# Evaluating AI-generated patient education materials for endometrial cancer surgery: a comparative analysis of response quality, reliability, and readability between ChatGPT and DeepSeek models

**DOI:** 10.3389/fpubh.2026.1831988

**Published:** 2026-08-26

**Authors:** Ling Tian, Long-yu Tang, Ming-tao Yang, Yan Wang, Hong-ni He, Tian-wen He, Ying Tang, Chuan Lin, Hui-quan Hu, Jun Li

**Affiliations:** 1 Department of Obstetrics and Gynecology, Beijing Anzhen Nanchong Hospital, Capital Medical University and Nanchong Central Hospital, Nanchong, Sichuan, China; 2 Department of Obstetrics and Gynecology, North Sichuan Medical College, Nanchong, Sichuan, China; 3 Department of Obstetrics and Gynecology, Women and Children's Hospital of Chongqing Medical University, Chongqing, China; 4 Department of Obstetrics and Gynecology, Chongqing Health Center for Women and Children, Chongqing, China; 5 Chongqing Research Center for Prevention & Control of Maternal and Child Diseases and Public Health, Chongqing, China; 6 Department of Obstetrics and Gynecology, Jinjiang District Maternal and Child Health Hospital of Chengdu, Chengdu, China

**Keywords:** artificial intelligence, ChatGPT, DeepSeek, endometrial cancer, health literacy, large language models (LLMs), patient education, readability

## Abstract

**Purpose:**

This study aimed to evaluate and compare the quality, reliability, and readability of patient education materials on endometrial cancer surgery generated by ChatGPT (GPT-5) and DeepSeek (R1).

**Materials and methods:**

This cross-sectional study analyzed the responses generated by ChatGPT and DeepSeek to totally 41 questions covering four domains: surgical planning, preoperative evaluation, postoperative care, and long-term follow-up. Reliability was assessed through the DISCERN and EQIP instruments, quality was evaluated by the Global Quality Score (GQS), and readability was analyzed by the Flesch Reading Ease Score (FRES), Gunning Fog Index (GFI), and Flesch-Kincaid Grade Level (FKGL). Statistical comparisons were performed by using paired *t*-tests and Wilcoxon signed-rank tests.

**Results:**

The two large language models (LLMs) generated education materials of comparable quality, as reflected in GQS scores (median: DeepSeek vs. ChatGPT 5.00 vs. 4.67, *p* = 0.077). DeepSeek demonstrated statistically significantly higher reliability scores on both DISCERN and EQIP instruments (both *p* < 0.001). Readability scores (FRES, GFI) were similar between groups, while DeepSeek exhibited a higher FKGL (10.28 vs. 8.84, *p* < 0.001), indicating the greater text complexity. Subgroup analysis showed that DeepSeek performed better in terms of reliability in the postoperative care and long-term follow-up domains, while ChatGPT exhibited better readability in the surgical planning domain.

**Conclusion:**

Both DeepSeek and ChatGPT can generate patient education text drafts that are commendable in their structural coherence and linguistic clarity. DeepSeek demonstrates a significant advantage in information reliability, particularly excelling in postoperative and follow-up management content. ChatGPT shows a slight edge in the readability of surgical planning sections. However, the text readability of both models exceeds the general public's health literacy level. This indicates that large language models can only serve as auxiliary tools for generating patient education materials. Their outputs must undergo review by clinical experts and readability optimization to ensure both accuracy and comprehensibility of the information.

## 1 Introduction

Endometrial cancer ranks as the second most prevalent malignancy of the female reproductive system worldwide and the third leading cause of gynecologic cancer-associated mortality (1, 2). In the United States, an estimated 69,120 new cases and 13,860 deaths were anticipated in 2025 (3). In China, there were 77,700 incident cases and 13,500 deaths attributable to endometrial cancer in 2022. Over the past decade, the global incidence has been rising annually, with an increasingly younger age at onset. Notably, in some economically developed cities, the incidence of endometrial cancer has surpassed that of cervical cancer (4).

Surgery remains the cornerstone of treatment for early-stage and select advanced-stage patients, and the decision-making process is complex, involving comprehensive consideration of surgical approaches, risks, benefits, and long-term outcomes (5). In this context, effective patient education has been suggested to enhance shared decision-making between clinicians and patients, reduce preoperative anxiety, improve treatment adherence, and facilitate postoperative recovery (6). Shared decision-making (SDM) is a collaborative process in which clinicians and patients share information regarding available treatment options and individual preferences, with the goal of reaching a shared, informed decision. It is widely recognized as a cornerstone of person-centered oncology care (7). Evidence indicates that SDM enhances patient knowledge, improves the accuracy of risk perception, and increases satisfaction, while concurrently reducing decisional conflict. In the specific context of endometrial cancer, qualitative research has revealed that women frequently contend with navigate complex information needs throughout their diagnostic and treatment trajectories, underscoring the critical importance of accessible and contextually sensitive educational resources (8). However, as the clinical consultation time is limited and there are insufficient standardized education materials to meet individual patient needs, patients often turn to the internet for health-related information (9). Data from the Health Information National Trends Survey (HINTS) show that the proportion of cancer survivors actively seeking cancer-related information rose from 66.8% in 2003 to 80.8% in 2013 (10). A subsequent cross-sectional analysis of HINTS data in 2024 further revealed that approximately 74% of adult cancer survivors had actively sought health information online within the year preceding the survey (11).

While online resources are easily accessible, the quality of available information is inconsistent—often inaccurate, outdated, commercially biased, or written in overly technical language that exceeds the public's health literacy level. These shortcomings may mislead patients and adversely influence their treatment decisions and prognosis (12, 13). To address these limitations, there is a growing need to develop accessible on-demand sources of accurate, comprehensible, and personalized health information.

The rise of large language models (LLMs), represented by ChatGPT, offers a potential solution for generating accurate, comprehensible, and personalized health information on demand (14). The application of LLMs to generate patient education materials in oncology has attracted growing research interest. Previous studies report that GPT-4 can produce materials with comparable or superior readability to that of existing resources and generally acceptable accuracy (15, 16). However, the rapid integration of these technologies into clinical settings has also raised concerns regarding factual reliability, information completeness, and the risk of generating content that, although structurally coherent, may contain subtle inaccuracies or fail to adequately address the full range of patient concerns (17–19). Their rapid adoption in the medical question-answering domain has sparked extensive research interest. Preliminary evidence has suggested that ChatGPT demonstrates impressive accuracy in answering certain medical questions (20), but it also exhibits significant limitations in other areas, including “hallucination” (generating fabricated content), reliance on outdated knowledge, and a lack of deep understanding of complex clinical contexts (21). Notably, existing literature predominantly focuses on evaluating single mainstream models (e.g., ChatGPT), while systematic comparative studies on emerging, efficient, and architecturally distinct models (e.g., the open-source model DeepSeek, which emphasizes reasoning and cost-effectiveness) in specific clinical scenarios are lacking (22). Particularly, in the highly specialized subspecialty of gynecologic oncology concerning endometrial cancer surgery, where information accuracy is paramount, the performance differences in generating patient education materials across various LLMs remain unknown.

To address this gap, this study aimed to conduct a preliminary exploratory evaluation of the ChatGPT (GPT-5 version) and DeepSeek (R1 version) models in generating patient education materials related to endometrial cancer surgery. The evaluation focused on the overall response quality, objectivity, comprehensiveness of medical information, and readability of the model outputs. In addition, this study examined the performance of each model in addressing a wide range of patient concerns across four key domains: (1) surgical options and technical considerations, (2) preoperative evaluation and optimization, (3) postoperative care and complication management, and (4) long-term follow-up and disease management. Through this comparative analysis, we intend to explore the potential and limitations of LLMs in enhancing patient education for endometrial cancer.

## 2 Materials and methods

### 2.1 Study design and question selection

**Figure 1:**
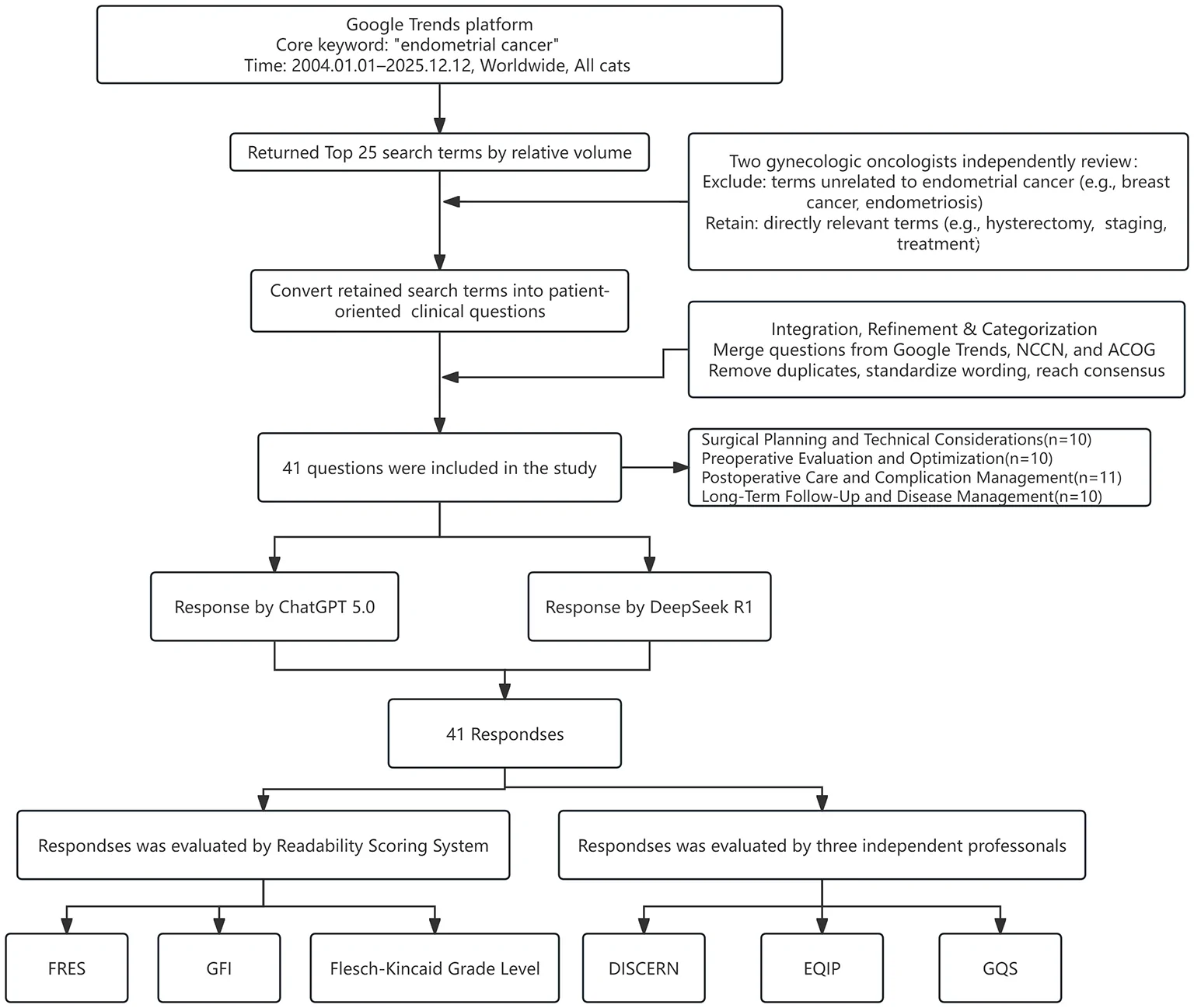
Flowchart of question selection and response evaluation process.

This cross-sectional study sought to evaluate the response performance of ChatGPT and DeepSeek on questions commonly posed by patients undergoing surgery for endometrial cancer. To construct a clinically relevant question bank, a systematic, multi-source strategy was employed. First, to capture real-world patient information needs, Google Trends (https://trends.google.com/) were utilized to identify high-frequency search queries related to endometrial cancer. Using “endometrial cancer” as the core keyword, a global search from January 1, 2004, to December 12, 2025, was conducted, with all categories selected to maximize retrieval breadth. This process yielded the top 25 search terms ranked by relative search volume (Supplementary Table S1). These 25 terms were then independently reviewed by two board-certified gynecologic oncology specialists. Terms not directly pertinent to the clinical management of endometrial cancer, including those referring to other malignancies (e.g., “breast cancer,” and “ovarian cancer”) or benign conditions (e.g., “endometriosis”), were excluded, whereas entries directly related to the disease and its treatment (e.g., “hysterectomy,” “endometrial cancer staging,” and “endometrial cancer treatment”) were retained. Afterwards, these high-frequency search queries were converted into potential patient-oriented clinical questions. In parallel, authoritative patient education resources, including the National Comprehensive Cancer Network (NCCN) ^*^Patient Guide: Uterine Cancer^*^ and patient education materials from the American College of Obstetricians and Gynecologists (ACOG), were comprehensively reviewed. Moreover, key educational themes and patient concerns were extracted from these sources. The same two specialists then integrated, refined, and de-duplicated all candidate questions. Through iterative discussion and consensus, the initial question bank was streamlined and organized into a final set of 41 items. These 41 questions were systematically grouped into four clinical domains aligned with the patient care trajectory: (1) surgical planning and technical considerations; (2) preoperative evaluation and optimization; (3) postoperative care and complication management; and (4) long-term surveillance and disease management. The original replies generated by ChatGPT (GPT-5) and DeepSeek (R1) for all 41 predefined questions are presented in Supplementary Table S2. Figure 1 presents a flowchart of the question inclusion and response evaluation process.

### 2.2 Artificial intelligence (AI) models and query protocol

The two LLMs examined in this study were ChatGPT and DeepSeek. The version of ChatGPT evaluated was GPT-5 (model version gpt-5-2025-08-07), which was officially launched by OpenAI on August 7, 2025, and accessed via its public web interface at chat.openai.com. As for DeepSeek, the version evaluated was DeepSeek-R1 (model version DeepSeek-R1-0528), which was released by DeepSeek on January 20, 2025, and accessed through its official web interface. In both cases, the standard consumer-facing versions of the two platforms available at the time of data collection were used.

All queries were executed within a defined window (December 10-20, 2025) to reduce the risk of confounding effects introduced by model updates over the course of data collection. To promote response consistency, each of the 41 questions was entered individually into both ChatGPT and DeepSeek using the “New Chat” function, thereby simulating a “zero-shot” prompting scenario and eliminating any contextual memory effects. For both platforms, the default generation settings supplied by their respective web interfaces were employed, without adjusting any configurable parameters such as temperature or top-p sampling. By adhering to the default configurations of each platform, our query protocol was intended to replicate the experience of ordinary end users seeking health information through these publicly accessible tools.

### 2.3 Ethical considerations

No human subjects, patient data, or clinical interventions were involved in this study. It constituted a secondary analysis of AI-generated content and did not involve any identifiable personal information, therefore, ethical approval and informed consent were not required.

### 2.4 Assessment tools and evaluation process

The quality and readability of the generated text were evaluated using multiple established metrics, such as the Flesch-Kincaid Grade Level (FKGL), the Flesch Reading Ease Score (FRES), the Gunning Fog Index (GFI), the DISCERN instrument, the Global Quality Score (GQS), and the Ensuring Quality Information for Patients (EQIP) tool. These metrics were selected to comprehensively assess the reliability, readability, quality, and comprehensibility of patient education materials, which are essential for supporting informed healthcare decision-making. Three extensively used metrics were employed in this study, including FRES (the FRES ranges from 0 to 100, with higher scores indicating easier readability) (23), GFI (it indicates the years of formal education required to understand the text) (24), and FKGL (it estimates the U.S. educational grade level required for text comprehension).

The DISCERN instrument, is a commonly used validated tool for assessing the quality of consumer health information (25). The DISCERN scale involves 16 items for evaluating the quality of written health information, focusing on reliability, treatment choices, and overall quality. The EQIP tool is a 20-item checklist that assesses the quality of information content, structure, and identifies data (26).

The overall quality of responses was assessed using the GQS. This score utilizes a validated 5-point scale, where higher scores indicate superior quality, ranging from 1 (poor) to 5 (excellent) (27).

### 2.5 Evaluation process

An enhanced readability scoring system (https://readabilityformulas.com/readability-scoring-system.php) automated formula was applied to the plain text of each generated response (with model-specific greetings and non-informative phrasing removed) to calculate the objective FKGL, FRES, and GFI scores. The DISCERN scores, GQS scores, and EQIP tool ratings for all responses were completed by three independent evaluators, all of whom are board-certified gynecologic oncology surgeons with extensive clinical and academic experience.

Three members of the author team (HQ.H, JL, and CL) were selected to serve as assessors, based on their expertise in gynecologic oncology and their prior involvement in the development of patient education resources. To mitigate the potential influence of institution-specific practice patterns on the evaluations, the assessors were recruited from three distinct tertiary medical centers (more details are available in institutional affiliations in the author list), thereby ensuring a breadth of clinical perspectives. All three assessors had previously applied the DISCERN and GQS instruments in studies evaluating health information quality, and two of them had also utilized the EQIP tool in prior research on patient education materials.

Before formal evaluation, a calibration exercise was carried out to standardize the assessors' interpretation of scoring criteria and to improve the inter-rater consistency. In this exercise, five AI-generated responses, which covered topics related to endometrial cancer but not drawn from the final set of 41 questions, were independently rated by each assessor using all the three evaluation tools. Later, the assessors discussed scoring discrepancies, clarified ambiguous items, and reached a consensus on how each criterion should be applied. This process proved to be especially valuable for the DISCERN instrument, given that items related to treatment options and SDM required careful contextual interpretation within the surgical setting. Following the calibration exercise, the intraclass correlation coefficient (ICC) for pre-scoring exceeded 0.75, indicating satisfactory inter-rater agreement prior to the start of formal data collection.

The formatting and identifying information was removed in patient education materials to ensure source anonymity. To minimize potential bias, all responses were anonymized and coded prior to evaluation, and the model source (ChatGPT or DeepSeek) was not disclosed to the evaluators. Each evaluator received the question-and-answer sets in a different random order and independently assessed each response according to the predefined criteria. A random number generator was used to scramble the order of the materials before distributing them to the reviewers.

### 2.6 Statistical analysis

All statistical analyses were performed using R software (version 4.3.0). Descriptive statistics were calculated for all continuous variables. The normality of data was assessed by the Shapiro–Wilk test. Readability scores (FRES, GFI, FKGL), which were approximately normally distributed, were compared between the two models using two-sample *t*-tests (with Welch's *t*-test conducted for potential unequal variances). Results were reported as mean ± standard deviation (SD). For ordinal scale data obtained by the DISCERN, EQIP, and GQS tools, which were not normally distributed, the non-parametric Wilcoxon signed-rank test was employed for model comparisons. These results were presented as median with interquartile range (Q1, Q3). Stratified analyses were performed for each of the four question categories by the same comparative tests (paired *t*-tests for readability scores and Wilcoxon tests for quality scores). Inter-rater reliability among the three evaluators was assessed using a two-way random-effects model, absolute agreement, and single rater intra-class correlation coefficient (ICC). An ICC value greater than 0.75 was considered indicative of good reliability. A two-sided *p*-value < 0.05 was considered statistically significant.

## 3 Results

### 3.1 Comparison of overall readability and comprehensibility

No statistically significant differences were observed between ChatGPT and DeepSeek in FRES readability scores (47 ± 11 vs. 47 ± 12) or GFI difficulty indices (11.80 ± 1.63 vs. 12.40 ± 1.78; *p* > 0.9 and ^*^p^*^ = 0.12, respectively). However, the FKGL of the text generated by DeepSeek (10.28 ± 2.10) was significantly higher than that created by ChatGPT (8.84 ± 1.48), indicating the greater linguistic complexity (mean difference −1.4, 95% CI [−2.2, −0.64], *p* < 0.001; Table 1).

**Table 1:** Comparison of ChatGPT and DeepSeek on FRES, GFI, and Flesch–Kincaid grade level.

Characteristic	ChatGPT *N* = 41	DeepSeek *N* = 41	Difference^a^	95% CI^a^	*p*-value^a^
FRES, mean ± SD	47 ± 11	47 ± 12	−0.20	−5.1, 4.7	>0.9
GFI, mean ± SD	11.80 ± 1.63	12.40 ± 1.78	−0.60	−1.3, 0.15	0.12
Flesch–Kincaid grade level, mean ± SD	8.84 ± 1.48	10.28 ± 2.10	−1.4	−2.2, −0.64	< 0.001

^a^Welch two sample *t*-test.

CI, confidence interval.

### 3.2 Inter-rater reliability

To evaluate the consistency of scores assigned by the three independent raters, the ICC values were calculated using a two-way random effects model with absolute agreement and a single-rater assessment design. Based on raw scoring data for all the 41 questions across both models, the single-rater ICC values for the three instruments are summarized in Table 2. The single-rater ICC values for the three assessment instruments were as follows: DISCERN, 0.86 (95% CI: 0.82–0.90); EQIP, 0.84 (95% CI: 0.78–0.88); and GQS, 0.82 (95% CI: 0.76–0.87). All ICC values exceeded the predetermined threshold of 0.75, indicative of good to excellent inter-rater reliability for each of the three instruments.

**Table 2:** Inter-rater reliability of the three assessment instruments.

Assessment instrument	ICC (single-rater)	95% confidence	Interval reliability level
DISCERN	0.86	0.81–0.90	Good to excellent
EQIP	0.84	0.78–0.88	Good to excellent
GQS	0.82	0.76–0.87	Good to excellent

ICC, intraclass correlation coefficient; EQIP, ensuring quality information for patients; GQS, global quality score.

### 3.3 Evaluation of information quality and tool practicality

DeepSeek yielded significantly higher scores on both DISCERN and EQIP instruments (*p* < 0.001). For example, the overall median (Q1, Q3) for DISCERN was 59 (57, 64) vs. 54 (52, 59), and that for EQIP was 78.3 (75.8, 83.6) vs. 73.3 (70.2, 75.8). However, in the overall quality score GQS, there was no significant difference in the overall rating between the two (median: 5.00 vs. 4.67, *p* = 0.077), though differences existed in individual evaluator scores (Table 3).

**Table 3:** Comparison of ChatGPT and DeepSeek on DISCERN, EQIP, and GQS.

Characteristic	ChatGPT *N* = 41	DeepSeek *N* = 41	Difference^a^	95% CI^a^	*p*-value^a^
DISCERN score					
Rater 1 median (Q1, Q3)	52 (50, 59)	58 (55, 62)	−6.4	−9.8, −3.1	< 0.001
Rater 2, mean ± SD	57 ± 8	64 ± 6	−7.1	−10, −4.1	< 0.001
Rater 3, median (Q1, Q3)	53 (52, 55)	57 (56, 58)	−5.2	−7.7, −2.8	< 0.001
15.6-7.4,-1.3498ptOverall, median (Q1, Q3)	54 (52, 59)	59 (57, 64)	−6.3	−8.7, −3.8	< 0.001
EQIP score					
Rater 1, median (Q1, Q3)	57.5 (57.5, 60.0)	60.0 (57.5, 68.4)	−4.3	−6.5, −2.0	< 0.001
Rater 2, median (Q1, Q3)	70 (68, 80)	85 (80, 90)	−9.9	−13, −6.5	< 0.001
Rater 3, median (Q1, Q3)	85.0 (82.5, 87.5)	92.5 (90.0, 92.5)	−6.4	−7.7, −5.1	< 0.001
15.6-7.4,-1.3498ptOverall, median (Q1, Q3)	73.3 (70.2, 75.8)	78.3 (75.8, 83.6)	−6.9	−8.6, −5.2	< 0.001
GQS score					
Rater 1, median (Q1, Q3)	5.00 (5.00, 5.00)	5.00 (4.00, 5.00)	0.00	−0.21, 0.21	>0.9
Rater 2, median (Q1, Q3)	5.00 (4.00, 5.00)	5.00 (5.00, 5.00)	−0.12	−0.33, 0.09	0.3
Rater 3, median (Q1, Q3)	4.00 (4.00, 5.00)	5.00 (4.00, 5.00)	−0.32	−0.56, −0.08	0.010
Overall, median (Q1, Q3)	4.67 (4.33, 4.67)	5.00 (4.33, 5.00)	−0.15	−0.31, 0.02	0.077

^a^Welch Two Sample *t*-test.

CI, confidence interval.

### 3.4 Comparison of information readability between ChatGPT and DeepSeek across question domains

A stratified analysis according to question type was conducted, which revealed that the performance of the two models in terms of the FRES, GFI, and Flesch-Kincaid Grade Level varied depending on the question type (Table 4): In Surgical Planning and Technical Considerations: the GFI score of DeepSeek (13.47 ± 0.54) was significantly higher than that of ChatGPT (11.91 ± 1.00), indicating the greater text difficulty (*p* < 0.001). The Flesch-Kincaid Grade Level of DeepSeek was also significantly higher (11.40 ± 0.83 vs. 9.25 ± 0.94, *p* < 0.001). No significant difference was detected in FRES scores (*p* = 0.2). In Preoperative Evaluation and Optimization: There were no significant differences between the two models across all three readability metrics (FRES *p* = 0.4, GFI *p* = 0.8, Flesch–Kincaid *p* = 0.10), indicating comparable readability performance in this question category. In postoperative care and complication management: there were no significant differences between the two models in terms of FRES, GFI, or Flesch–Kincaid scores (all *p* > 0.05), indicating similar readability. In long-term follow-up and disease management: DeepSeek demonstrated a significantly higher Flesch–Kincaid Grade Level (10.92 ± 2.73) than ChatGPT (8.64 ± 1.94, *p* = 0.046). FRES and GFI scores did not show any significant difference between the two models (*p* = 0.5 and *p* = 0.2).

**Table 4:** Comparison of ChatGPT and DeepSeek across question domains on FRES, GFI, and Flesch-Kincaid Grade Level.

Characteristic	Surgical planning and technical considerations, *N* = 20					Preoperative evaluation and optimization, *N* = 20					Postoperative Care and Complication Management, *N* = 22					Long-Term Follow-Up and Disease Management, *N* = 20				
	ChatGPT *N*= 10	DeepSeek *N* = 10	Difference^a^	95%CI^a^	*p*-value^a^	ChatGPT *N* = 10	DeepSeek *N*= 10	Difference^a^	95%CI^a^	*p*-value^a^	ChatGPT *N* = 11	DeepSeek *N* = 11	Difference^a^	95%CI^a^	*p*-value^a^	ChatGPT *N* = 10	DeepSeek *N*= 10	Difference^a^	95%CI^a^	*p*-value^a^
FRES, mean ± SD	45.4 ± 6.3	42.5 ± 4.0	2.9	−2.1, 7.9	0.2	44.0 ± 8.6	47.0 ± 5.7	−3.0	−9.9, 3.9	0.4	51 ± 11	56 ± 12	−5.2	−16, 5.4	0.3	47 ± 14	42 ± 16	5.0	−9.2, 19	0.5
GFI, mean ± SD	11.91 ± 1.00	13.47 ± 0.54	−1.6	−2.3, −0.79	< 0.001	12.15 ± 1.20	12.28 ± 0.95	−0.13	−1.2, 0.89	0.8	11.25 ± 1.85	10.93 ± 2.07	0.33	−1.4, 2.1	0.7	11.95 ± 2.22	13.07 ± 1.91	−1.1	−3.1, 0.83	0.2
Flesch–Kincaid grade level, mean ± SD	9.25 ± 0.94	11.40 ± 0.83	−2.2	−3.0, −1.3	< 0.001	9.34 ± 1.17	10.31 ± 1.29	−0.97	−2.1, 0.19	0.10	8.21 ± 1.55	8.65 ± 2.04	−0.44	−2.1, 1.2	0.6	8.64 ± 1.94	10.92 ± 2.73	−2.3	−4.5, −0.05	0.046

^a^Welch two sample *t*-test.

CI, confidence interval.

### 3.5 Comparison of information quality between ChatGPT and DeepSeek across question domains

The quality of information provided by ChatGPT and DeepSeek across four surgical question categories was analyzed using three validated instruments: DISCERN, EQIP, and GQS. The results are summarized in Table 5. In the surgical planning and technical considerations category, the overall median DISCERN score of ChatGPT (59.83, IQR: 59.00–61.33) was higher than that of DeepSeek (57.17, IQR: 56.67–58.33), with a statistically significant difference of 2.2 points (95% CI: 0.35–4.1, *p* = 0.024). Inter-rater variability was noted: Rater 2 reported a mean score significantly higher for ChatGPT (64.6 vs. 57.1, *p* < 0.001), while Raters 1 and 3 showed no significant differences. With regard to preoperative evaluation and optimization, the overall median score was lower for ChatGPT (55.50, IQR: 54.33–56.33) than DeepSeek (57.67, IQR: 56.33–58.67), with a difference of−3.1 points (95% CI: −5.4 to −0.77, *p* = 0.012). Rater 3 also reported a significant advantage for DeepSeek (*p* < 0.001). In Postoperative Care and Complication Management, DeepSeek scored significantly higher than ChatGPT across all three raters, and the overall median score was 59.3 (IQR: 57.7–60.7) vs. 50.3 (IQR: 49.7–52.7), with a difference of −8.9 points (95% CI: −12 to −6.0, *p* < 0.001). Similarly, for long-term follow-up and disease management, DeepSeek demonstrated superior performance with a median score of 72 (IQR: 72–73) relative to that of 54 (IQR: 52–64) for ChatGPT, a difference of −15 points (95% CI: −20 to −10, *p* < 0.001).

**Table 5:** Comparison of information quality in different question types.

Characteristic	Surgical planning and technical considerations, *N* = 20					Preoperative evaluation and optimization, *N* = 20					Postoperative care and complication management, *N* = 22					Long-term follow-up and disease management, *N* = 20				
	ChatGPT *N* = 10	DeepSeek *N* = 10	Difference^a^	95%CI^a^	*p*-value^a^	ChatGPT *N* = 10	DeepSeek *N* = 10	Difference^a^	95%CI^a^	*p*-value^a^	ChatGPT *N* = 11	DeepSeek *N* = 11	Difference^a^	95%CI^a^	*p*-value^a^	ChatGPT *N* = 10	DeepSeek *N* = 10	Difference^a^	95%CI^a^	*p*-value^a^
DISCERN																				
Rater 1, median (Q1, Q3)	60.0 (59.0, 61.0)	59.0 (58.0, 61.0)	−1.0	−4.2, 2.2	0.5	50.0 (48.0, 55.0)	53.0 (52.0, 58.0)	−3.1	−6.5, 0.26	0.068	52.00 (50.00, 52.00)	57.00 (55.00, 57.00)	−6.7	−12, −1.8	0.012	56 (52, 70)	75 (73, 78)	−15	−22, −7.7	< 0.001
Rater 2, mean ± SD	64.6 ± 2.3	57.1 ± 2.3	7.5	5.3, 9.7	< 0.001	62.10 ± 3.67	62.60 ± 2.17	−0.50	−3.4, 2.4	0.7	49 ± 6	64 ± 3	−15	−19, −10	< 0.001	52 ± 1	72 ± 3	−20	−22, −18	< 0.001
Rater 3, median (Q1, Q3)	56.00 (55.00, 57.00)	56.00 (56.00, 57.00)	0.20	−2.3, 2.7	0.9	51.50 (50.00, 53.00)	56.50 (56.00, 57.00)	−5.7	−7.9, −3.5	< 0.001	52.00 (51.00, 53.00)	57.00 (57.00, 57.00)	−5.5	−7.7, −3.4	< 0.001	54 (53, 69)	70 (68, 73)	−9.9	−17, −3.1	0.007
15.6-7.4,-26.3689.4ptOverall, median (Q1, Q3)	59.83 (59.00, 61.33)	57.17 (56.67, 58.33)	2.2	0.35, 4.1	0.024	55.50 (54.33, 56.33)	57.67 (56.33, 58.67)	−3.1	−5.4, −0.77	0.012	50.3 (49.7, 52.7)	59.3 (57.7, 60.7)	−8.9	−12, −6.0	< 0.001	54 (52, 64)	72 (72, 73)	−15	−20, −10	< 0.001
EQIP																				
Rater 1, median (Q1, Q3)	57.50 (55.00, 57.50)	57.50 (57.50, 60.00)	−2.0	−3.9, −0.14	0.037	57.50 (57.50, 60.00)	57.50 (57.50, 57.50)	1.0	0.08, 1.9	0.037	57.5 (57.5, 60.0)	65.8 (65.8, 68.4)	−8.1	−11, −5.5	< 0.001	59.0 (57.5, 68.4)	68.4 (68.4, 73.7)	−7.6	−12, −2.9	0.004
Rater 2, median (Q1, Q3)	80.0 (78.0, 80.0)	75.0 (71.0, 76.0)	3.5	−0.15, 7.1	0.059	81.3 (80.0, 85.0)	85.0 (80.0, 85.0)	−3.0	−7.3, 1.3	0.2	65 (63, 68)	85 (80, 90)	−18	−26, −11	< 0.001	69 (68, 69)	90 (90, 90)	−21	−23, −19	< 0.001
Rater 3, median (Q1, Q3)	90.00 (90.00, 92.50)	90.00 (90.00, 92.50)	−0.60	−3.3, 2.1	0.6	82.5 (80.0, 85.0)	91.3 (90.0, 92.5)	−8.0	−10, −5.8	< 0.001	82.5 (82.5, 85.0)	92.5 (92.5, 92.5)	−8.4	−9.8, −7.0	< 0.001	87.5 (85.0, 87.5)	95.0 (95.0, 95.0)	−8.5	−9.4, −7.6	< 0.001
Overall, median (Q1, Q3)	75.33 (74.17, 76.67)	74.75 (73.67, 75.83)	0.30	−1.6, 2.2	0.7	74.58 (73.33, 75.83)	77.92 (75.83, 78.33)	−3.3	−5.4, −1.3	0.003	68.3 (67.7, 71.8)	82.0 (79.4, 82.8)	−12	−14, −8.7	< 0.001	71.9 (71.3, 73.8)	85.3 (84.5, 86.2)	−12	−14, −11	< 0.001
	ChatGPT *N* = 10	DeepSeek *N* = 10	Difference^a^	95%CI^a^	*p*-value^a^	ChatGPT *N* = 10	DeepSeek *N* = 10	Difference^a^	95%CI^a^	*p*-value^a^	ChatGPT *N* = 11	DeepSeek *N* = 11	Difference^a^	95%CI^a^	*p*-value^a^	ChatGPT *N* = 10	DeepSeek *N* = 10	Difference^a^	95%CI^a^	*p*-value^a^
GQS score																				
Rater 1, median (Q1, Q3)	5.000 (5.000, 5.000)	5.000 (5.000, 5.000)	−0.10	−0.33, 0.13	0.3	5.00 (4.00, 5.00)	5.00 (5.00, 5.00)	−0.10	−0.53, 0.33	0.6	5.00 (5.00, 5.00)	4.00 (4.00, 5.00)	0.55	0.06, 1.0	0.029	4.50 (4.00, 5.00)	5.00 (5.00, 5.00)	−0.40	−0.81, 0.01	0.058
Rater 2, median (Q1, Q3)	5.00 (4.50, 5.00)	5.00 (5.00, 5.00)	−0.05	−0.45, 0.35	0.8	5.00 (5.00, 5.00)	5.00 (5.00, 5.00)	−0.10	−0.45, 0.25	0.6	4.00 (4.00, 5.00)	5.00 (4.00, 5.00)	−0.50	−0.98, −0.02	0.042	5.00 (5.00, 5.00)	5.00 (4.00, 5.00)	0.20	−0.19, 0.59	0.3
Rater 3, median (Q1, Q3)	5.00 (4.00, 5.00)	5.00 (5.00, 5.00)	−0.20	−0.59, 0.19	0.3	4.00 (4.00, 4.00)	5.00 (4.00, 5.00)	−0.80	−1.2, −0.41	< 0.001	4.00 (4.00, 4.00)	4.00 (4.00, 4.00)	0.00	−0.40, 0.40	>0.9	4.00 (4.00, 5.00)	5.00 (4.00, 5.00)	−0.30	−0.87, 0.27	0.3
Overall, median (Q1, Q3)	4.92 (4.67, 5.00)	5.00 (5.00, 5.00)	−0.12	−0.35, 0.12	0.3	4.67 (4.33, 4.67)	4.83 (4.67, 5.00)	−0.33	−0.58, −0.09	0.010	4.33 (4.33, 4.67)	4.33 (4.00, 4.67)	0.02	−0.34, 0.37	>0.9	4.67 (4.33, 4.67)	5.00 (4.33, 5.00)	−0.17	−0.53, 0.20	0.3

^a^Welch two sample *t*-test.

CI, confidence interval.

The EQIP scores revealed a similar pattern. In Surgical Planning, there was no significant overall difference between the two models (median: 75.33 vs. 74.75, *p* = 0.7). However, in preoperative evaluation, DeepSeek scored higher (median: 77.92 vs. 74.58, *p* = 0.003). In postoperative care and long-term follow-up, DeepSeek significantly outperformed ChatGPT, with the overall median EQIP scores of 82.0 vs. 68.3 (*p* < 0.001) and 85.3 vs. 71.9 (*p* < 0.001), respectively.

The GQS results were mixed across question types. In surgical planning, no significant overall difference was detected (*p* = 0.3). For preoperative evaluation, ChatGPT demonstrated slightly lower scores than DeepSeek (median: 4.67 vs. 4.83, *p* = 0.010). In Postoperative Care, no significant difference was observed (*p* > 0.9), while in long-term follow-up, the difference did not reach statistical significance (*p* = 0.3).

## 4 Discussion

This exploratory study evaluated the performance of DeepSeek and ChatGPT in generating patient education materials for endometrial cancer surgery. Both models produced well-structured, informative, and qualitatively reliable text, with overall comparable readability. Nevertheless, DeepSeek demonstrated a significant advantage in information reliability, and this superiority showed a clear domain specificity. Moreover, a persistent and noteworthy finding was that the reading grade levels of text generated by both models substantially exceeded the health literacy level of the general public, posing a potential obstacle to clinical translation.

The potential of AI chatbots to deliver accurate and comprehensible health information has attracted increasing research interest in their medical applications. Our findings align with previous studies evaluating LLMs in various surgical fields (28–33), confirming that both DeepSeek and ChatGPT are capable of generating well-structured and informative text for patient education in endometrial cancer.

Previous research has yielded divergent findings regarding which model generates superior education materials (28, 34). Using the validated DISCERN and EQIP instruments (35), our results align with the latter study, demonstrating that DeepSeek significantly outperformed ChatGPT in terms of information reliability. In the evaluation of information quality, DeepSeek scored significantly higher than ChatGPT in both the DISCERN and EQIP tools, indicating that its generated content showed greater transparency of information sources, better balance, and improved structural clarity. Specifically, ChatGPT tended to produce responses that were concise and direct but often lacked sufficient detail (36). In contrast, DeepSeek generated extremely detailed responses, elaborating on various subsections of the content. When providing diagnostic or treatment recommendations, DeepSeek aimed to offer as specific a plan as possible, thereby enhancing the feasibility of its responses (37). For example, when answering questions about “pelvic lymphedema management” or “recurrence surveillance plans,” DeepSeek consistently provided detailed, step-by-step, phased guidance. This structural approach naturally aligns with the DISCERN and EQIP criteria for “information completeness” and “structural clarity.” In comparison, while accurate responses were generated by ChatGPT, they were more general and sometimes failed to exhaustively list all management options or provide specific action recommendations.

Noteworthily, this study discovered that model performance was context-dependent. Subgroup analysis revealed clear domain specificity: DeepSeek performed particularly well in the highly structured clinical management domains of “postoperative care and complication management” and “long-term follow-up and disease management” (with differences in DISCERN and EQIP scores both reaching *p* < 0.001), whereas ChatGPT demonstrated better readability GFI in the area of “surgical planning and technical considerations.” This finding echoes recent research comparing DeepSeek and GPT in the field of head and neck surgery, indicating that model performance is highly dependent on the task domain and evaluation dimension (38). We hypothesize that this domain-specific divergence may stem, at least in part, from the distinct training and optimization paradigms underlying the two models. To be specific, DeepSeek operates on a rule-reinforced learning framework that emphasizes structured reasoning and stepwise logical deduction, an architecture that is suspected to be particularly conductive to generating systematic, procedure-oriented clinical knowledge, such as postoperative care protocols and follow-up surveillance schedules. In contrast, ChatGPT is developed using reinforcement learning from human feedback (RLHF), which prioritizes conversational fluency, readability, and user engagement. This design orientation may confer an advantage in conveying decision-oriented knowledge that involves weighing options and trade-offs (for example, in the context of surgical planning), but could potentially come at the expense of completeness in structured information content. However, we wish to underscore that these mechanistic interpretations remain speculative, as the present study did not directly examine the internal architectural operations or training dynamics of these two models. The observed differences in their performance may also be attributable to other unmeasured factors, including variations in the composition and temporal currency of their training corpora, differences in fine-tuning strategies, or even stochastic variability intrinsic to the generation process. Accordingly, these explanatory accounts should be viewed as working hypotheses intended to guide future research, rather than as definitive conclusions. Rigorous studies that employ controlled experimental designs—such as systematic probing of model outputs under standardized input perturbations, or direct comparison of model-generated content against human-written standards using identical knowledge bases—are warranted to validate the hypothesized mechanism-outcome relationships. This supports the emerging view that the significant heterogeneity of outcomes across different question types emphasizes that the evaluation of LLMs must delve into specific clinical subdomains rather than remaining at the level of general conclusions. This provides novel insights in improving the performance of LLMs in generating patient education materials.

Readability is a crucial concern in evaluating AI-generated medical information. FKGL and FRES serve as the efficient and standardized tools for measuring text readability and hold reference value for assessing AI-generated health content (29). Previous research has indicated that DeepSeek shows a lower reading difficulty level in its responses than ChatGPT (37). However, in the present study, the two models performed comparably on the FRES and GFI, with no statistically significant difference (*p* > 0.9). Nonetheless, DeepSeek demonstrated a significantly higher FKGL than ChatGPT (10.28 vs. 8.84, *p* < 0.001), suggesting higher syntactic complexity in its text. This apparent discrepancy stems from the different emphases of the readability metrics: FRES and GFI primarily assess word- and sentence-level fluency, whereas FKGL is more sensitive to syntactic complexity. DeepSeek tended to employ more complex and nested sentences to accommodate for its higher reliability scores and poorer performance on FKGL. Notably, ChatGPT exhibited better GFI readability in the “surgical planning and technical considerations” domain, which involves weighing options and explaining clinical decisions, suggesting that it may prioritize concise and clear expression when processing decisional knowledge.

One of the most noteworthy findings of this study is that the reading grade levels of content generated by both models ranged from 8th−10th grade, significantly higher than the 6th−8th grade target recommended by the American Medical Association (AMA) for patient education materials. This reveals an inherent limitation of current general-purpose LLMs when applied in specialized medical fields. The possible reasons are shown as follows. First, the higher reading grade level may stem from the high proportion of academic literature and professional documents in the training data of models, leading them to replicate the narrative style of their “knowledge sources” rather than actively performing “health literacy adaptation.” This indicates that even the most advanced LLMs today are highly likely to produce default outputs that exceed the health literacy level of the general public. For patient populations with a lower health literacy level, this content may be difficult to understand, thus failing to serve its educational purpose. If this issue is not systematically addressed, AI-assisted educational tools may inadvertently exacerbate rather than alleviating existing health information inequities—because they are more readily utilized by groups with higher health literacy, while those most in need of help are left behind.

This study reveals that in the specialized domain of endometrial cancer surgery education, there is no absolute “best model,” but only the tool “most suitable for a specific task.” DeepSeek-generated content was significantly superior in information completeness and structural clarity (DISCERN/EQIP scores). Clinicians can prioritize its use as a drafting assistant for standardized discharge instructions, rehabilitation plans and follow-up reminders, to improve documentation efficiency and standardization. ChatGPT performed better in the readability of content related to “surgical planning,” with more concise outputs closer to patients' cognitive habits. Therefore, in scenarios requiring communication with patients about surgical risks and weighing treatment options, drafts generated by ChatGPT may be more convenient for doctors to personalize and explain. The subgroup analysis in this study found that DeepSeek did not have a universal reliability advantage, instead, it was highly concentrated in the “postoperative care” and “long-term follow-up” domains. Future development of specialized AI assistants for “clinical pathway management” or “chronic disease follow-up” can draw on the technical approach of DeepSeek (rule-reinforced learning). Notably, since the present study did not incorporate clinical safety verification, any speculation regarding the optimal use scenarios for the model should be regarded as hypothesis-generating rather than practice-guiding. Our findings suggest that DeepSeek may offer advantages in organizing domain-specific knowledge, whereas ChatGPT appears to excel in conciseness. Nonetheless, these observed differences must be confirmed through prospective studies that incorporate patient outcomes. Although the two models showed no difference in most readability metrics, their reading grade levels far exceeded the AMA-recommended 6th−8th grade target. This finding highlights a clinical tension: the more reliable and comprehensive information may be associated with the more complex expression, making it harder for patients with a lower health literacy level to understand. Therefore, for frontline clinicians: any AI-generated material, regardless of its quality, must undergo “readability downgrading” (e.g., splitting long sentences, explaining terminology, and adding illustrations) before it can be truly used for patient education.

When our findings were placed in a broader public health context, the persistent readability gap we observed—with both models generating content at an 8th- to 10^th^-grade reading level—raises significant equity concerns. Health literacy has been increasingly recognized not merely as an individual capability, but as a systemic determinant shaped by the complexity of health information and the accessibility of educational resources (39). Patients with limited health literacy face compounded obstacles throughout the cancer care continuum, from comprehending complex treatment options to adhering to postoperative surveillance regimens. When AI-generated educational materials default to language that exceeds the health literacy level of the general public, these tools may be associated with a risk of inadvertently exacerbating existing disparities—benefiting those with higher health literacy while failing those most in need of accessible information (40). This concern is especially pertinent in endometrial cancer, where the affected patient population tends to be older and may present with lower average health literacy levels (8). As LLMs become increasingly integrated into clinical practice, deliberate strategies are needed to tailor their outputs to diverse patient populations through plain language rewriting, visual supplementation, and culturally tailored content. The health literacy universal precautions framework, which advocates assuming that all patients may struggle with health information and proactively simplifying communication accordingly, offers a valuable guiding principle for such adaptations.

Moreover, evaluation of AI-generated patient education materials must extend beyond structural quality indicators to encompass patient-centered outcomes. Although our study assessed the potential quality of these materials from the perspective of clinical expert reviewers, the ultimate measure of their value lies in their real-world impact on patient knowledge acquisition, decision satisfaction, treatment adherence, and clinical outcomes. Emerging evidence suggests that LLMs can enhance patient comprehension and support SDM when appropriately deployed, yet these benefits are contingent upon the accessibility of the materials to their intended audience (41, 42). Future investigations should integrate patient-reported outcome measures and usability testing across diverse populations to determine whether AI-generated resources can truly fulfill their educational purpose in clinical practice.

There are several limitations in this study. First, from a methodological perspective, the evaluation was based on simulated static question-and-answer interactions, failing to capture the dynamic, interactive information needs of real-world clinician-patient conversations. Although blinding and standardized assessment tools were employed, the limited number of evaluators may still introduce subjective bias. Second, this study evaluated the quality, reliability, and readability of the model-generated responses using validated instruments such as DISCERN, EQIP, and GQS. These tools, however, are designed to assess structural completeness, comprehensiveness, and perceived information quality, and should not be construed as surrogates for clinical factual accuracy. A response may attain a high DISCERN score due to its clear structure and balanced presentation, yet it still contains subtle factual errors or omissions that would only be detected through expert clinical benchmarking against current evidence. Therefore, while our findings reflect the overall quality of information as perceived by clinical reviewers, they do not guarantee the complete clinical correctness of every statement generated by the models. Future investigations incorporating systematic fact-checking against established clinical guidelines and comprehensive evidence syntheses will be essential to address this gap. Third, our assessment was conducted from the perspective of clinical expert evaluators and did not involve actual patients or end users. Consequently, our findings reflect the “potential utility” of these materials rather than their “actual effectiveness” in real-world settings. We did not directly measure patient-centered outcomes, such as comprehensibility, perceived usefulness, emotional impact, information retention, decision satisfaction, or effects on treatment adherence and health behaviors. It is widely acknowledged that even high-scoring educational content may fail to engage or be understood if it does not align with the health literacy levels, cultural backgrounds, or personal values of the target audience. Thus, while our study offers a foundational assessment of material quality, future research incorporating patient-reported outcome measures and usability testing across diverse patient populations will be critical for determining whether AI-generated materials can genuinely enhance patient education and SDM in clinical practice. Fourth, extensive subgroup analyses and statistical comparisons were conducted across four question domains and multiple evaluation tools. Although these analyses were prespecified and guided by clinical reasoning regarding the distinct nature of the four question categories, we acknowledge that the multiplicity of comparisons may have increased the risk of Type I errors (false-positive findings). Readers are therefore advised to interpret the subgroup results as hypothesis-generating rather than confirmatory in nature. Future confirmatory studies with larger question banks and preregistered analytical plans will be warranted to validate the domain-specific performance patterns observed in the current study. Fifth, with regard to content, the study focused exclusively on the specific area of endometrial cancer surgery, and the conclusions may not be generalizable to other gynecological cancer treatment modalities (such as chemotherapy and radiotherapy) or cancer types. Third, as technological development is highly time-sensitive, the results based on specific model versions (ChatGPT-5 or DeepSeek-R1) and query timing may quickly become outdated due to rapid model iteration. Finally, although the evaluation dimensions were relatively comprehensive, they are process indicators and do not directly assess the long-term impact of the generated materials on patient knowledge acquisition, decision satisfaction, anxiety levels, or clinical outcomes.

This study assesses readability based on English-language input and output. The readability formulas employed, including FKGL, FRES, and GFI, are constructed upon linguistic features specific to English texts (such as syllable count, word length, and sentence length), and their scoring criteria are directly benchmarked against the U.S. educational system. Consequently, the conclusion made in this study that “the AI-generated texts present a reading difficulty exceeding the general public's health literacy level” is explicitly language- and culture-specific. Its direct implications should therefore be strictly confined to older adult patients with endometrial cancer in English-speaking, high-income countries and regions.

For non-native English-speaking populations—including patients in China and other non-English-speaking countries—even though the reading difficulty of AI-generated materials is lowered to the 6th−8th grade level recommended by the American Medical Association (AMA), these materials may still face considerable barriers in clinical translation. The reason lies in the fact that medical English itself constitutes a “professional language barrier”: non-native speakers must not only negotiate the cognitive demands of “medical expertise” but also shoulder the added burden of “processing information in a non-native language.” Therefore, for AI-generated patient education materials to achieve global empowerment, they must transcend “linguistic simplicity” and strive for “cognitive accessibility.” Given the considerable heterogeneity in health education systems across countries and regions, cultural traditions, and patient participation models in medical decision-making, there is an urgent need to develop and validate cross-lingual, cross-cultural readability assessment systems in future research, rather than simply applying standardized thresholds from a single country.

Furthermore, this study was conceived as a “benchmarking assessment” of the foundational educational capabilities of two mainstream LLMs. To this end, a standardized question-and-answer text format was necessarily adopted to minimize the influence of confounding variables. However, in real-world perioperative patient education settings, the effectiveness of information delivery depends on far more than the mere presentation of written content. Visual materials (e.g., surgical anatomy diagrams), short videos (e.g., post-operative rehabilitation exercise demonstrations), and context-sensitive, multi-turn interactive dialogues (allowing patients to pose follow-up questions and clarify misunderstandings) are widely recognized as important contributors to patient comprehension and engagement. In this context, current plain-text LLM outputs, which lack multimodal plugin support, do not yet appear poised to directly supplant the existing, visually optimized patient education booklets.

It is worth emphasizing that the DISCERN, EQIP, GQS, and readability formulas employed in the present work are all surrogate evaluation indicators. They reflect whether the materials are “well-written” and “clearly structured,” measuring the intrinsic structural quality of the information materials rather than their actual educational effectiveness for patients in real clinical settings. Although AI-generated texts receive high scores on these metrics, their actual clinical utility may be considerably diminished if their presentation format fails to align with the patient's cognitive load, cultural background, or emotional state—particularly the preoperative anxiety levels commonly experienced by cancer patients. To this end, a three-phase, sequential approach is proposed for future research. Phase 1—Knowledge Acquisition and Retention Validation: a prospective randomized controlled trial should be designed to directly compare AI-generated materials with traditional educational booklets. Standardized questionnaires (e.g., objective knowledge tests) should be used to quantitatively assess short- and long-term knowledge retention rates following patient reading or interaction. Phase 2—Decision Quality and Psychological Impact Assessment: validated instruments including the Decisional Conflict Scale (DCS) and the State-Trait Anxiety Inventory (STAI) should be incorporated to dynamically monitor improvements in patients' decisional clarity, trust in the medical team, and perioperative anxiety levels after exposure to AI-assisted materials. Phase 3—Real-World, Multi-Center Clinical Utility Validation: a multi-center observational study should be designed to encompass hospitals across different regions and tiers (tertiary A-level vs. primary-level hospitals) and diverse economic and cultural settings. AI-generated materials should be seamlessly integrated into routine clinical workflows (e.g., pre-admission education systems). Besides, their implementation effectiveness, feasibility, and acceptance should be evaluated by healthcare providers through objective metrics including actual patient click-through rates, reading completion times, and subsequent rates of unscheduled consultations (e.g., repeated phone inquiries).

## 5 Conclusion

In summary, both DeepSeek and ChatGPT are the promising sources of patient education materials for endometrial cancer surgery. When applying LLMs to specialized patient education, it is vital to avoid simplistic judgments of one model being “better” than the other and instead recognize that their performance exhibits significant domain specificity. DeepSeek demonstrates higher reliability in handling structured, procedural clinical management knowledge (e.g., postoperative care and long-term follow-up), whereas ChatGPT offers a slight readability advantage when addressing decisional knowledge that requires trade-offs and explanations (e.g., surgical planning). These findings suggest that in future clinical practice, different AI tools can be flexibly selected and combined based on specific task requirements, rather than relying on a single model to address all educational needs. However, given that this study did not perform systematic fact-checking against clinical guidelines as the gold standard, the clinical factual accuracy of these materials requires further validation prior to their application in practice.

## Data Availability

The raw data supporting the conclusions of this article will be made available by the authors, without undue reservation.
