# Supplementary material for: Evaluating AI-generated patient education materials for endometrial cancer surgery: a comparative analysis of response quality, reliability, and readability between ChatGPT and DeepSeek models
Source: Front Public Health. 2026 Aug 26;14:1831988. doi: 10.3389/fpubh.2026.1831988 (PMC13553861; doi:10.3389/fpubh.2026.1831988)
Supplement: Supplementary file 1 [file Table_1.docx]

Supplementary Table 1: Top 25 Google search terms related to endometrial cancer (obtained via Google Trends), including the relative search popularity ranking for each of the 25 terms.

| Category: All categories |  |  |
| --- | --- | --- |
| Endometrial Cancer: (1/1/04 - 12/12/25, Worldwide) |  |  |
|  |  |  |
|  | TOP |  |
| 1 | symptoms endometrial cancer | 100 |
| 2 | cancer symptoms | 100 |
| 3 | uterine cancer | 69 |
| 4 | what is endometrial cancer | 55 |
| 5 | endometrial cancer stage | 52 |
| 6 | ovarian cancer | 50 |
| 7 | endometriosis cancer | 46 |
| 8 | endometrial cancer treatment | 45 |
| 9 | endometriosis | 44 |
| 10 | symptoms of endometrial cancer | 40 |
| 11 | cervical cancer | 39 |
| 12 | uterus cancer | 32 |
| 13 | uterus | 32 |
| 14 | staging endometrial cancer | 31 |
| 15 | endometrial cancer icd 10 | 31 |
| 16 | hyperplasia | 27 |
| 17 | endometrial hyperplasia | 27 |
| 18 | vaginal cancer | 27 |
| 19 | endometrial biopsy | 26 |
| 20 | endometrial carcinoma | 25 |
| 21 | endometrium | 24 |
| 22 | hysterectomy | 24 |
| 23 | breast cancer | 24 |
| 24 | endometrium cancer | 24 |
| 25 | endometrial cancer causes | 24 |
|  | Category: All categories |  |
|  | Endometrial Cancer: (1/1/04 - 12/12/25, Worldwide) |  |

**Supplementary Table 2: Questions on Endometrial Cancer-Related Educational Materials.**

Question

**I. Surgical Planning and Technical Considerations**

Do I have to have my uterus removed? Are there any treatment options that preserve the uterus?

Will the surgery be minimally invasive (laparoscopic/robotic) or open? How should I choose? What are the advantages and disadvantages of each?

Why are the ovaries and fallopian tubes often removed during surgery? I am still young—can I keep my ovaries?

What is "sentinel lymph node biopsy"? How does it differ from traditional "systematic lymph node dissection"? What are the benefits for me?

Will a "frozen section" pathology examination be performed during surgery? What is its purpose?

What exactly does "comprehensive staging surgery" include? Why are these steps necessary?

If I am an early-stage patient, is "fertility-sparing treatment" possible? What conditions must be met? How high are the risks?

Is robotic surgery better than traditional laparoscopy? Is it worth the significantly higher cost?

How long will the surgery take? Are the risks of general anesthesia high?

How is the completeness of the surgery assessed? How can it be ensured that the tumor is fully removed?

**II. Preoperative Evaluation and Optimization**

What tests do I need to complete before surgery? (e.g., MRI, CT, CA125, cardiopulmonary function assessment, etc.) What is the purpose of these tests?

I have chronic conditions like hypertension/diabetes. How should they be optimized before surgery?

My doctor recommends "neoadjuvant therapy" (such as chemotherapy or radiation) before surgery. Why is this?

Do I need "genetic counseling and testing" (e.g., for Lynch syndrome)? What does this mean for me and my family?

What should I pay attention to in terms of diet and nutrition before surgery? Do I need a blood transfusion or white blood cell boosters in advance?

I am taking aspirin/anticoagulants/supplements. How long before surgery should I stop them?

How can postoperative thrombosis (deep vein thrombosis) be prevented? What preparations are needed before surgery?

My anemia is severe. Will it affect the surgery? Does it need to be corrected first?

Is bowel preparation required before surgery? How is it done?

What psychological and practical preparations should I make before surgery?

**III. Postoperative Care and Complication Management**

How is postoperative pain managed? How long can a patient-controlled analgesia pump be used?

How long will the urinary catheter and drainage tubes remain? Is their removal painful?

How soon after surgery can I get out of bed, eat, and drink? What dietary restrictions and recommendations are there?

How should the wound be cared for? When can I shower? Do the minimally invasive puncture sites require special care?

What are the dangerous "warning signs" after surgery? (e.g., fever, redness/discharge from the wound, leg swelling, difficulty breathing, abnormal abdominal pain, etc.)

What are the common postoperative complications (e.g., lymphocyst, urinary tract infection, intestinal obstruction, etc.)? How can they be prevented and managed?

How long will I likely need to stay in the hospital? What are the criteria for discharge?

How soon after discharge can I resume normal life and work? When can I exercise (e.g., walk, swim) and resume sexual activity?

How soon after discharge can I resume normal life and work? When can I exercise (e.g., walk, swim) and resume sexual activity?

What should I do if I experience menopausal symptoms (hot flashes, insomnia, mood swings) after surgery? Is hormone replacement therapy an option?

How does the vaginal cuff heal? Will there be bleeding or discharge after surgery? What is considered normal?

**IV. Long-Term Follow-Up and Disease Management**

When will my final pathology report be available? What do terms like "stage," "grade," "depth of myometrial invasion," and "lymphovascular space invasion" mean on the report?

Based on my pathology report, is my recurrence risk high or low? What factors affect my prognosis?

Will I need postoperative adjuvant therapy (radiation, chemotherapy, targeted/immunotherapy)? Why or why not?

What is the long-term follow-up plan? (e.g., How often should I have check-ups? What tests are done each time: gynecological exams, tumor markers, imaging?)

If tumor markers (e.g., CA125) rise during follow-up, does it always mean recurrence? What should I do?

How can potential long-term complications be monitored and managed? (e.g., lymphedema, increased cardiovascular disease risk, osteoporosis, etc.)

To prevent recurrence, what specific lifestyle changes (diet, exercise, weight management) should I make?

Can I receive vaccines such as the HPV vaccine or COVID-19 vaccine?

Will my disease and treatment have long-term effects on my bone health (osteoporosis), cardiovascular health, and cognitive function? How can these be addressed?

If recurrence or metastasis occurs in the future, what effective treatment options remain? Will current treatments affect future options?

Table 3 Global Quality Scale (GQS) criteria

| Score | Description |
| --- | --- |
| 1 | Poor quality and flow, most information missing, not at all useful for patients |
| 2 | Generally poor quality and flow, some information listed but many important topics missing, of very limited use to patients |
| 3 | Moderate quality, suboptimal flow, some important information is adequately discussed but others poorly discussed, somewhat useful for patients |
| 4 | Good quality and generally good flow, most of the relevant information is listed, but some topics are not covered, useful for patients |
| 5 | Excellent quality and excellent flow, very useful for patients |

**DISCERN score**

The DISCERN instrument consists of a checklist containing 16 questions. These questions are divided into three main sections:

**Section 1: Reliability of the Content (Questions 1–8)**

This section focuses on whether the information itself is trustworthy and unbiased.

*	Are the aims clear? Does the information clearly state its purpose?

*	Does it achieve its aims?

*	Is it relevant? Is the information relevant to the target audience?

*	Is it clear what sources of information were used? Are references or data sources cited?

*	Is it clear when the information was produced? Is the information up-to-date? Is there a clear publication or revision date?

*	Is it balanced and unbiased? Are different treatment options presented fairly, without inappropriate bias toward one?

*	Does it provide details of additional sources of support and information?

*	Does it refer to areas of uncertainty? Are any potential conflicts of interest disclosed (e.g., funding from a pharmaceutical company)?

**Section 2: Specific Details about Treatment Choices (Questions 9–15)**

This section specifically assesses the quality of information about treatment options, with particular emphasis on enabling the reader to make an informed choice.

*	9. Does it describe how each treatment works?

*	10. Does it describe the benefits of each treatment?

*	11. Does it describe the risks of each treatment?

*	12. Does it describe what would happen if no treatment is used?

*	13. Does it describe how the treatment choices affect overall quality of life?

*	14. Is it clear that there may be more than one possible treatment choice?

*	15. Does it provide support for shared decision-making? Does the information help patients make supportive decisions based on their own values and preferences?

**Section 3: Overall Rating (Question 16)**

*	16. Based on the answers to all of the above questions, rate the overall quality of the publication as a source of information about treatment choices.

**How to Use the DISCERN Score?**

Each question is scored on a scale of **1–5 points**:

*	1 point: No, the criterion is not met at all (e.g., risks are not mentioned at all).

*	2 points: Partially, the criterion is met but with major shortcomings.

*	3 points: Partially, the criterion is met with minor shortcomings.

*	4 points: Yes, the criterion is met with minor reservations.

*	5 points: Yes, the criterion is fully met.

**Interpreting the Total Score:**

*	**High score (>55 points):** Indicates high-quality information and a reliable source.

*	**Moderate score (39–54 points):** Information quality is acceptable but may have some important omissions or shortcomings; use with caution.

*	**Low score (<39 points):** Information quality is poor, with serious flaws; it may be misleading and is not recommended as a basis for decision-making.

**Main Contents of EQIP**

The EQIP checklist contains 20 items, divided into four main domains. Each item is scored as "Yes" (1 point), "Partially" (0.5 points), or "No" (0 points). The total score is expressed as a percentage (maximum 100%).

**Part 1: Content Scope and Quality**

This section focuses on whether the information is accurate, relevant, and comprehensive.

1. Purpose: Is the purpose of the information (e.g., for education, guiding decisions) clearly stated?

2. Background: Is background information on the health condition provided (e.g., definition, causes, prevalence)?

3. Relevance: Is the information relevant to the target audience (e.g., patients, families)?

4. Key messages: Are the key messages or recommendations highlighted in the text?

5. Practicality: Is the information practical? Can patients act on it?

6. Accuracy: Is the information medically accurate? (This may require expert knowledge to judge.)

7. Sources: Are the sources of information provided (e.g., clinical guidelines, research)?

8. Date: Is a publication date or "last updated" date included?

**Part 2: Structure and Design**

This section evaluates the formatting, layout, and readability of the information, which are crucial for user experience.

9. Headings: Are headings and subheadings used to organize content?

10. Logical flow: Is the information presented in a logical sequence?

11. Font and typography: Is the font size and style easy to read?

12. White space: Is there sufficient white space to avoid a cluttered look?

13. Emphasis: Are bold, italics, or bullet points used to highlight key information?

14. Color: Is color used to enhance readability (rather than distract)?

**Part 3: Identification and Patient-Centered Factors**

This section focuses on the transparency of the information and whether it considers patients' individual needs.

15. Authorship/Source: Are the author and/or publishing organization clearly identified?

16. Conflicts of interest: Are any potential conflicts of interest declared?

17. Patient perspective: Does the information reflect patient viewpoints and experiences? (e.g., uses patient quotes)

18. Different options: Where relevant, are different treatment or management options mentioned?

**Part 4: Evaluation and Understanding**

This section assesses whether the information genuinely helps patients understand and make decisions.

19. Summary: Is a summary or recap of key points provided?

20. Understanding check: Does the information include elements that encourage readers to check their understanding? (e.g., "Ask yourself these three questions..." or a short quiz)

****How to Use EQIP?****

1. Select Material: Identify the health information you want to evaluate (e.g., a PDF booklet, a webpage).

2. Review Item by Item: Read the material carefully against the 20-item checklist.

3. Score: Assign a score of "Yes" (1 point), "Partially" (0.5 points), or "No" (0 points) to each item.

4. Calculate Total Score: Add the scores for all items, divide by 20 (the total possible score), and multiply by 100% to get a percentage score.

*	Formula: (Total Score / 20) * 100% = EQIP Total Score (%)

5. Interpret the Score:

*	**High score (e.g., >75%):** Indicates high quality in content, design, transparency, and usability.

*	**Moderate score (e.g., 50%–75%):** Information quality is acceptable but has several areas needing improvement.

*	**Low score (<50%):** Information quality is poor; it may be misleading, difficult to use, or lacking key details.
