# Supplementary material for: Evaluating AI-generated patient education materials for endometrial cancer surgery: a comparative analysis of response quality, reliability, and readability between ChatGPT and DeepSeek models
Source: Front Public Health. 2026 Aug 26;14:1831988. doi: 10.3389/fpubh.2026.1831988 (PMC13553861; doi:10.3389/fpubh.2026.1831988)
Supplement: Supplementary file 2 [file Table_2.docx]

|  | **chatGPT** | **DeepSeek** |
| --- | --- | --- |
| **I. Surgical Planning and Technical Considerations** |  |  |
| Do I have to have my uterus removed? Are there any treatment options that preserve the uterus? | Short answer first, no sugarcoating: most people with endometrial cancer do have their uterus removed, because surgery is the most reliable way to cure it. But uterus-preserving options do exist in specific, limited situations. This isn’t folklore or wishful thinking—it’s a real, evidence-based fork in the road.  Here’s the lay of the land.  For the majority of cases, the standard treatment is a hysterectomy (removal of the uterus), often with removal of the ovaries and fallopian tubes. Why so aggressive? Endometrial cancer usually starts in the uterine lining and can quietly invade deeper layers or spread. Surgery removes both the tumor and its staging uncertainty in one decisive move. From a cancer-control perspective, it’s hard to beat.  Now the exception—the one people care about deeply.  Uterus-preserving (fertility-sparing) treatment may be considered if all of the following are true: • The cancer is very early stage • It is low-grade (grade 1) endometrioid adenocarcinoma • The disease is confined to the endometrium (no invasion into the muscle on MRI) • There is no spread outside the uterus • You understand and accept higher recurrence risk and close surveillance  In that narrow window, treatment usually involves high-dose progestin therapy (hormonal treatment). This can be: – Oral progestins (like medroxyprogesterone or megestrol) – A levonorgestrel-releasing IUD – Sometimes a combination of both  This approach is not casual. It requires: • Frequent endometrial biopsies • Regular imaging • A clear plan that hysterectomy will still be done later, often after childbearing or if the cancer doesn’t fully respond. Success rates are real but imperfect. Many patients do achieve remission; a significant fraction will recur. This is management, not eradication.  What does not work well as uterus-sparing treatment: – Radiation alone (it controls disease but destroys fertility and uterine function) – “Watchful waiting” – Alternative therapies – Half-measures without close oncology supervision  The uncomfortable truth: uterus preservation trades certainty for possibility. It’s a reasonable trade only when the biology of the cancer is gentle and the monitoring is strict.  One more important note: if the cancer is higher grade, has muscle invasion, or is more advanced, uterus-preserving treatment is generally unsafe. At that point, delaying surgery meaningfully increases risk.  So the real question is not “Do I have to remove my uterus?” but rather “Does my specific cancer biology allow me to safely delay or avoid hysterectomy?”  That answer lives in pathology reports, MRI findings, and oncology guidelines—not optimism or fear. | This is an excellent and very important question. The short answer is: For most people with endometrial cancer, removal of the uterus (hysterectomy) is the standard and most effective treatment. However, in a specific, select group of patients, there are uterus-preserving options available.  Let's break this down clearly.   The Standard Treatment: Hysterectomy For the majority of endometrial cancer cases, a total hysterectomy (removal of the uterus and cervix) along with removal of the fallopian tubes and often the ovaries is the primary treatment. This is recommended because: It is curative for most early-stage cancers. It allows surgeons to stage the cancer accurately (check if it has spread), which guides further treatment. It eliminates the primary source of the cancer, providing the best chance for a cure.  So, for most patients, especially those who have completed childbearing, a hysterectomy is the recommended course.     Uterus-Preserving (Fertility-Sparing) Treatment Options These options are NOT for everyone. They are considered only under very strict criteria and involve significant risks. The goal is to allow for future pregnancy.  Candidate Criteria (all must be met): Younger age (typically under 40-45 and strongly desiring future fertility). Early-Stage, Low-Grade Tumor: Grade 1 endometrioid adenocarcinoma (the most common type) that is well-differentiated. Confined to the Uterus: Imaging (MRI) must show the cancer is Stage IA, with no invasion into the deep muscle of the uterus and no spread outside the uterus. No Suspicion of Ovarian Cancer: The ovaries should appear normal. No Genetic Syndromes: Such as Lynch syndrome, which raises the risk of other cancers. The patient must understand and accept the significant risks, including the risk of cancer progression during treatment.  The Primary Uterus-Preserving Treatment is: High-Dose Progestin Therapy How it works: Progestin hormones counteract estrogen and can cause cancer cells to shrink. It is given as a high-dose pill (like megestrol acetate) or through a levonorgestrel-releasing IUD (Mirena®). Process: This is not a one-time treatment. It requires:  1. Close Monitoring: You will need a D&C (dilation and curettage) to remove all the cancer tissue before starting hormones, and then repeat biopsies (usually every 3-6 months) to ensure the cancer is responding.  2. Timeline: Treatment continues until the cancer is gone, which can take 6-12 months or longer.  3. Pregnancy Planning: Once the cancer has completely regressed, you are encouraged to attempt pregnancy as soon as possible, often with the help of a fertility specialist.  4. Definitive Surgery Later: After completing childbearing, a hysterectomy is strongly recommended due to the high risk of the cancer coming back.   Crucial Steps You Must Take  1. Get a Precise Diagnosis: The single most important factor is your pathology report. You need to know the exact type, grade, and stage of your cancer. Ask your doctor for this report. 2. Consult a Specialist Team: This decision requires a Gynecologic Oncologist (cancer surgeon) and often a Reproductive Endocrinologist (fertility specialist). They will review your imaging (MRI) and pathology to see if you meet the strict criteria. 3. Have a Detailed Risk-Benefit Discussion: If you are a candidate, your doctors will explain the risks:  Risk of Progression: The cancer may not respond or may progress during hormone therapy, requiring immediate hysterectomy.  Risk of Recurrence: Even if it works, the risk of the cancer coming back in the uterus is significant (up to 30-40%).  Need for Rigorous Follow-up: You must commit to frequent biopsies and imaging.   Summary For most patients: Hysterectomy is the standard, curative treatment. For a select few: If you are young, have a very early, low-grade cancer, and desire future pregnancy, high-dose progestin therapy may be an option under the strict guidance of a specialist team. The decision is highly personal and medical: It depends entirely on your specific cancer characteristics, your age, your fertility desires, and your willingness to accept the risks.  Your next step is to schedule an appointment with a Gynecologic Oncologist. Bring a list of your questions, ask for a copy of your pathology report, and have a detailed discussion about all your options based on your unique situation.  Wishing you strength and clarity as you navigate this path. |
| Will the surgery be minimally invasive (laparoscopic/robotic) or open? How should I choose? What are the advantages and disadvantages of each? | Short, honest headline: for most endometrial cancer patients today, minimally invasive surgery is the default, not the exception. Open surgery still has a role, but it’s no longer the first tool you reach for unless there’s a good reason.  Let’s unpack this without romance or fear.  Minimally invasive surgery (laparoscopic or robotic) This includes traditional laparoscopy and robot-assisted laparoscopy. From a cancer-control standpoint, they are equivalent to open surgery in early-stage endometrial cancer. That’s not opinion—that’s data.  Why surgeons like it: • Much smaller incisions • Less blood loss • Less postoperative pain • Faster recovery (often home in 1–2 days) • Lower risk of wound infections and hernias • Earlier return to normal life  For patients with obesity, diabetes, or cardiovascular disease—common in endometrial cancer—minimally invasive surgery actually reduces complications, which was a big paradigm shift over the last 15 years.  Limitations: • Requires surgeon experience (this matters more than the robot itself) • Can be technically difficult in very advanced disease • Slightly longer operating time in some cases  Robotic vs standard laparoscopy: from your perspective, the difference is modest. Robotics can help surgeons in tight spaces or with complex anatomy, but surgeon skill outweighs platform choice.  Open surgery (laparotomy) This is the traditional large abdominal incision.  Why it’s still used: • Very large uterus or bulky tumors • Extensive disease requiring complex resections • Dense adhesions from prior surgeries • Situations where quick access and tactile feedback are critical  Advantages: • Direct visualization and manual control • Sometimes shorter operating time in very complex cases  Disadvantages: • Larger incision • More pain • Higher risk of infection and blood clots • Longer hospital stay (3–5 days is typical) • Slower return to normal activity  Now the key part—how you should choose.  You don’t choose based on fear, scar size, or the word “robot.” You choose based on:  1. Cancer stage and imaging 2. Your anatomy and medical conditions 3. Your surgeon’s experience and outcomes  A highly experienced surgeon doing laparoscopy beats an inexperienced surgeon doing robotics, and both beat a reluctant open approach done “just in case.”  Practical, no-nonsense questions to ask your surgeon: • How often do you perform minimally invasive hysterectomy for endometrial cancer? • What percentage of your cases are done minimally invasively? • What’s your conversion rate to open surgery? • Based on my MRI and biopsy, why are you recommending this approach?  A surgeon who answers clearly and comfortably is a good sign. Vagueness is not.  Bottom line: If your cancer is early stage and your surgeon is experienced, minimally invasive surgery is usually the best balance of safety, recovery, and cancer control. Open surgery is a tool, not a failure—but it should be chosen for a reason, not inertia.  The decision is less about technology and more about judgment. | This is another crucial question when planning for surgery. The choice between minimally invasive surgery (MIS) and open surgery (laparotomy) depends on multiple factors. Here’s a detailed breakdown to help you understand and discuss with your surgeon.   Current Standard for Early-Stage Disease: Minimally Invasive Surgery For the majority of endometrial cancer cases that appear to be early-stage, minimally invasive surgery is the preferred and recommended approach. This includes: Laparoscopic Surgery: The surgeon operates using long, thin instruments inserted through small incisions, guided by a camera. Robot-Assisted Laparoscopic Surgery: The surgeon controls robotic arms that hold the instruments, allowing for enhanced precision, dexterity, and 3D visualization.  Both laparoscopic and robotic surgeries are forms of MIS and share similar benefits.   Comparison: Advantages & Disadvantages  \| Feature \| Minimally Invasive (Lap/Robotic) \| Open Surgery (Laparotomy) \| \| : \| : \| : \| \| Incisions \| Several small incisions (0.5-1.2 cm) \| One large vertical or horizontal incision (10–15 cm) \| \| Hospital Stay \| Shorter. Typically 1–2 nights. \| Longer. Usually 3–4 nights or more. \| \| Blood Loss \| Less. \| More. \| \| Pain After Surgery \| Generally less. Pain is more from gas/air used and port sites. \| Generally more significant. Due to large incision and muscle retraction. \| \| Recovery Time\| Faster. Return to normal activities in 2-4 weeks. \| Slower. Return to normal activities in 6-8 weeks or more. \| \| Infection Risk \| Lower risk of wound infection. \| Higher risk of wound infection. \| \| Visualization \| Excellent, magnified view of pelvis/abdomen. \| Direct, wide view of the entire abdomen with surgeon's hands inside. \| \| Surgeon Dexterity \| Enhanced with robotics; standard with laparoscopy. \| Direct tactile feedback and unrestricted range of motion. \| \| Key Disadvantage\| Not suitable for all cases. May need to convert to open during surgery. In very rare historical studies (re: cervical cancer), concerns were raised about tumor disruption, but this is not supported for endometrial cancer in multiple large studies. \| Longer recovery, more pain, higher complication rates (infections, hernias, adhesions). \|   How Should You Choose? Key Decision Factors  The choice isn't yours alone; it's a collaborative decision with your Gynecologic Oncologist based on:  1. Cancer Characteristics & Stage:  Favorable for MIS: Early-stage cancer, small uterus, and no significant spread suspected.  May Require Open Surgery: Bulky disease, cancer that appears to have spread deeply into the uterine muscle or to other organs (advanced stage), very large uterus (fibroids), or involvement of the cervix. Open surgery provides the best exposure for complex cancer removal and comprehensive staging.  2. Your Body & Health History:  Favorable for MIS: Lower BMI. While MIS can be done on patients with higher BMI (and is often preferred to reduce wound complications), extreme obesity can make it technically challenging.  May Require Open Surgery: Multiple prior major abdominal surgeries creating dense scar tissue (adhesions), severe medical conditions that make lengthy anesthesia for MIS risky, or an inability to tolerate the Trendelenburg position (tilted head-down) required for MIS.  3. Surgeon Skill & Hospital Resources:  The surgeon's training, experience, and comfort level with complex MIS or robotic procedures are paramount. You should ask about their conversion rate (how often they need to switch to open surgery).   The Process: What to Expect & Key Questions to Ask 1. Preoperative Planning: Your surgeon will decide the planned approach based on your exam, biopsy, and imaging (MRI/CT). 2. Consent for Conversion: You will sign a consent form that includes permission for conversion from MIS to open surgery if needed. This is not a failure but a critical safety decision made if the surgeon encounters unexpected findings (such as widespread disease or adhesions) that make it unsafe to proceed minimally invasively. 3. Final Decision in the OR: Sometimes, the final decision is made after the surgeon places the camera and visually inspects the abdomen.  Questions to Ask Your Gynecologic Oncologist: "Based on my scans and exam, am I a candidate for minimally invasive surgery?" "What is your preferred approach (straight laparoscopy vs. robotic) for my case, and why?" "What is your personal and institutional rate of converting from MIS to open surgery?" "What factors in my *specific case might make an open surgery more likely?" "If we start with MIS and find more advanced disease, what is the plan?"   Summary: For standard early-stage endometrial cancer, minimally invasive surgery (laparoscopic or robotic) is the recommended first-line approach due to faster recovery, less pain, and fewer complications with equal cancer outcomes. Open surgery remains a vital, necessary tool for more advanced cancers, complex anatomy, or when patient safety demands it. The decision is made by your surgical team based on medical factors. Your role is to understand the rationale, ask questions, and prepare for the possibility of conversion.  The most important thing is to have a surgeon you trust who is experienced in both techniques and will choose the safest, most effective path for your unique situation. |
| Why are the ovaries and fallopian tubes often removed during surgery? I am still young—can I keep my ovaries? | Here’s the straight truth, without hand-waving: the ovaries and fallopian tubes are removed not out of habit, but out of risk management. That said, some young patients can safely keep their ovaries—but only when the biology cooperates.  Let’s break the logic, not just the rule.  Why surgeons usually remove ovaries and tubes (BSO) Three main reasons.  First, estrogen fuels many endometrial cancers. The ovaries are the body’s estrogen factory. Leaving them in place can—*in some cancers*—increase the risk of recurrence. This is less about today’s tumor and more about tomorrow’s microscopic leftovers.  Second, occult spread. Even when ovaries look normal, a small percentage of patients have microscopic cancer involvement. It’s uncommon, but cancer plays a long game, and surgery is about closing loopholes.  Third, the fallopian tubes. Modern pathology has made one thing very clear: many “ovarian” cancers actually start in the fallopian tubes. Removing the tubes reduces future cancer risk with almost no downside.  So removal is a defensive move: eliminate hormone stimulation, eliminate hidden disease, eliminate future risk.  Now the important part.  Can a young patient keep her ovaries? Sometimes, yes.  Ovarian preservation may be considered if *all of the following are true: • You are premenopausal and young • The cancer is low-grade (grade 1) • It is early stage and confined to the uterus • There is no ovarian involvement on imaging • No high-risk histology (not serous, clear cell, or high-grade) • You accept a small but real increase in recurrence risk  In these cases, studies show that keeping the ovaries does not significantly worsen overall survival and it avoids premature menopause.  That last point matters more than people realize.  What you gain by keeping your ovaries • Natural estrogen production • Avoidance of sudden menopause • Lower long-term risk of osteoporosis • Better cardiovascular health • Fewer severe menopausal symptoms • Better quality of life for many young patients  This is not cosmetic—it’s endocrine physics.  What you give up • A small increase in theoretical cancer risk • Less “absolute certainty” compared to removal • The option requires careful pathology and follow-up  This is a classic oncology tradeoff: maximum risk reduction vs long-term physiological cost.  One nuance people miss: Some surgeons will remove the fallopian tubes but preserve the ovaries (salpingectomy with ovarian preservation). This reduces future ovarian cancer risk while maintaining hormones. It’s a reasonable middle ground in select patients.  So the real answer is not “yes” or “no.” It’s: your age matters, but your tumor biology matters more than your birth certificate.  If your cancer is low-risk, ovarian preservation can be a rational, evidence-based choice—not reckless optimism. If your cancer is high-risk, keeping ovaries is usually a bad bet, no matter how young you are.  Cancer treatment is about probabilities, not promises. The art is choosing which risks you’re willing to live with long after the incision heals. | This is an absolutely critical question, especially for younger patients. The decision about whether to remove the ovaries (oophorectomy) and fallopian tubes (salpingectomy) during a hysterectomy for endometrial cancer involves weighing cancer risk against hormonal and long-term health benefits.   Why Are They Often Removed? (The Standard Rationale)  1. Eliminate a Potential Source of Cancer Spread: Endometrial cancer can microscopically spread to the ovaries or fallopian tubes, even if imaging looks normal. Removing them is part of a complete cancer staging and treatment. 2. Remove a Source of Estrogen: Most endometrial cancers are estrogen-sensitive. The ovaries produce estrogen, which could theoretically fuel the growth of any remaining cancer cells. Removing them eliminates this source. 3. Reduce Future Cancer Risk: There is a known link between endometrial cancer and a slightly increased risk of ovarian cancer, either concurrently (at the same time) or later in life. Removing the tubes and ovaries prevents this future risk. 4. Updated Understanding of Ovarian Cancer Origin: Recent research shows that many high-grade serous ovarian cancers (the most lethal type) actually start in the fallopian tubes. Removing the tubes is now a standard part of risk reduction.  Because of these reasons, removing the tubes and ovaries (bilateral salpingo-oophorectomy, or BSO) has been the standard recommendation for decades, especially for women who are postmenopausal or near menopause.     The Case for Ovarian Preservation in Younger Women  For women under 45, especially those who are premenopausal, the decision is much more nuanced. There is a growing movement to consider ovarian preservation in select cases, focusing on quality of life and long-term health.  Why Keeping Ovaries Might Be Considered: Avoid Surgical Menopause: Removing ovaries in a premenopausal woman causes immediate, severe menopause. This brings hot flashes, night sweats, vaginal dryness, and potential sexual dysfunction. Long-Term Health Risks: Premature loss of ovarian hormones is linked to significantly higher risks of:  Heart Disease  Osteoporosis (bone loss)  Cognitive Decline (dementia risk)  Mood Disorders Preserving Natural Hormones: Ovarian hormones are involved in more than just reproduction; they affect the brain, heart, bones, skin, and metabolism.   Can You Keep Your Ovaries? The Eligibility Criteria  Ovarian preservation is NOT for everyone. It is a calculated risk taken only under strict conditions. Your gynecologic oncologist will consider:  Favorable Factors for Preservation: Young Age: Typically under 45 (sometimes under 50), and definitely premenopausal. Low-Risk Cancer Features:  Stage I, Grade 1 endometrioid adenocarcinoma.  No deep myometrial invasion (cancer hasn't grown deeply into the uterine muscle wall).  No signs of spread to the ovaries, tubes, or lymph nodes during surgery (frozen section analysis). No High-Risk Genetics: You do NOT have a genetic syndrome like Lynch Syndrome (which dramatically increases ovarian cancer risk) or BRCA mutations.  Absolute Contraindications for Preservation: Cancer that has spread to the ovaries (obviously). High-grade tumors (Grade 3, serous, clear cell, carcinosarcoma). Suspicion of Lynch Syndrome (based on personal/family history or tumor testing). Advanced stage disease.   The Current Compromise & What You Should Discuss  1. Fallopian Tubes Should Go: There is near-universal agreement that the fallopian tubes should be removed in all cases (opportunistic salpingectomy). This gives significant ovarian cancer risk reduction with minimal hormonal impact.  2. Shared Decision-Making is Key: This is a classic example of a decision that requires in-depth discussion with your gynecologic oncologist. You must weigh:  The small, but real, risk of an occult ovarian metastasis or future ovarian cancer.  The significant and proven benefits of avoiding premature menopause and its long-term health consequences.  Questions to Ask Your Doctor: "Based on my final pathology and surgical findings, what is my personal risk of having or developing ovarian cancer if I keep my ovaries?" "Given my age and cancer characteristics, do I meet the criteria where ovarian preservation is a safe option to consider?" "Should I have genetic testing (for Lynch Syndrome) before surgery to help guide this decision?" "If we preserve my ovaries, what is the follow-up plan to monitor them?"   Summary: Standard Practice: Removal of tubes and ovaries to ensure complete cancer treatment and prevent future ovarian cancer. For Young, Low-Risk Patients: Ovarian preservation (keeping ovaries, removing tubes) is a viable and increasingly considered option to avoid the severe health consequences of premature surgical menopause. The Decision is Highly Individualized: It depends on your exact cancer type and stage, your age, menopausal status, genetic risk, and your personal values regarding risk tolerance vs. quality of life.  Bring this question directly to your preoperative consultation. A thorough discussion with your gynecologic oncologist, potentially involving a genetic counselor, is essential to make the best choice for your long-term health and well-being. |
| What is a "sentinel lymph node biopsy"? How does it differ from traditional "systematic lymph node dissection"? What are the benefits for me? | Think of the lymphatic system as a city’s drainage and checkpoint network. Cancer spreads by sneaking through those channels. The question surgeons face is not “Should we look at the lymph nodes?” but “How much disruption is necessary to learn what we need to know?”  That’s where sentinel lymph node biopsy (SLNB) comes in.  What it is: A sentinel lymph node is the *first lymph node (or small group of nodes) that drains lymph fluid from the uterus. If cancer cells travel, they almost always pass through these nodes first.  During surgery, the surgeon injects a tracer—usually a fluorescent dye like indocyanine green—into the cervix. The dye travels along lymphatic channels and lights up the sentinel nodes, which are then selectively removed and analyzed in great detail by pathology.  If those nodes are clean, the odds that other nodes are involved are extremely low.  Traditional systematic lymph node dissection This is the older, more brute-force approach. Surgeons remove many lymph nodes from the pelvis (and sometimes the para-aortic area) on both sides, regardless of whether they look suspicious.  It gives information, but at a cost.  How they differ: In plain terms, SLNB is targeted sampling with high-resolution pathology. Systematic dissection is wide clearance with lower-resolution sampling per node.  Both aim to stage the cancer accurately. One does it surgically minimalist; the other does it surgically maximalist.  Why SLNB is now preferred in many patients For early-stage, low- to intermediate-risk endometrial cancer, large studies show that SLNB: • Detects node involvement as reliably as full dissection • Actually finds *micrometastases better because each node is examined more intensely • Does not worsen survival outcomes  And here’s the part that matters to you as a human, not a statistic.  Benefits to you • Much lower risk of lymphedema (chronic leg swelling) • Less nerve and vessel injury • Shorter operative time • Faster recovery • Fewer long-term complications  Lymphedema deserves emphasis. It’s not rare with full dissection; it’s lifelong, and it quietly degrades the quality of life. Avoiding it is not a trivial win.  When full lymph node dissection may still be used • High-grade or aggressive tumor types • Grossly enlarged or suspicious nodes • Failed or incomplete sentinel mapping • Situations where staging uncertainty would change treatment decisions  In other words, when the biology looks dangerous, surgeons widen the net.  One subtle but important point: SLNB is not “doing less.” It’s doing smarter. The removed sentinel nodes undergo ultrastaging—serial sectioning and special staining—so tiny clusters of cancer cells are detected that would be missed in routine exams of dozens of nodes.  So the irony is this: fewer nodes removed, more information gained.  Bottom line: Sentinel lymph node biopsy gives you the staging information your oncologist needs while sparing you many of the long-term complications of older techniques. For appropriately selected patients, it’s one of the clearest examples in modern cancer surgery where precision beat brute force.  Cancer surgery has matured. The goal is no longer maximal removal—it’s maximal insight with minimal collateral damage. | Excellent question. Understanding lymph node assessment is crucial in endometrial cancer surgery, as it directly impacts staging, prognosis, and treatment decisions. The shift from traditional dissection to Sentinel Lymph Node Biopsy (SLNB) is one of the most significant advances in surgical management. Here’s a detailed breakdown.   What is a Sentinel Lymph Node (SLN)? Imagine the lymphatic system as a network of highways draining fluid from an organ. The sentinel lymph node(s) are the first major "checkpoint" or "gateway" node(s) that cancer cells are most likely to travel to if they start to spread from the uterus.   The Two Procedures: A Side-by-Side Comparison  \| Feature \| Systematic (Complete) Lymph Node Dissection (LND) \| Sentinel Lymph Node Biopsy (SLNB) \| \| : \| : \| : \| \| What is Removed? \| A predetermined area containing many lymph nodes (e.g., all pelvic and sometimes para-aortic nodes). \| Only the 1–4 specific "sentinel" nodes identified by the mapping technique. \| \| Goal \| Maximum removal to find any possible cancer spread and reduce recurrence risk in that area. \| Targeted, intelligent sampling to find the nodes most likely to harbor cancer, if it has spread. \| \| Technique \| Anatomic landmark-based removal. \| Mapping: A blue dye and/or a fluorescent tracer with a radioactive component is injected into the cervix (or uterus). These tracers travel the same lymphatic pathways cancer cells would. The surgeon uses visual cues and a special camera to find and remove the nodes that "light up" or turn blue. \| \| Typical Number of Nodes \| Many (often 10–40+). \| Few (typically 1–4). \| \| Surgical Scope \| More extensive dissection around major blood vessels and nerves. \| Highly focused, minimally invasive removal of specific nodes. \|     How SLNB Works in Practice (The Process) 1. Injection: At the start of surgery, the surgeon injects the tracers into the cervix. 2. Mapping: Over 15–20 minutes, the tracers flow through the lymphatic channels. 3. Identification: Using a near-infrared camera (for fluorescence) and direct visualization (for blue dye), the surgeon pinpoints the glowing or blue-stained sentinel nodes in the pelvic areas. 4. Removal & Ultrastaging: The sentinel nodes are removed and sent to pathology for ultrastaging. This is a much more detailed examination than for nodes from a standard LND. Pathologists slice the node very thinly and use special stains to look for even the tiniest clusters of cancer cells (micrometastases).   What Are the Benefits for You? The adoption of SLNB represents a move toward personalized, precision surgery with fewer side effects, without compromising cancer care. Benefits include:  1. Drastic Reduction in Surgical Complications:  Lymphedema Risk: This is the biggest benefit. Lymphedema (chronic, painful swelling of the legs) is a lifelong risk after a full LND, occurring in 10–25% of patients. With SLNB, the risk is reduced to 1-3% because far fewer lymph channels are disrupted.  Lower Risk of Nerve Injury and Lymphoceles: Less dissection around pelvic nerves and vessels means lower risks of nerve damage (causing numbness/weakness) and symptomatic lymphoceles (fluid collections).  2. More Accurate Staging (Possibly Even Better):  Ultrastaging: Because pathologists focus intensely on just a few nodes, they can find micrometastases and isolated tumor cells that would be missed in the routine processing of dozens of nodes from an LND.  This can lead to more precise staging, which may identify a subset of patients who benefit from additional (adjuvant) treatment.  3. Ideal for Minimally Invasive Surgery:  SLNB integrates perfectly with laparoscopic and robotic surgery, allowing for precise node removal through small incisions.  4. Personalized Treatment Guidance:  If the SLN is negative, it is highly predictive that all other nodes are also negative. This spares you the morbidity of a full LND without compromising the accuracy of your staging.  If the SLN is positive, Your cancer stage is upgraded (to Stage IIIC), and this clearly indicates a need for adjuvant therapy (such as radiation or chemotherapy). The surgeon may then proceed to remove more nodes in that specific basin.   Important Considerations & When a Full LND Might Still Be Needed Not Finding the Sentinel Node: In a small percentage of cases (5–10%), a sentinel node cannot be identified on one or both sides. The surgeon will then perform a targeted lymphadenectomy on that side. High-Risk Cancers: For cancers with very high-risk features (e.g.,Extensive invasion, serous/clear cell types, grossly enlarged nodes), your surgeon may recommend proceeding directly to a therapeutic LND in addition to or instead of SLNB. Surgeon/Institution Experience: SLNB requires specific training, technology (near-infrared cameras), and a coordinated pathology protocol. It should be performed by a team experienced in the technique.   Summary & Your Next Steps Sentinel Lymph Node Biopsy is now the standard of care for surgical staging in early-stage endometrial cancer. It is a smarter, more targeted approach that reduces long-term side effects while providing equal or superior diagnostic information compared to removing many nodes indiscriminately.  Key Questions to Ask Your Gynecologic Oncologist: "Do you recommend a sentinel lymph node biopsy for my case, based on my pre-op imaging and biopsy?" "Is your team experienced in performing SLNB with both dye and fluorescence mapping?" "What is your protocol if a sentinel node is not found on one side?" "Does your pathology department perform ultrasound staging on sentinel nodes?"  This technique exemplifies modern cancer surgery: maximizing information and cure rates while minimizing the long-term physical burden on the patient. It’s a very positive development in the field, and you are right to be asking about it. |
| Will a "frozen section" pathology examination be performed during surgery? What is its purpose? | Yes—a frozen section *may be done during surgery, but it is selective, not automatic, and its value depends on context. It’s a tool, not a ritual.  Here’s what it actually is, and why surgeons sometimes use it.  What a frozen section is During surgery, a piece of tissue (usually the uterus after removal, sometimes a lymph node) is rushed to pathology. It’s rapidly frozen, sliced, stained, and examined under a microscope while you’re still asleep on the table. The pathologist then calls the operating room with a preliminary answer.  This whole process takes about 15–30 minutes.  What question the Frozen section is trying to answer is used to make *real-time decisions*. In endometrial cancer, the surgeon is usually asking one or more of these:  • How deep does the tumor invade the uterine muscle (myometrium)? • What is the tumor grade—does it look low-risk or high-risk? • Based on this, do we need to assess lymph nodes more aggressively?  In other words, it helps decide how far to go *during the same operation*.  How it affects what happens next: If the frozen section shows: – Superficial invasion + low grade → the surgeon may limit lymph node surgery – Deep invasion or high-risk features → the surgeon may proceed with sentinel node biopsy or full lymph node assessment  So frozen section can change the surgical plan *on the fly*.  But here’s the crucial honesty: the frozen section is not perfect.  Limitations: • Tumor grade can be underestimated • Microscopic invasion can be missed • It’s less reliable for certain aggressive subtypes • Final pathology (done days later) is always more accurate  Because of this, many modern centers rely more on: – Preoperative biopsy – MRI findings – Sentinel lymph node mapping  In those settings, frozen section may add little and is sometimes skipped.  When the frozen section is most useful: • Preoperative information is incomplete or conflicting • Imaging is unavailable or unclear • Surgical decisions hinge on the depth of invasion • The institution has experienced gynecologic pathologists  When it may not be necessary: • Clear pre-op diagnosis of low-risk disease • Planned sentinel lymph node biopsy regardless • Centers following strict SLNB algorithms • Situations where frozen results wouldn’t change management  Important nuance: doing a frozen section does not make surgery “better” by default. Doing an unnecessary one just adds time and cost without improving outcomes.  Bottom line: Frozen section pathology is a real-time decision aid, not a definitive diagnosis. Its purpose is to guide surgical extent while you’re still under anesthesia. Whether it’s used depends on how confident your team already is about your cancer’s behavior before surgery.  Modern endometrial cancer surgery is less about reacting mid-operation and more about planning intelligently ahead of time. Frozen section remains useful—but only when it meaningfully changes decisions. | Excellent and very astute question. The use of "frozen section" pathology during surgery is a critical, real-time decision-making tool, especially in endometrial cancer. It is a standard component of well-conducted oncologic surgery.  Here’s a detailed breakdown of what it is, why it’s done, and how it directly impacts your care.   What is a Frozen Section? A frozen section is a rapid intraoperative (during surgery) pathological examination of tissue. Here's the process: 1. Immediate Sampling: The surgeon removes your uterus and sends it directly from the operating room to the pathology lab. 2. Quick Freeze & Slice: The pathologist takes targeted slices of the tumor, rapidly freezes them to make them solid, and cuts them into very thin slices with a special microtome. 3. Stain & Examine: The slices are stained and placed under a microscope for immediate analysis. 4. Phone Call to OR: Within 20–40 minutes, the pathologist calls the surgeon in the operating room with the preliminary findings.   What is its purpose? (Why It's So Important) The primary purpose is to provide real-time information to guide the surgical plan while you are still under anesthesia. For endometrial cancer, the key questions it answers are:  1. Tumor Grade and Cell Type: Confirms the grade (aggressiveness) and specific type (e.g., endometrioid vs. aggressive serous/clear cell). Sometimes, the biopsy obtained before surgery can be upgraded or show a more serious type. 2. Depth of Myometrial Invasion: This is the most crucial factor. The pathologist measures how deeply the cancer has grown into the muscular wall of the uterus (the myometrium).  No or superficial invasion (<50%): Suggests low-risk disease.  Deep invasion (≥50%): Significantly increases the risk that cancer cells may have spread to lymph nodes or beyond.   How Does This Information Directly Impact Your Surgery? Based on the frozen section results, your surgeon makes critical decisions on the spot:  Lymph Node Assessment:  Low-Risk Findings (Grade 1–2, <50% invasion): The surgeon may proceed with a Sentinel Lymph Node Biopsy (SLNB) alone, as the risk of spread is low.  High-Risk Findings (Grade 3, serous/clear cell type, or ≥50% invasion): The surgeon may perform a more comprehensive lymph node dissection (or a more extensive SLNB mapping) to thoroughly check for spread, as the risk is much higher.  Decision on Ovarian Preservation (if previously planned):  If the frozen section reveals a high-grade tumor or an unexpected aggressive cell type, the surgeon will likely recommend removing the ovaries and fallopian tubes even if you are young, due to the higher risk of microscopic spread.  Extent of Surgery (Staging Procedure):  For very high-risk features, the surgeon might extend the surgery to include biopsies of the omentum (an abdominal fat pad) or perform peritoneal washings to check for microscopic abdominal spread.   Important Limitations to Understand Preliminary Diagnosis: A frozen section is not the final diagnosis. It is a highly accurate but rapid assessment. The final pathology report, which takes several days, involves a more thorough examination of the entire uterus with permanent (not frozen) sections. Rarely (in <5% of cases), the final diagnosis may change (e.g., a higher grade may be confirmed). Not a Guarantee: It cannot detect all microscopic features. Its main strength is assessing the key prognostic factors (grade and depth) quickly. Team-dependent: It requires skilled gynecologic pathologists and good communication with the surgical team.   Summary & Your Next Steps A frozen section is a standard, vital part of surgical staging for endometrial cancer. It transforms surgery from a one-size-fits-all procedure into a personalized, dynamic operation tailored to your cancer's specific behavior.  It allows your surgeon to make the most informed decisions possible while you are on the operating table. potentially avoiding unnecessary extensive surgery for low-risk disease or ensuring a more comprehensive procedure is done when high-risk features are present.  You can ask your surgeon: "Will you be using frozen section analysis during my surgery to guide the lymph node procedure?" "How will the frozen section result impact the decision about my ovaries (if we are considering preservation)?" "What is the process at your hospital for communication between pathology and the OR?"  Understanding this process can help demystify what happens while you are asleep and show how the surgical team works dynamically to provide you with the most precise care. |
| What exactly does "comprehensive staging surgery" include? Why are these steps necessary? | “Comprehensive staging surgery” sounds grand and intimidating, but its job is actually very prosaic: figure out exactly how far the cancer has gone, so you’re neither under-treated nor over-treated. Cancer hates vagueness. Staging is how we take that away from it.  Here’s what it usually includes, and—more importantly—*why each piece exists*.  First, the core operation: removal of the uterus (hysterectomy). This removes the primary tumor and allows the pathologist to examine how big it is, what type it is, and how deeply it invades the uterine muscle. Depth of invasion turns out to be one of the strongest predictors of spread. You cannot infer this reliably from imaging alone.  Second, removal of the fallopian tubes and often the ovaries. This is partly about eliminating possible microscopic spread, and partly about prevention. Endometrial cancer can spread to the adnexa even when everything looks normal. The tubes are also now known to be a common origin site for future gynecologic cancers. From a staging perspective, their pathology matters.  Third, assessment of lymph nodes. This is where modern staging has evolved the most.  Traditionally, surgeons removed many pelvic (and sometimes para-aortic) lymph nodes. That gave information, but at the cost of lymphedema and nerve injury. Today, in many patients, this is replaced by sentinel lymph node biopsy, which targets the first nodes that drain the uterus. These nodes are then examined extremely carefully (ultrastaging), often detecting tiny metastases that bulk removal could miss.  Why lymph nodes matter: If cancer cells have reached the nodes, the disease is no longer “uterus-only.” That single fact can change the stage and whether you need radiation or chemotherapy afterward.  Fourth, inspection of the abdomen and pelvis. The surgeon visually surveys the ovaries, bowel surfaces, omentum, and peritoneum. If anything looks suspicious, a biopsy is taken. Cancer doesn’t always read textbooks; staging surgery is partly about catching surprises.  In selected cases—especially higher-risk histologies—omentectomy or peritoneal biopsies may be added. This isn’t routine for low-risk diseases, but for aggressive subtypes, skipping it would leave blind spots.  Now the philosophical question: why do all this instead of “just removing the uterus”?  Because treatment decisions depend on stage, not hope.  Adjuvant therapy (radiation, chemotherapy, both, or neither) is chosen based on: – Depth of invasion – Lymph node status – Tumor type and grade – Presence or absence of extrauterine disease  Without comprehensive staging, doctors are forced to guess—and guessing leads either to undertreatment (higher recurrence risk) or overtreatment (unnecessary toxicity).  One subtle but important point: Staging surgery doesn’t automatically mean “more surgery.” With sentinel node techniques and minimally invasive approaches, modern staging is often *less destructive than older, incomplete operations that later forced broader radiation “just in case.”  So think of comprehensive staging as an information-gathering mission with surgical tools. Its purpose isn’t to be maximal. It’s to be precise.  Cancer therapy works best when it knows exactly what it’s dealing with. Staging is how we stop fighting shadows and start fighting facts. | Excellent question. "Comprehensive staging surgery" is the precise, systematic procedure performed to treat endometrial cancer and determine its exact extent (stage). This information is the single most important factor in predicting your prognosis and guiding all future treatment decisions.  Here is a breakdown of what it typically includes, presented in the general order of the operation, and the critical reasons for each step.   The Components of Comprehensive Staging Surgery  `mermaid flowchart TD A [Comprehensive Staging Surgery] --> B1 [Exploration & Biopsies] A --> B2 [Remove Primary Cancer Site] A --> B3 [Assess Lymph Nodes] A --> B4 [Remove Other Tissues if Needed]  B1 --> C1 [Collect Peritoneal Washings<br>Detect microscopic spread] B1 --> C2 [Inspect/ Biopsy Suspicious Areas<br>Rule out visible spread]  B2 --> C3 [Total Hysterectomy<br>Remove uterus & cervix] B2 --> C4 [Bilateral Salpingo-oophorectomy<br>Remove ovaries & tubes]   B3 --> C5 [Pelvic & Para-aortic LN Assessment<br>Check nodal spread SLNB or LND]   B4 --> C6 [Omentectomy for High-Grade Types<br>Serous/clear cell cancers] B4 --> C7 [Peritoneal Biopsies<br>For advanced disease]  C1 & C2 & C3 & C4 & C5 & C6 & C7 --> D [Final Pathology Report & FIGO Stage] D --> E [Guides Adjuvant Therapy Decision<br>Radiation, Chemotherapy, or Observation] `   Phase 1: Exploration & Biopsies What: The surgeon begins by thoroughly inspecting the entire abdominal and pelvic cavity. They collect peritoneal washings (instilling and removing saline fluid) and inspect/biopsy any suspicious areas on other organs (e.g., peritoneum, diaphragm). Why: This checks for microscopic or visible cancer cells that have spread outside the uterus, which would immediately indicate Stage III or IV disease. The washings are analyzed for cancer cells.   Phase 2: Removal of the Primary Cancer Site & Hormone Sources What:  1. Total Hysterectomy: Removal of the uterus and cervix.  2. Bilateral Salpingo-oophorectomy: Removal of both fallopian tubes and ovaries (as discussed, This may be modified in select young patients). Why: This is the curative core of the surgery, removing the organ housing the cancer and, in most cases, the source of estrogen that could fuel any remaining cells.   Phase 3: Assessment of Lymph Nodes What: Evaluation of the pelvic and para-aortic lymph nodes. As discussed, this is most commonly done via Sentinel Lymph Node Biopsy (SLNB). In high-risk cases or if SLNs are not found, a more extensive lymphadenectomy may be performed. Why: This is the most critical part of staging. Lymph nodes are the primary pathway for cancer spread. Knowing if they contain cancer cells determines whether the cancer is Stage I/II (node-negative) or Stage IIIC (node-positive), which dramatically changes post-operative treatment.   Phase 4: Removal of Other Tissues for High-Grade Cancers What: For high-risk histological types (like serous or clear cell carcinoma), the surgeon may also perform an omentectomy (removal of the fatty apron hanging from the colon) and take multiple peritoneal biopsies. Why: These aggressive cancer types behave like ovarian cancer and have a higher tendency to spread within the abdominal cavity. This step is necessary for accurate staging in these cases.   Why Are These Steps Necessary? The "Why" Behind the Protocol  1. To Determine the FIGO Stage: This is the internationally recognized staging system. The stage (e.g., IA, IB, II, IIIA, IIIC1, IVB) is directly derived from the surgical findings. It is not a guess; it is a precise classification based on what is seen and confirmed under the microscope.  Stage I: Confined to the uterine body.  Stage II: Involves the cervical stroma.  Stage III: Local/regional spread (to adnexa, vagina, nodes).  Stage IV: Spread to the bladder/bowel or distant sites.  2. To Guide Adjuvant (Post-Operative) Therapy: This is the most important practical reason. The stage and pathology tell your oncologist if you need further treatment.  Low-Risk (Stage IA, Grade 1–2): Often, surgery alone is curative. and no further therapy is needed.  Intermediate-Risk: May require vaginal brachytherapy (internal radiation) to reduce the risk of local recurrence in the vagina.  High-Risk (e.g., Stage I with deep invasion & Grade 3, or node-positive): Usually requires external beam radiation and/or chemotherapy to treat potential microscopic disease elsewhere.  3. To Provide Prognostic Information: The stage and grade offer the best estimate of the likelihood of cure and the risk of recurrence.  4. To Ensure Complete Cancer Removal: The goal is to remove all visible and microscopic disease in one procedure, offering the best chance for a cure.   Summary Comprehensive staging surgery is a diagnostic and therapeutic "one-stop" procedure. It is designed to: Remove all cancerous tissue (the uterus, cervix, and often tubes/ovaries). Gather all necessary data (from lymph nodes, washings, and biopsies). Synthesize that data into a precise FIGO stage. Inform a clear, evidence-based path for what comes next: observation, radiation, or chemotherapy.  It is a meticulously planned operation where every step has a proven purpose in maximizing your cure rate and personalizing your follow-up care. Your gynecologic oncologist will tailor the exact extent of the surgery based on your preoperative imaging and the intraoperative frozen section findings, ensuring you receive the most appropriate procedure—neither too little nor too much. |
| If I am an early-stage patient, is "fertility-sparing treatment" possible? What conditions must be met? How high are the risks? | Yes—fertility-sparing treatment *can be possible for some early-stage endometrial cancer patients, but only inside a very narrow biological box. This is not a lifestyle choice; it’s a risk-managed medical strategy. Outside that box, it becomes unsafe quickly.  Let’s be precise.   What “fertility-sparing treatment” actually means  It means not removing the uterus initially, and instead treating the cancer with progestin-based hormonal therapy, while monitoring obsessively. The goal is temporary disease control so pregnancy is possible—not permanent cancer eradication.  This is delay with supervision, not avoidance.   The conditions that must be met (all of them) are: Fertility-sparing treatment is considered only if every box below is checked:  1. Cancer type  – Endometrioid adenocarcinoma  – Grade 1 (low grade) only  High-grade, serous, clear cell = no.  2. Stage  – Stage IA  – Cancer confined to the endometrium  – No myometrial invasion on MRI  3. No spread  – No lymph node involvement  – No ovarian or extrauterine disease  – Imaging must be clean  4. Hormone responsiveness  – Tumor expresses progesterone receptors  (Most grade-1 tumors do, but this matters.)  5. Strong desire for fertility  – This option exists to preserve childbearing, not anatomy.  6. Ability to comply with surveillance  – Regular biopsies every 3–6 months  – Imaging as needed  – Immediate surgery if progression occurs  If even one of these fails, fertility-sparing treatment becomes a gamble with bad odds.   What the treatment involves: Typically: • High-dose oral progestins and/or • A levonorgestrel-releasing IUD • Sometimes both together (better response rates)  This is active cancer therapy, not “watch and wait.”   How effective is it?  Here’s the honest math, averaged across studies:  • Initial response rate: ~70–80% • Complete remission: ~60–70% • Recurrence after remission: ~30–50% • Progression to higher stage: uncommon but real • Overall survival: very high *when strict criteria are met*  Many patients achieve remission, get pregnant (often with assisted reproduction), and then proceed to hysterectomy afterward.   The risks—no sugarcoating  • Cancer persistence: it may not respond • Recurrence: common, even after remission • Progression risk: low but not zero • Anxiety and constant monitoring • You are borrowing time from a disease that prefers certainty  This is why most guidelines recommend definitive surgery after childbearing, even if everything goes well.   What fertility-sparing treatment is NOT—It is not safer than surgery—It is not permanent cancer control—It is not appropriate for “borderline” cases—It is not something to try casually   Bottom line  Fertility-sparing treatment is medically legitimate, tightly controlled, and biologically selective. For the right patient, it offers a real chance at pregnancy without compromising survival. For the wrong patient, it delays curative treatment and increases danger.  So the real decision isn’t “Can I keep my fertility?” It’s “Does my tumor’s behavior allow me to negotiate with time?”  In oncology, that question always has an answer—but it’s written in pathology, imaging, and probabilities, not hope. | This is the most critical question for a young woman facing an endometrial cancer diagnosis. Let's address it with clarity and depth.  In short: Yes, fertility-sparing treatment is possible for a very specific subset of early-stage patients, but it is a calculated risk and a rigorous process, not the standard of care.  It is considered an exception to the standard treatment (hysterectomy) and is only pursued under strict protocols. Here is a detailed breakdown.   Conditions That MUST Be Met (All of Them)  This is a non-negotiable checklist. You must be a candidate for "Fertility-Sparing Management" (FSM).  1. Strong Desire for Future Fertility: This is the foundational reason for considering this path. 2. Young Age: Typically under 40, and certainly premenopausal. The closer to natural menopause, the less benefit. 3. Tumor Type & Grade:  Endometrioid Adenocarcinoma ONLY (the most common type).  Grade 1 (Well-Differentiated) ONLY. Grade 2 is controversial and rarely considered; Grade 3 is an absolute exclusion. 4. Confirmed Early Stage (IA):  No Myometrial Invasion: Imaging, preferably a pelvic MRI, must show the cancer is confined to the endometrium (the uterine lining) with no invasion into the underlying muscle wall.  No Evidence of Spread: Imaging must show no signs of disease in the cervix, ovaries, lymph nodes, or elsewhere. 5. No Contraindications:  No Suspicion of Lynch Syndrome or Hereditary Cancer: These genetic conditions require a different, more aggressive approach. Genetic counseling/testing may be recommended.  No other medical conditions that would make pregnancy high-risk.   The Fertility-Sparing Treatment Protocol (Not a One-Time Fix): This is a prolonged, active treatment and monitoring process, not a single procedure.  1. Initial Expert D&C: A dilation and curettage (D&C), performed by an experienced gynecologic oncologist, is mandatory. This both confirms the diagnosis by providing ample tissue and is the first therapeutic step to remove all visible tumor. 2. Progestin Therapy: The mainstay treatment is high-dose progestin to counteract estrogen and cause the cancer to regress.  Options: Oral (e.g., Megestrol acetate) or via a levonorgestrel-releasing IUD (Mirena®). Often, both are used together for maximum effect. 3. Rigorous Follow-Up & Monitoring:  Schedule: Endometrial biopsies are typically done every 3–6 months.  Goal: To achieve a complete pathological response (no evidence of cancer in the biopsy). This can take 6-12 months or longer.  What if it doesn't work? If cancer persists after 6-12 months of treatment, FSM is usually considered a failure, and a hysterectomy is strongly recommended. 4. Pregnancy Planning:  Once a complete response is confirmed, conception should be attempted as soon as possible, often with the help of a reproductive endocrinologist (fertility specialist). Delays increase the risk of recurrence.  Pregnancy itself has a protective effect due to high progesterone levels. 5. Definitive Surgery After Childbearing: Completion hysterectomy (with removal of tubes and ovaries) is strongly recommended after completing childbearing due to the high risk of recurrence.   How High Are the Risks? A Transparent Look  This path requires accepting significant, known risks.  Risk of Non-Response/Persistence: About 20–30% of tumors will not regress completely with progestin therapy, requiring a hysterectomy. Risk of Recurrence: This is the biggest risk.  During Treatment: Possible, which is why frequent biopsies are non-negotiable.  After Complete Response: Very high—approximately 30–40% of women will have a recurrence, often within the first 2–3 years.  Silver Lining: Most recurrences (70–80%) are again early-stage, low-grade, and many will respond to a second round of progestin therapy or can then be treated with hysterectomy. The vast majority of recurrences do not compromise survival if caught early through strict follow-up. Risk of Undetected Advanced Disease: The primary weakness of FSM is that full surgical staging (lymph node assessment) is not performed. There is a very small risk that an occult, higher-risk disease exists. This is why impeccable imaging (MRI) is critical. Risk of Fertility Challenges: Endometrial cancer is often linked to conditions like PCOS and chronic anovulation, which themselves cause infertility. You must be prepared for the possibility of needing assisted reproductive technologies (like IVF).   Summary & Your Immediate Next Steps  1. Confirm Your Eligibility: Your first step is to get a pelvic MRI and have your pathology slides reviewed by a gynecologic pathology specialist to confirm Grade 1 endometrioid histology. 2. Consult a Specialized Team: You need a multidisciplinary team including a Gynecologic Oncologist and a Reproductive Endocrinologist (Fertility Specialist). They will review all your data against the strict criteria. 3. Have the "Risk-Benefit" Talk: If you are a candidate, have a brutally honest conversation with your doctors. You must understand and accept the risks of recurrence and the commitment to intense monitoring. 4. Consider Genetic Testing: Discuss genetic counseling to rule out Lynch syndrome.  Final Perspective: Fertility-sparing treatment is a safe and viable option only when managed within strict protocols by expert teams for highly selected patients. It trades the immediate certainty of a cure (from hysterectomy) for the chance to carry a pregnancy, accepting a defined and managed level of risk. The decision is profoundly personal and must be made with full information and expert guidance. |
| Is robotic surgery better than traditional laparoscopy? Is it worth the significantly higher cost? | Blunt answer first: robotic surgery is not inherently “better” than traditional laparoscopy for endometrial cancer. In most early-stage cases, outcomes are essentially the same, while the cost is meaningfully higher. The robot is a tool, not a magic spell.  Now let’s replace marketing fog with physics and data.  What the robot actually adds Robotic systems give the surgeon: – Wristed instruments with more degrees of freedom – Tremor filtration – 3D, high-definition visualization – Better ergonomics (the surgeon is less physically strained)  These advantages help the *surgeon*, not the cancer directly.  What it does NOT automatically add: Better cancer clearance, Better survival, Lower recurrence, Better staging accuracy  For early-stage endometrial cancer, multiple studies show: robotic ≈ laparoscopic in oncologic outcomes.  Same disease control. Same staging accuracy. Same survival.  So why do people use robotics at all? Because surgery happens in real bodies, not ideal textbooks.  Robotics can be genuinely helpful when: – The patient has obesity (very common in endometrial cancer) – The pelvis is narrow or anatomy is challenging – The surgery is technically complex – The surgeon is significantly more experienced with robotics than laparoscopy  In these scenarios, robotics can reduce: – Conversion to open surgery – Blood loss – Surgeon fatigue (which quietly matters over long cases)  Notice the theme: robotics lowers technical friction, not biological risk.  Cost—this is where honesty matters: Robotic surgery costs substantially more due to: – Capital equipment – Disposable instruments – Maintenance – Longer operating room time in some settings  From a pure value standpoint: – If both approaches are feasible – And the surgeon is equally skilled in both → laparoscopy usually wins on cost-effectiveness  That’s not ideology. That’s arithmetic.  The uncomfortable truth most patients aren’t told: The surgeon’s skill and judgment matter far more than the platform.  A highly experienced laparoscopic surgeon will outperform: – An inexperienced robotic surgeon – Or a robotic surgeon using the robot as a crutch  Robotics can *extend a surgeon’s capabilities, but it cannot replace them.  How to think about “worth it” Robotics may be worth the cost if: – It meaningfully reduces your risk of open surgery – It allows your surgeon to operate at their highest level – Your anatomy makes standard laparoscopy borderline  It is probably not worth the extra cost if: – You are a straightforward early-stage case – Your surgeon is excellent at laparoscopy – The robot is used mainly because it’s available, not necessary  Bottom line Robotic surgery is not superior by default. It is situationally advantageous. In early-stage endometrial cancer, it rarely improves cancer outcomes compared to laparoscopy, but it may improve technical ease in certain patients.  So the real question isn’t “Is the robot better?” It’s “Which approach allows *this surgeon* to perform *this operation* most safely on *this body*?”  Technology doesn’t cure cancer. Good decisions do. | This is a highly relevant and debated question in modern surgery. The answer isn't a simple "yes" or "no," but rather "it depends on the situation, the surgeon, and the specific goals." Let's break down the comparison and the cost-benefit analysis.   Robotic Surgery vs. Traditional Laparoscopy: A Feature Comparison  \| Feature \| Traditional Laparoscopy \| Robotic Surgery (e.g., Da Vinci) \| \| : \| : \| : \| \| Visualization \| 2D view on a monitor. \| High-definition 3D view with up to 10x magnification. \| \| Instruments \| Rigid, straight tools. Limited range of motion (like operating with chopsticks). \| Wristed, articulating instruments that mimic the full range of motion of a human hand. \| \| Surgeon Control \| Direct hand movement. Hand tremor is transmitted. \| Surgeon sits at console, controls robotic arms. Tremor filtration and motion scaling for precision. \| \| Ergonomics \| Can be physically taxing for the surgeon, leading to fatigue over long procedures. \| The surgeon operates seated at an ergonomic console, reducing fatigue. \| \| Learning Curve \| Steep, but well-established. \| Often considered easier to learn for surgeons already skilled in open surgery, due to intuitive hand-eye coordination and a 3D view. \| \| Tactile Feedback \| Direct "feel" of tissue (haptics). \| No true haptic feedback. The surgeon relies on visual cues for tissue tension and resistance. \|     Is Robotic Surgery "Better"?  For certain procedures and situations, robotic assistance offers distinct advantages:  1. In Complex or Confined Spaces: For surgeries requiring intricate dissection (e.g., around major blood vessels, deep in the pelvis, or for complex cancer staging including para-aortic lymph node dissection), the 3D vision, enhanced dexterity, and precision of the robot can be superior. 2. For Surgeons with Less Laparoscopic Experience: It can help surgeons who are experts in open surgery transition more safely and effectively to minimally invasive techniques. 3. In patients with a high BMI (obesity): The robotic arms are stable and can operate effectively in the deep pelvis, which can be technically challenging with straight laparoscopic tools.  However, for many standard procedures, outcomes are often equivalent. For a straightforward hysterectomy and staging in a patient with average anatomy, a highly experienced laparoscopic surgeon can achieve identical cancer outcomes, safety, and recovery times as with a robot. Numerous studies have shown that when surgeons are proficient in both, the long-term results for the patient are the same.   The Crucial Question: Is it Worth the Higher Cost?  This is the core of the debate. The cost difference is substantial due to the robot's high purchase price (over $1 million) and expensive disposable instruments for each case.  A cost-benefit analysis must consider:  Direct Medical Costs: Higher for robotic surgery. Indirect/System Costs: *Maybe offset by:  Shorter Hospital Stays (though both MIS approaches have short stays).  Lower Conversion Rates to Open Surgery: Some studies show that robots have a lower rate of needing to convert to a large open incision, especially in complex cases or obese patients. Avoiding open surgery saves significant costs from longer hospitalization and higher complication rates.  Surgeon Fatigue: May allow surgeons to perform more complex cases or operate longer with maintained precision.  From a *Patient's Perspective: What Matters Most?  For you, the most important factors are safety, completeness of cancer surgery, and recovery. The technology itself is a tool. The single most critical factor is your surgeon's skill and experience.   Key Takeaways & Questions to Ask Your Surgeon  1. The Surgeon is More Important Than the Machine. Choose a surgeon highly experienced in minimally invasive gynecologic oncology. Ask about their specific training and volume. 2. Ask About Their Approach: "For a case like mine, do you more commonly use traditional laparoscopy or the robot?" What are the reasons you would choose one over the other for my specific situation? 3. Focus on Outcomes, Not Technology: Ask: "What is your personal rate of converting to open surgery? What are your complication rates?" A skilled surgeon will have excellent outcomes with either tool. 4. Cost Transparency: Ask your hospital's financial counseling department about your potential out-of-pocket costs for robotic vs. laparoscopic surgery, as insurance coverage can vary.   Final Verdict: Robotic surgery is a powerful technological advancement that expands the possibilities of minimally invasive surgery, especially in complex cases, and can help more surgeons offer MIS options. It is not categorically "better" than skilled traditional laparoscopy for all patients in terms of final cancer outcomes and recovery. The value of the investment is most clear in enabling complex surgeries to be done minimally invasively, reducing conversions to open surgery, and potentially shortening the learning curve for surgeons.  Your best path is to find a surgeon you trust and follow their recommendation on the optimal approach for your specific anatomy and cancer characteristics. They should be able to clearly explain why their preferred method (lap or robot) is the right tool for your operation. |
| How long will the surgery take? Are the risks of general anesthesia high? | Here’s the practical reality, no fluff:   Surgery duration  For early-stage endometrial cancer using minimally invasive approaches (laparoscopy or robotic):  Typical operating time: 1.5–3 hours Factors that can extend time:   Obesity or complex anatomy  Deep or large tumors  Sentinel lymph node mapping or full node dissection  Unexpected findings that require additional steps  For open surgery (laparotomy): 2–4 hours is common, sometimes longer if lymph nodes or extra tissue need removal.  Minimally invasive surgery generally takes slightly longer than open surgery in operating time but shortens recovery time significantly.     Risks of general anesthesia  For most young, healthy patients, anesthesia is extremely safe. Modern anesthetics and monitoring make major complications rare.  Potential risks include:  Temporary nausea, vomiting, or sore throat Minor allergic reactions Rare serious complications:   Breathing problems  Heart rhythm changes  Blood clots  Stroke or heart attack (very rare in young, healthy patients)  Factors that increase anesthesia risk:  Older age Obesity Heart, lung, or kidney disease History of difficult anesthesia Smoking  An anesthesiologist reviews your history and tailors drugs and monitoring to minimize risk. For most young patients with early-stage endometrial cancer, serious complications are uncommon (<1%).    Bottom line:  Surgery usually takes 1.5–3 hours minimally invasively. General anesthesia is very safe in young, healthy patients. Most risks are minor or temporary, but your anesthesiologist is there to handle anything unexpected.  If you want, I can give a step-by-step timeline of what your day of surgery and recovery might actually look like, so you know what to expect hour by hour. | These are two of the most common and important questions for anyone facing surgery. Let's address them clearly.   1. How Long Will the Surgery Take?  For a comprehensive staging surgery for endometrial cancer, you should plan for the entire process—from being taken into the operating room to waking up in the recovery area—to take approximately 2 to 4 hours.  Here’s a typical breakdown of where that time goes:  Preparation & Anesthesia (30–45 minutes): Getting positioned, placing monitors, IVs, and safely administering anesthesia. The Surgical Procedure Itself (1.5 to 3 hours): This is the variable part. It depends on:  Surgical Approach: Minimally invasive (laparoscopic/robotic) surgery often takes longer than open surgery due to the setup and precise dissection, but recovery is faster.  Extent of Surgery: A simple hysterectomy is quicker than a full-staging procedure with sentinel lymph node mapping and biopsy or a full lymph node dissection.  Patient Factors: Anatomy, body habitus (e.g., BMI), and the presence of scar tissue from prior surgeries can add time.  Surgeon's Findings: If unexpected disease is found (e.g.,, adhesions, more advanced cancer), the procedure will be extended to complete the necessary staging. Emergence from Anesthesia & Transfer (30–45 minutes): Gradually waking up, ensuring stable vital signs, and moving to the recovery room.  Key Point: Don't be alarmed if your surgery takes longer than initially estimated. A careful, meticulous surgery is the priority. Your surgical team will keep your family updated.   2. Are the Risks of General Anesthesia High?  This is a very understandable concern. The short answer is: For the vast majority of people, modern general anesthesia is extremely safe. The risks are low, but not zero. The entire anesthesia team is dedicated to minimizing these risks.  Here’s a transparent look at the risks and the extensive safety measures in place: Common, Minor Side Effects (Very Frequent, Temporary): Nausea and vomiting, Sore throat (from the breathing tube) Shivering and feeling cold Confusion or "brain fog" upon waking (more common in the elderly)  # Serious Complications (Very Rare): These are what people truly fear. It's crucial to understand their rarity and the context: Death or Major Brain Damage: The risk of death directly attributable to anesthesia is extraordinarily low—estimated at less than 1 in 100,000 to 200,000 for healthy patients. This risk is often tied to the complexity of the surgery itself, not the anesthesia alone. Heart Attack or Stroke: The risk is primarily related to your pre-existing health conditions (like heart disease). The anesthesia team meticulously manages your blood pressure and heart function to prevent this. Awareness During Surgery (Anesthesia Awareness): Extremely rare (approx. 1 in 10,000-20,000), and even rarer to have pain or recall. Brainwave monitors are often used to ensure you are fully unconscious. Allergic Reaction or Breathing Problems: The team is trained to recognize and treat these immediately.  # How Your Risk is Minimized: The Safety Protocol  Your safety is managed through a multi-step process: 1. Pre-Operative Assessment: You will meet with an anesthesia provider (an anesthesiologist or nurse anesthetist) before surgery. They will review your entire medical history, medications, allergies, and any prior issues with anesthesia. This is your chance to ask all your questions. 2. Individualized Plan: Your anesthesia is tailored to you—your age, weight, health status, and the planned surgery. 3. Constant Monitoring: From induction to waking, you are monitored with:  Continuous EKG (heart)  Blood pressure (every few minutes)  Oxygen saturation (pulse oximeter)  End-tidal CO2 (breathing adequacy)  Often, depth of anesthesia monitors (BIS/EEG). 4. Expert Team: A dedicated anesthesia professional stays with you for the entire surgery, doing nothing but managing your vital signs and comfort.  Your personal risk level depends largely on your overall health (e.g., pre-existing heart, lung, or kidney disease, smoking, obesity, sleep apnea) rather than the anesthesia itself.   Your Action Plan: Questions for Your Care Team  To alleviate your specific concerns, ask your surgeon and anesthesia provider:  "Based on my health history, what is my personalized risk profile for anesthesia?" "Who will be providing my anesthesia, and can I speak with them beforehand?" "What specific steps will you take to prevent post-op nausea?" (They have excellent medications for this.) "How long should I expect to be in the recovery room before going to my hospital room?"  Bottom Line: While any surgery with anesthesia carries inherent risks, for endometrial cancer staging surgery, the benefits of a properly staged and treated cancer far outweigh the very small risks of a modern, expertly administered anesthetic. The system is designed with multiple layers of safety to protect you. |
| How is the completeness of the surgery assessed? How can it be ensured that the tumor is fully removed? | In endometrial cancer surgery, “completeness” doesn’t just mean cutting out the uterus—it’s about making sure all cancer that could affect prognosis is removed or properly staged. There are several layers to how surgeons ensure this.     1. Preoperative planning: Before you even go to the OR, your surgeon relies on:  MRI or CT imaging to see how deep the tumor invades and whether lymph nodes look suspicious. Endometrial biopsy to confirm tumor type and grade. Blood tests and sometimes tumor markers.  Good imaging + pathology reduces the chance of surprises in surgery.     2. Intraoperative assessment: During surgery, the surgeon actively evaluates:  The uterus and tumor: visually and sometimes palpably. Surrounding structures: ovaries, fallopian tubes, peritoneum, and omentum. Sentinel lymph nodes (or full lymph node dissection if indicated) are used to see if microscopic spread is present.  Frozen section pathology may be used to assess:  Tumor grade Depth of myometrial invasion  This helps decide whether more extensive lymph node assessment or additional resections are needed.     3. Surgical technique: Completeness depends on following oncologic principles:  Total hysterectomy: removes the uterus and cervix. Removal of fallopian tubes ± ovaries: eliminates potential microscopic spread. Lymph node assessment: sentinel node biopsy or dissection ensures proper staging. Inspection or biopsy of suspicious areas in the abdomen or pelvis.  The goal is not just “get it out” but to remove or sample all areas where cancer might hide.     4. Postoperative pathology: After surgery, the removed tissue is examined thoroughly:  Margins: confirms the tumor hasn’t reached the edge of the excised tissue. Depth of invasion: critical for prognosis and adjuvant therapy decisions. Lymph nodes: ultrastaging identifies micrometastases. Other tissues (ovaries, tubes, peritoneum) if removed or biopsied.  This is the final confirmation that surgery achieved its goals. If pathology shows residual cancer or unexpected spread, further treatment (e.g., radiation or chemotherapy) is planned.     5. Why “100% assurance” is rarely guaranteed  Cancer is microscopic. Surgeons can remove all visible and reachable disease, but they rely on staging and pathology to detect tiny clusters of cells. That’s why surgery is paired with follow-up imaging and, if indicated, adjuvant therapy.  In other words, “complete removal” = all macroscopic disease + proper staging, not a literal guarantee of zero microscopic cells in your body.     Bottom line: Surgical completeness is a combination of:  1. Careful pre-op planning 2. Meticulous removal and lymph node assessment 3. Real-time evaluation (visual, palpation, frozen section) 4. Thorough post-op pathology: This layered approach maximizes the chance that the tumor is fully addressed, while follow-up and adjuvant therapy manage any residual microscopic risk.  If you want, I can draw a diagram showing exactly what tissues and nodes are removed or examined in a standard early-stage surgery—it makes “completeness” very concrete. | This is arguably the most important question a patient can ask. Ensuring the "completeness" of cancer surgery is the core goal of your surgical team. It's not a single event but a multi-step process of assessment and verification that happens during and after your operation.  Here is how surgeons ensure the tumor is fully removed, from the operating room to the final pathology report.   Part 1: Intraoperative Assessment (During Surgery)  This is the real-time quality control performed while you are still under anesthesia.  1. Visual and Tactile Inspection:  The surgeon meticulously inspects the entire pelvic and abdominal cavity for any signs of visible disease beyond the uterus.  They examine the removed uterus, ovaries, and tubes for gross (visible) tumor characteristics and margins.  2. Frozen Section Analysis (The Crucial Real-Time Tool):  As discussed, this is a rapid pathology check. The surgeon sends the uterus to the pathology lab.  The pathologist examines key areas, most importantly:  Tumor Grade: Is it the expected low-grade tumor, or is it more aggressive?  Depth of Invasion: How deep has the cancer grown into the uterine wall? This is the single most important factor for predicting spread.  Cervical Involvement: Is the cancer touching the cervix? (This changes the stage and surgical plan).  Based on this, the surgeon makes critical decisions: If the frozen section shows deep invasion or high-grade features, they will proceed with a more comprehensive lymph node assessment to check for spread, as the risk is higher.  3. Lymph Node Evaluation (Sentinel Node or Dissection):  The status of the lymph nodes is the ultimate test of whether cancer has escaped the uterus.  If the sentinel nodes are negative on frozen section, it provides high confidence that the cancer is contained.  If any node is positive, the surgeon knows the cancer has spread regionally (Stage IIIC) and will remove all nodes in that specific basin for therapeutic purposes.   Part 2: The Final, Definitive Assessment (The Pathology Report): The completeness of removal is ultimately proven in the pathology lab, days after surgery. This is the gold standard.  1. Examination of the Uterus: The entire uterus is processed into slides. The pathologist confirms:  Tumor size and location.  Final Depth of Invasion (measured microscopically in millimeters and as a percentage of the uterine wall).  Distance to Surgical Margins: They check that the cancer is not present at the cut edges (margins) of the cervix or the areas where blood vessels were sealed. For endometrial cancer, clear margins are almost always achieved with a standard hysterectomy.  Lymphovascular Space Invasion (LVSI): This is a critical finding—whether cancer cells are seen in small channels of blood or lymph vessels. Its presence increases the risk of recurrence.  2. Ultrastaging of Lymph Nodes: The few sentinel nodes removed undergo ultrastaging. They are sliced much more thinly and examined with special stains to find even the tiniest deposits of cancer cells (micrometastases) that would be missed in a routine check.  3. Examination of All Other Tissues: Any other tissue removed (ovaries, fallopian tubes, omentum, peritoneal biopsies) is examined to confirm the absence of cancer.   How "Completeness" is Defined and Ensured  For endometrial cancer, a "complete" or "optimal" surgery means:  R0 Resection: No gross (visible) disease left behind. Adequate Surgical Staging: All necessary tissues (uterus, cervix, tubes/ovaries) and lymph node samples have been removed and examined to accurately determine the FIGO stage. Negative Surgical Margins: Cancer is not present at the edges of the removed tissue. No Unresected Metastatic Disease: Any spread that was found (e.g., to nodes) was removed during the surgery.  The final pathology report synthesizes all this data into your FIGO stage (e.g., Stage IA, Grade 1, no LVSI). This stage is the definitive answer to the question of completeness and dictates the next steps.   The Final Safety Net: Adjuvant Therapy—Even with a surgically "complete" removal, microscopic cells can sometimes remain. This is why the final pathology stage guides the need for adjuvant (additional) therapy.  If you are low-risk (e.g.,, Stage IA, Grade 1-2): Surgery alone is considered curative, and no further treatment is needed. The completeness of surgery is definitive. If you have intermediate or high-risk features (e.g., deep invasion, LVSI, Grade 3, positive nodes): Your oncologist will recommend radiation or chemotherapy to eradicate any potential microscopic cells that surgery couldn't remove. This is not a failure of surgery, but a logical extension of care based on the precise staging that the surgery provided.   Summary: The Layers of Assurance  1. Surgeon's Skill & Protocol: An experienced gynecologic oncologist follows a standardized staging procedure. 2. Intraoperative Frozen Section: Guides the extent of surgery in real time. 3. Final Pathology Report: Provides the microscopic proof of completeness and establishes the exact stage. 4. Multidisciplinary Review: Your case is reviewed by a tumor board (surgeons, medical oncologists, radiation oncologists, pathologists) to confirm the treatment plan. 5. Tailored Adjuvant Therapy: Addresses any microscopic risk based on the surgical findings.  You can ask your surgeon: "What specific factors in my final pathology report will tell us if the surgery was complete and if I need any further treatment?" This will direct the conversation to your personalized prognosis and next steps. |
| II. Preoperative Evaluation and Optimization |  |  |
| What tests do I need to complete before surgery? (e.g., MRI, CT, CA125, cardiopulmonary function assessment, etc.) What is the purpose of these tests? | Before endometrial cancer surgery, your medical team orders a series of tests to map the tumor, assess your overall health, and plan anesthesia safely. Each test has a purpose—nothing is random. Here’s a clear breakdown:     1. Imaging to evaluate the cancer: a. Pelvic MRI (preferred)  Purpose: Shows the depth of tumor invasion into the uterine muscle, whether the tumor extends to the cervix, and helps detect suspicious lymph nodes. Why is it important: It determines whether fertility-sparing treatment is even possible and helps plan the surgical approach (minimally invasive vs open).  b. Pelvic/abdominal CT scan (sometimes added)  Purpose: Checks lymph nodes outside the pelvis (para-aortic) and other organs for metastasis. Why is it important: Ensures surgery targets all possible sites if higher-stage disease is suspected.  c. Chest imaging (X-ray or CT)  Purpose: Screens for lung metastases in higher-risk cases. Why it is important: It guides whether additional treatment (like chemo) may be needed.     2. Blood tests: a. Complete blood count (CBC)  Purpose: Checks for anemia or infection risk, important for surgery and recovery.  b. Liver and kidney function tests  Purpose: Ensures your organs can safely metabolize anesthesia and medications.  c. Tumor markers (CA125, sometimes HE4)  Purpose: CA125 can be elevated in endometrial or ovarian cancer. Not diagnostic alone, but it helps assess disease burden and can be monitored postoperatively.  d. Coagulation profile. Purpose: Checks clotting ability, reducing the risk of bleeding or clot formation during surgery.     3. Cardiopulmonary and anesthesia assessment. a. ECG (electrocardiogram)  Purpose: Screens for heart rhythm issues that could complicate anesthesia.  b. Echocardiogram or stress test (if indicated)  Purpose: Evaluates heart function in patients with a history of heart disease or risk factors.  c. Pulmonary function tests (if indicated)  Purpose: Ensures the lungs can handle anesthesia, especially if you have asthma, COPD, or a smoking history.  d. Pre-anesthesia physical exam  Purpose: Confirms you’re fit for general anesthesia, plans airway management, and addresses any risks.     4. Optional / case-specific tests: Bone density scan: if you may enter early menopause (ovary removal) Endocrine hormone levels: sometimes to guide fertility-sparing therapy Genetic testing: if family history suggests Lynch syndrome or other hereditary cancer risk     Bottom line: Imaging: defines cancer spread → guides surgical approach and fertility options Blood tests & tumor markers: evaluate baseline health, organ function, and risk Cardiopulmonary assessment: ensures anesthesia safety Other tests: tailored to your age, risk factors, and treatment plan  Everything is about reducing surprises in the OR and maximizing safety and effectiveness.  If you want, I can make a step-by-step pre-surgery checklist with timing so you know exactly when each test is done before your operation. | Excellent question. Completing the right preoperative tests is crucial for safe surgery and precise planning. These tests serve two main purposes: 1) accurately stage the cancer, and 2) ensure you are medically fit for anesthesia and surgery.  Here is a detailed list of typical preoperative tests for endometrial cancer surgery and their specific purposes.   Category 1: Tests for Cancer Staging & Surgical Planning: These tests help your surgeon understand the exact nature and extent of your cancer *before entering the operating room.  \| Test \| Purpose in Endometrial Cancer \| \| \| Pelvic MRI (Magnetic Resonance Imaging) \| THE KEY IMAGING STUDY. It provides superb detail of the uterus to assess:<br>• Depth of myometrial invasion (Is the cancer superficial or deep into the muscle wall?) This is the single most important factor for planning lymph node assessment.<br>• Cervical stromal involvement (Is cancer in the cervix? This changes the surgical plan).<br>• Gross evaluation of lymph nodes (Are any nodes visibly enlarged?).<br>• Rules out obvious spread to other pelvic structures. \| \| CT Scan of Chest/Abdomen/Pelvis \| Often used if the MRI suggests deep invasion, high-grade tumor, or if CA-125 is very elevated. Its purpose is to look for distant metastasis (spread) to the lungs, liver, or distant lymph nodes. It is less detailed for the uterus itself than MRI. \| \| CA-125 Blood Test \| A tumor marker. While not diagnostic, a significantly elevated level can be a red flag for more advanced disease (e.g., spread outside the uterus) or a more aggressive tumor type (like serous carcinoma). A normal level is common in early-stage disease. \|   Category 2: Tests for Medical & Anesthesia Fitness  These are standard for any major surgery to minimize risks.  \| Test \| Purpose \| \| : \| : \| \| Complete Blood Count (CBC) \| Checks for anemia (low red blood cells), which could affect oxygen delivery during surgery, and evaluates the platelet count (for clotting). \| \| Comprehensive Metabolic Panel (CMP) \| Evaluates kidney and liver function, electrolytes, and blood sugar. Crucial for medication dosing and fluid management during anesthesia. \| \| Coagulation Panel (PT/PTT/INR) \| Assesses blood clotting ability to prevent excessive bleeding or clotting issues. \| \| Electrocardiogram (EKG) \| Screens for underlying heart conditions that could pose a risk under anesthesia. Routine for patients over 50 or with cardiac symptoms. \| \| Chest X-Ray \| Evaluates heart size and lung fields. Often done for patients with lung disease symptoms, a smoking history, or an abnormal EKG. \| \| Cardiopulmonary Function Assessment \| Not routine for everyone. May be ordered if you have significant heart/lung disease (e.g., severe COPD, heart failure) to quantify your risk and optimize your condition before surgery. \|   Category 3: Consultations & Special Assessments  Pre-Anesthesia Evaluation: A visit with an anesthesiologist or nurse anesthetist to review all your health history, medications, and test results. They will create your personalized anesthesia plan and address any concerns. Cardiology/Pulmonary Consult: Only if you have known complex heart or lung disease. Genetic Counseling Referral: May be recommended if you are young (<50) or have a strong family history of cancer (colon, endometrial, or ovarian) to assess for Lynch syndrome.   Summary: The Typical Pre-Op Pathway  1. For Staging: A pelvic MRI is the cornerstone. A CT scan and CA-125 are added based on initial biopsy results (high-grade features) or MRI findings. 2. For Safety: Blood work (CBC, CMP, coagulation), an EKG, and a chest X-ray (if indicated) are standard. 3. For Optimization: Consultations (anesthesia, etc.) are scheduled to clear you for surgery.   Purpose in a Nutshell: These tests allow your surgical team to create a personalized, precise, and safe surgical plan. They answer: *"What is the most likely stage?"and *"What is the safest way to get this patient through the operation?"*  Key Questions to Ask Your Care Team: "Based on my biopsy, do I need a pelvic MRI, a CT scan, or both?" "What is my CA-125 level, and how does that influence the plan?" "Do I need any special cardiac or pulmonary clearances given my personal health history?" "When will I have my pre-anesthesia appointment to discuss the anesthesia plan?"  By thoroughly completing this preoperative roadmap, your team minimizes surprises in the OR and maximizes your safety and the effectiveness of your cancer surgery. |
| I have chronic conditions like hypertension/diabetes. How should they be optimized before surgery? | With chronic conditions like hypertension and diabetes, careful preoperative optimization is critical. Uncontrolled blood pressure or blood sugar significantly increases surgical and anesthesia risks, including bleeding, infection, heart events, and poor wound healing. Here’s a practical roadmap.     1. Hypertension (high blood pressure)  Goals before surgery:  Aim for stable blood pressure, usually <140/90 mmHg (your surgeon or anesthesiologist may give a specific target). Avoid sudden spikes or drops during surgery, which can cause heart or kidney problems.  Optimization steps:  Medication adherence: Take antihypertensives as prescribed. Do not stop before surgery unless instructed. Review medications: Some blood pressure drugs (like ACE inhibitors) may be paused on the day of surgery; your physician will advise. Lifestyle tweaks: Reduce salt intake, avoid caffeine/stimulants, and ensure adequate sleep. Monitoring: Home or clinic blood pressure checks in the weeks leading up to surgery.     2. Diabetes (high blood sugar)  Goals before surgery:  Maintain blood sugar in a safe target range, often ~100–140 mg/dL fasting pre-op (the exact target is individualized). Reduce the risk of infection, delayed wound healing, and cardiovascular complications.  Optimization steps:  Medication adjustment: Insulin or oral diabetes meds may need timing changes around fasting and surgery; your endocrinologist will guide this. Blood sugar monitoring: Check fasting and postprandial levels consistently in the days before surgery. Diet control: Focus on stable carbohydrate intake and avoid sugary foods. Weight and fluid balance: If overweight or fluid retention exists, mild weight optimization and careful hydration can help.     3. Coordination with your medical team: Pre-op clearance: Your primary doctor or cardiologist/endocrinologist may provide a clearance letter confirming that your conditions are controlled. Medication plan for surgery day: Make a clear list of what to take, what to skip, and when. Close follow-up: Some hospitals schedule a pre-op visit specifically to optimize chronic conditions.     4. Additional practical tips: Bring all current medication bottles to pre-op. Avoid sudden lifestyle changes that might destabilize blood pressure or blood sugar just before surgery. Notify your surgeon and anesthesiologist of any recent hospitalizations, medication changes, or symptoms like chest pain or dizziness.     Bottom line: Hypertension: stabilize blood pressure; avoid spikes or drops. Diabetes: maintain tight but safe glucose control; prevent infection risk. Communication: coordinated care between the surgeon, anesthesiologist, and your primary doctor is essential.  Optimizing these conditions reduces surgical complications, shortens recovery, and improves overall safety.  If you want, I can draft a pre-surgery timeline specifically for patients with hypertension and diabetes, showing exactly how medications, diet, and monitoring should be managed in the two weeks leading up to your operation. | This is an extremely important question. Properly managing chronic conditions like hypertension (high blood pressure) and diabetes before surgery is critical for your safety and a smooth recovery. It reduces the risk of complications during and after the procedure.  Here is a clear, step-by-step guide on how to optimize these conditions before your surgery.   Why Optimization Matters for Hypertension: Uncontrolled high blood pressure increases the risk of heart attack, stroke, heart rhythm problems, and bleeding during surgery. It also makes blood pressure management under anesthesia more difficult. For diabetes: High blood sugar can impair wound healing, increase the risk of infection, and lead to electrolyte imbalances. It can also cause poor outcomes if there are heart or kidney issues.   Pre-Surgery Optimization Plan  The key is to start this process as soon as possible, ideally weeks before your surgery date. Your surgical team, primary care doctor, and/or specialist (cardiologist/endocrinologist) will work together.  # 1. For Hypertension (High Blood Pressure), Goal: Achieve well-controlled blood pressure as per your doctor's target (typically < 130/80 mmHg for most adults before elective surgery). Actions:  Medication Review: Do NOT stop your blood pressure medications unless specifically instructed by your surgeon or cardiologist. Most are continued right up to and including the morning of surgery with a small sip of water.  Special Note on Certain Drugs: Medications like ACE inhibitors (e.g., lisinopril) or ARBs (e.g., losartan), losartan) are sometimes given the morning of surgery to prevent intraoperative low blood pressure. This decision MUST be made by your anesthesiologist or surgeon. You will receive clear instructions.  Lifestyle Measures: Adhere strictly to a low-sodium diet, maintain any prescribed exercise, and monitor your blood pressure at home. Keep a log to show your doctors.  # 2. For Diabetes: Goal: Achieve optimal glycemic control. A common target is an HbA1c < 8.0% for surgery. and ideally much lower (< 7.0%). Fasting and pre-meal blood glucose should be in a safe range (e.g., 80-180 mg/dL). Actions:  Intensify Monitoring: Check your blood sugar more frequently (fasting and after meals). Good data are essential.  Medication Adjustments:  Oral Medications: Some, like metformin, are usually held starting the day before or the day of surgery to reduce the risk of a rare condition called lactic acidosis. You will get specific instructions.  Injectable Medications (Insulin & GLP-1 agonists): This requires a detailed, personalized plan created with your endocrinologist or surgeon.  Long-acting/Background Insulin: The dose is often reduced the night before/morning of surgery.  Short-acting/Mealtime Insulin: Typically held when you are fasting.  GLP-1 agonists (e.g.,, Ozempic, Trulicity): Due to new guidelines about slowed stomach emptying, you will likely be instructed to hold the dose for 1–2 weeks prior to surgery to reduce the risk of aspiration under anesthesia. This is a critical discussion point.  Hospital Protocol: Be prepared for your blood sugar to be managed with an intravenous insulin drip during and immediately after surgery, as it's the safest and most precise method.   Your Action Plan: A Checklist  1. Immediate Notification: Inform your gynecologic oncologist and your anesthesiologist about all your conditions and medications during your first consultations. 2. Schedule a "Medical Clearance" Appointment: See your primary care doctor, cardiologist (for HTN), and/or endocrinologist (for diabetes) specifically for pre-surgical optimization. Bring your surgery date and a list of questions. 3. Create a Unified Plan: Ensure all your doctors communicate. The final instructions on which medications to take or hold on the day of surgery will typically come from the pre-anesthesia clinic or your surgeon's office. 4. Gather Your Records: Bring your home blood pressure logs and blood glucose logs to all appointments. 5. Ask These Specific Questions:  "What are my specific blood pressure and blood sugar targets for surgery?"  "Which of my medications should I take on the morning of surgery?" Which should I hold, and for how long before?"  "Do I need to adjust my insulin doses in the days leading up to surgery? Can you provide me with a written plan?"  "Given my GLP-1 medication, how many days before surgery should I stop it?"   Summary Timeline  Weeks Before: Intensify management with your doctors. Aim for control targets. 1–2 Weeks Before: Finalize medication plans, especially for diabetes medications like GLP-1 agonists. Day Before/Day of: Follow the precise medication instructions given to you. Typically, you take most heart/blood pressure pills with a sip of water, and adjust/hold diabetes meds as directed.  By taking these proactive steps, you are actively participating in your own care and significantly reducing your perioperative risks. Well-managed chronic conditions lead to fewer complications, a smoother anesthesia experience, and a faster recovery. |
| My doctor recommends "neoadjuvant therapy" (such as chemotherapy or radiation) before surgery. Why is this? | Here’s the plain, practical explanation: neoadjuvant therapy is treatment given before surgery to shrink or control the tumor, rather than after. It’s not routine for all endometrial cancers—it’s only recommended in certain situations.     Why your doctor might suggest it  1. The tumor is large or locally advanced   If the tumor has invaded deeply into the uterus or surrounding tissues, it may be difficult or risky to remove completely right away.  Shrinking the tumor first can make surgery safer, easier, and more likely to remove everything.  2. High-risk histology   Aggressive subtypes (serous, clear cell, or high-grade endometrioid) may benefit from early systemic therapy to reduce microscopic spread before surgery.  3. Medical optimization   For patients who aren’t immediately fit for surgery due to comorbidities (like uncontrolled diabetes or heart disease), neoadjuvant therapy can buy time to improve health before the operation.  4. Unresectable disease   If imaging shows a tumor extending to structures that would make surgery extremely difficult or dangerous, preoperative therapy can sometimes convert an inoperable tumor into an operable one.     Types of neoadjuvant therapy: 1. Chemotherapy (systemic drugs)   Works throughout the body to shrink the tumor and target possible microscopic spread.  Usually given in cycles over weeks.  2. Radiation therapy (external beam or brachytherapy)   Focused on the uterus and nearby tissue to shrink the tumor and reduce local invasion.  Sometimes combined with chemotherapy (“chemoradiation”) for high-risk cases.     Benefits of neoadjuvant therapy: Makes surgery less extensive and safer Can improve chances of complete tumor removal Sometimes reduces the need for more radical procedures (like full lymph node dissection) Addresses microscopic disease early, before surgery spreads it     Risks or downsides: Side effects from chemo or radiation (fatigue, nausea, blood count changes, bowel/bladder irritation) Tumor may not shrink as expected. Slight delay in definitive surgery. Requires careful monitoring to ensure timely progression to surgery.     Bottom line: Neoadjuvant therapy is a strategic pre-surgery move, not a punishment. It’s recommended when surgery alone may be risky or incomplete. The decision is always a balance of:  1. How aggressive or large the tumor is 2. Your overall health and surgical fitness 3. Likely benefit vs side effects  It’s a plan to increase your chance of a successful, safe operation while controlling the cancer early.  If you want, I can outline a typical neoadjuvant therapy-to-surgery timeline so you can see exactly how treatment sequences and monitoring usually work. | This is a very important question, and it signals that your doctor is tailoring your treatment plan to a more complex situation. Neoadjuvant therapy (treatment before surgery) is not the standard first step for most early-stage endometrial cancers, but it is a crucial strategy in specific, more advanced scenarios.  Here’s a clear breakdown of why it might be recommended for you, and what it aims to achieve.   Why Neoadjuvant Therapy Instead of Surgery First?  The primary goal is to improve the outcome of surgery and the overall chance of cure by addressing the cancer's aggressive features *before operating. It is typically considered in these situations:  1. To Shrink a Large or Bulky Tumor:  Scenario: If the tumor is very large, it may be technically difficult or risky to remove completely with clear margins.  Goal: Chemotherapy or radiation can shrink the tumor, making it easier, safer, and more effective to remove surgically. This is sometimes called "debulking."  2. For Cancers with a High Risk of Microscopic Spread:  Scenario: For very aggressive cell types like uterine serous carcinoma or carcinosarcoma, which behave like ovarian cancer and have a high tendency to spread early, even if imaging looks normal.  Goal: Systemic chemotherapy before surgery can attack microscopic cancer cells that may have already spread throughout the abdominal cavity (peritoneum) or bloodstream, which surgery alone cannot reach.  3. For Locally Advanced Disease:  Scenario: If imaging (MRI/CT) suggests the cancer has spread locally to the cervix, vagina, bladder, rectum, or nearby lymph nodes, making immediate surgery very radical or incomplete.  Goal: Radiation (with or without chemo) can target and shrink this local spread, potentially converting an inoperable tumor into one that can be surgically removed.   What Forms Does Neoadjuvant Therapy Take?  Neoadjuvant Chemotherapy: This is the most common form. You would typically receive 3–4 cycles of intravenous chemotherapy (e.g., carboplatin/paclitaxel) over a few months, then have surgery, followed by more chemotherapy post-op. Neoadjuvant Radiation Therapy: Less common, but may be used for locally advanced disease, often in combination with low-dose chemotherapy (chemoradiation) to make the radiation more effective.   Potential Benefits of This "Surgery-Later" Approach: Increased Chance of Optimal (Complete) Surgical Removal: By shrinking the tumor first, the surgeon is more likely to achieve an "R0 resection" (no visible cancer left behind). Treating Microscopic Disease Early: Hits cancer cells that may have escaped the pelvis *before they can grow further. May Allow for Less Radical Surgery: In some cases, it might avoid the need for more extensive organ removal (like parts of the bladder/rectum). Acts as an "In Vivo" Sensitivity Test: Seeing how the tumor responds to chemotherapy gives valuable prognostic information. A good response is a positive sign.   Important Considerations & Questions for Your Doctor  This approach means your treatment journey will be longer (several months of chemo before surgery) and requires careful coordination.  You must have a detailed discussion with your gynecologic oncologist and medical oncologist. Ask these key questions:  1. "What specific characteristic of my cancer leads to this recommendation?" (Is it the cell type, the apparent stage on imaging, the tumor size, or genetic markers?) 2. "What is the primary goal of neoadjuvant therapy in my case?" (To shrink the primary tumor? To treat suspected microscopic spread? To avoid overly radical surgery?) 3. "What is the exact treatment plan?" (Which drugs? How many cycles? How will we monitor response? What scans will be used?) 4. "What happens after neoadjuvant therapy?" (Will I definitely have surgery? When? Will I need more treatment after surgery? 5. "What are the potential risks or downsides?" (Could the cancer progress during chemo? Could it make surgery more difficult if there is a poor response?))   Summary  Receiving a recommendation for neoadjuvant therapy means your cancer team believes your situation is more effectively treated by first weakening the cancer throughout the body or shrinking a large tumor, rather than going straight to surgery. It is a strategic, evidence-based approach for locally advanced, bulky, or highly aggressive endometrial cancers.  While it may feel like a delay, it is a planned sequence designed to give you the best possible long-term outcome. Ensure you understand the specific rationale for your case by having the detailed conversation outlined above. |
| Do I need "genetic counseling and testing" (e.g., for Lynch syndrome)? What does this mean for me and my family? | For endometrial cancer, genetic counseling and testing are not automatic for everyone—but they are strongly considered in certain situations, mainly to check for hereditary cancer syndromes like Lynch syndrome. Here’s a clear breakdown.     What Lynch syndrome is: A hereditary condition caused by mutations in DNA mismatch repair (MMR) genes.  People with Lynch syndrome have a higher lifetime risk of:   Endometrial cancer  Colorectal cancer  Ovarian cancer  Certain other cancers (stomach, urinary tract, small bowel, brain)  It’s autosomal dominant, meaning if you have the mutation, each child has a 50% chance of inheriting it.     Who should consider genetic counseling/testing? Guidelines suggest testing for patients who have any of the following:  1. Age <50 at diagnosis 2. Family history of Lynch-associated cancers (colon, endometrial, ovarian, stomach, urinary tract) 3. Multiple cancers in the patient or family 4. Tumor testing shows mismatch repair deficiency (dMMR) or microsatellite instability (MSI). Even if you don’t meet all the criteria, some doctors may recommend testing if the pathology shows specific features of your tumor.     What testing involves: 1. Genetic counseling first   A trained counselor reviews your personal and family history.  Explains implications for you, your relatives, and options for surveillance or preventive measures.  2. Testing   Blood or saliva sample for DNA analysis to look for mutations in MMR genes (MLH1, MSH2, MSH6, PMS2, EPCAM).  Tumor tissue testing may also be done to guide the decision.     Why it matters for you: Surveillance: If you have Lynch syndrome, your risk for other cancers is higher. You may need:   Colonoscopy starting earlier and more frequently  Possibly prophylactic removal of ovaries after childbearing  More frequent imaging or endometrial sampling Treatment decisions: Some therapies (like immunotherapy) work better for tumors with mismatch repair deficiency.     Why it matters for your family: Relatives may carry the same mutation. Early detection and preventive strategies can significantly reduce cancer risk. Children, siblings, and even more distant relatives may benefit from counseling and testing.     Bottom line: Not everyone with endometrial cancer needs testing, but if you are young, have high-risk features, or have a suspicious family history, genetic counseling is strongly recommended. It’s about planning for both your health and your family’s health.  Genetic testing does not change the fact that your tumor needs treatment, but it provides critical information for surveillance, prevention, and family planning.  If you want, I can make a simple decision guide showing who should get Lynch syndrome testing and what it would mean step by step for you and your relatives. | This is one of the most important long-term questions you can ask. The answer is: Yes, genetic counseling and testing are strongly recommended for a significant portion of endometrial cancer patients, and they have profound implications for both your treatment and your family's health.  Let's break down why, what it involves, and what it means.   Why Is It Recommended for Endometrial Cancer Patients?  Approximately 3–5% of all endometrial cancers are caused by an inherited genetic syndrome, the most common being Lynch syndrome (also known as Hereditary Non-Polyposis Colorectal Cancer or HNPCC). Other syndromes like Cowden syndrome (PTEN) are rarer.  You are more likely to be referred for genetic counseling if you: Are diagnosed under age 50. Have a personal history of another Lynch-related cancer (colorectal, ovarian, stomach, urinary tract, small bowel, pancreatic). Have a strong family history of these cancers, especially in multiple close relatives across generations. Have a tumor with specific pathological features (like mismatch repair deficiency, which can be seen on your initial pathology report).   What is Genetic Counseling and Testing?  This is a two-step process: 1. Genetic Counseling: A session with a certified genetic counselor. They will:  Review your personal and detailed family cancer history.  Explain the science, the specific syndromes (like Lynch), and what testing can and cannot tell you.  Discuss the potential emotional, insurance, and family implications.  Help you make an informed decision about whether to proceed with testing. 2. Genetic Testing: If you choose to proceed, it's usually a simple blood or saliva sample. The lab analyzes specific genes (MLH1, MSH2, MSH6, PMS2, EPCAM for Lynch) for harmful mutations.   What Does a Positive Result (for Lynch Syndrome) Mean for YOU?  1. Surgical Decisions:  Ovarian Cancer Risk: Lynch syndrome carriers have a 10–12% lifetime risk of ovarian cancer. This often makes the decision to remove the ovaries and fallopian tubes during your hysterectomy much clearer, even if you are young.  Extent of Surgery: Some may consider more extensive colon surgery at the same time if there's also a high colon cancer burden, but this is individualized.  2. Cancer Surveillance:  You will need enhanced, lifelong cancer screening. This most critically includes an annual colonoscopy starting at a young age (or immediately) to prevent colorectal cancer.  You may be offered screening for other Lynch-related cancers (urinary tract, stomach, small bowel).  3. Treatment Options:  Tumors with Lynch-related genetic changes (mismatch repair deficiency) may respond exceptionally well to a type of immunotherapy called PD-1/PD-L1 checkpoint inhibitors. This is relevant if you ever need treatment for advanced or recurrent disease.   What Does It Mean for YOUR FAMILY?  This is the most significant broader impact. Lynch syndrome is an autosomal dominant condition. Each of your first-degree relatives (parents, siblings, children) has a 50% chance of also carrying the same mutation. If you test positive, they can be offered targeted testing for the specific mutation you carry. This is called predictive testing. If they test positive, they can begin lifesaving surveillance (like early colonoscopies) to prevent cancers or catch them at the earliest, most curable stage. If they test negative, they can be relieved of the high-risk category and follow average population screening.   Summary: The "Why" Behind the Recommendation  Genetic testing is not just about a label; it's about actionable intelligence.  For You: It guides your surgeons and oncologists to provide the most personalized, risk-reducing care possible. For Your Family: It provides the opportunity for true cancer prevention, potentially saving lives across generations.  Your Next Steps: 1. Ask your gynecologic oncologist: "Based on my age and pathology, do you recommend I see a genetic counselor?" 2. If referred, Attend the counseling session with as much family health history as you can gather. 3. Take your time to decide about testing. The counselor will help you navigate the decision.  This proactive step transforms your diagnosis from a solitary event into a powerful tool for safeguarding your family's future health. It is one of the most impactful aspects of modern cancer care. |
| What should I pay attention to in terms of diet and nutrition before surgery? Do I need a blood transfusion or white blood cell boosters in advance? | Before endometrial cancer surgery, diet and nutrition are important to help your body handle anesthesia, heal efficiently, and reduce complications. Here’s what you should focus on:     1. General nutrition: High-protein foods: Eggs, lean meat, fish, tofu, beans, and dairy. Protein supports wound healing and immune function. Fruits and vegetables: Provide vitamins, minerals, and fiber to maintain gut health. Whole grains: Brown rice, oats, whole-wheat bread for energy and stable blood sugar. Hydration: Drink enough water unless your doctor restricts fluids before surgery.     2. Specific considerations: Iron-rich foods: Red meat, spinach, lentils, or iron-fortified cereals if you are mildly anemic. This helps reduce the risk of needing a blood transfusion. Avoid excessive fat or sugar: Can worsen blood sugar control, especially if you have diabetes. Small, frequent meals: Can help stabilize blood sugar and prevent nausea. Supplements: Only take vitamins/minerals if approved by your doctor; some (like high-dose vitamin E, fish oil, or herbal supplements) can increase the risk of bleeding.     3. Preoperative fasting: Typically, nothing by mouth 6–8 hours before surgery. Clear liquids may be allowed up to 2 hours before anesthesia. Follow your anesthesiologist’s instructions carefully to prevent aspiration during surgery.     4. Blood transfusions and white blood cell boosters. Routine pre-surgery transfusions are usually unnecessary unless you have significant anemia. White blood cell boosters (like G-CSF) are generally not given prophylactically before surgery; they are used after chemotherapy if counts drop. Your blood counts will be checked before surgery. If they are low, your doctor may treat the deficiency, but this is individualized.     Bottom line  1. Eat a balanced, protein-rich diet and stay hydrated. 2. Correct anemia or nutrient deficiencies if present. 3. Follow pre-op fasting instructions precisely. 4. Blood transfusions or immune boosters are only given if lab tests indicate a need, not routinely.  Good nutrition before surgery is mostly about optimizing your body’s resilience so recovery is faster and complications are minimized.  I can also give a sample pre-surgery diet plan for 1–2 weeks to prepare your body if you want. | These are excellent and practical questions. Proper preoperative preparation can optimize your body's ability to handle surgery and heal. Let's address each part clearly.   Part 1: Diet & Nutrition Before Surgery  The goal is to enter surgery well-nourished and hydrated, not to start any drastic new regimens.  What TO DO: Focus on Protein: Protein is the building block for tissue repair and immune function. Include lean meats, fish, eggs, beans, tofu, and Greek yogurt. Eat a Balanced, Anti-inflammatory Diet: Plenty of fruits, vegetables, and whole grains. These provide essential vitamins (like Vitamin C for collagen formation) and minerals. Stay Hydrated: Drink plenty of water in the days leading up to surgery. Well-hydrated veins make IV placement easier. Manage Constipation: Surgery, anesthesia, and pain medications can severely slow your bowels. If you are prone to constipation, increasing fiber intake *gradually ahead of time and staying hydrated can help set you up for a better post-op course. Discuss a bowel regimen with your doctor.  What TO AVOID: Extreme Diets or Fasting: Do not start a severe calorie-restricted diet. Your body needs energy to heal. "Blood-Thinning" Supplements: CRITICAL – You must discontinue supplements that can increase the risk of bleeding. These include Vitamin E, fish oil, omega-3s, garlic, ginkgo biloba, ginseng, and high-dose turmeric/curcumin. Inform your surgeon of all supplements; a common rule is to stop them 1–2 weeks before surgery. Alcohol: Avoid alcohol for at least 48 hours before surgery, as it can affect liver function and interact with anesthesia. Overeating or Heavy, Greasy Meals: The day before surgery, eat light, easily digestible meals.  The Golden Rule: Pre-Surgical Fasting: You will receive strict instructions from your surgical team, typically: NO solid food after midnight the night before your surgery. Clear liquids (water, black coffee, apple juice) may be allowed up to 2–4 hours before your arrival time. You must follow the specific instructions given to you to avoid cancellation.   Part 2: Blood Transfusion & White Blood Cell Boosters  The short answer is: No, these are almost never needed or given *in advance of routine endometrial cancer surgery.  Let's break down why:  1. Blood Transfusion (Packed Red Blood Cells): Purpose: To replace blood volume and carry oxygen after significant blood loss. Pre-Op Use: Extremely rare. It is only considered if you have severe, symptomatic anemia (very low red blood cell count) discovered during pre-op testing that would make anesthesia dangerous. The goal would be to correct it *before surgery, not to "boost" levels arbitrarily. Typical Scenario: Blood loss during a hysterectomy is usually modest. The surgical team is prepared to give a transfusion during or after surgery only if needed due to unexpected bleeding. You will be typed and cross-matched (blood drawn for compatibility) pre-op as a precaution, not because you expect to need it.  2. White Blood Cell Boosters (Growth Factors like Neulasta/Filgrastim): Purpose: To stimulate the bone marrow to produce more infection-fighting white blood cells (neutrophils). This is a standard part of chemotherapy regimens, which can suppress the bone marrow. Pre-Op Use: Virtually never. Surgery itself does not cause a dangerous drop in white blood cells that requires pre-treatment. Your white count may rise slightly after surgery as a normal stress/inflammatory response. These drugs have significant side effects (bone pain) and are only used in the context of chemotherapy, not before standalone surgery.   Your Pre-Op Checklist & Questions  1. Review All Meds & Supplements: With your surgeon, create a clear list of what to take and what to stop before surgery. 2. Ask About Your Blood Counts: At your pre-op appointment, you can ask: "What were my hemoglobin and hematocrit levels? Am I anemic, and does it need to be addressed before surgery?" 3. Follow fasting instructions precisely. 4. Focus on Overall Health: The best preparation is good nutrition, hydration, and, if possible, light activity (like walking) to maintain fitness.  Summary: Your surgical team will optimize your medical conditions (like anemia) if necessary. You do not need prophylactic "boosters." Your role is to provide your body with good foundational nutrition while avoiding anything that increases surgical risk, like certain supplements. Always defer to the specific written instructions provided by your hospital's preoperative clinic. |
| I am taking aspirin/anticoagulants/supplements. How long before surgery should I stop them? | This is a very important question because blood-thinning medications can significantly increase the risk of bleeding during and after surgery. Timing depends on the specific drug, dose, and why you’re taking it. Here’s a practical guide:     1. Aspirin (low-dose, e.g., 81 mg daily)  Often used for heart protection. Typical recommendation: Stop 5–7 days before surgery to allow platelets to function normally. Exception: If you have a high-risk heart condition (stent, recent heart attack), your cardiologist may advise continuing aspirin, weighing bleeding vs. cardiac risk.     2. Other anticoagulants  \| Drug type \| Typical stop time before surgery \| Notes \| \| \| -- \| \| \| Warfarin \| 4–5 days \| May require bridging with short-acting heparin depending on clot risk \| \| DOACs (apixaban, rivaroxaban, dabigatran, rivaroxaban) \| 24–48 hours for normal kidney function \| Longer if kidney function impaired \| \| Heparin (subcutaneous for prophylaxis) \| Usually skip the dose the night before \| Check with doctor \|  Key: Always coordinate with both your surgeon and prescribing physician, because stopping anticoagulants can increase your risk of clots, while continuing them increases bleeding.     3. Supplements and herbal products: Some can increase bleeding risk and should generally be stopped 1–2 weeks before surgery:  Fish oil/omega-3 at high doses Vitamin E Garlic, ginger, ginkgo, ginseng, St. John’s wort, some weight-loss/herbal teas  Tip: Bring a complete list of everything you take to the pre-op visit. The surgical/anesthesia team will decide what to stop and when.     4. General rules: 1. Do not stop prescription medications without guidance from the prescribing doctor. 2. Timing depends on the drug's half-life, kidney function, and clot risk. 3. Always inform the anesthesiologist about any medications you take on the day of surgery.     Bottom line: Aspirin: usually stop 5–7 days before Warfarin: stop 4–5 days; may need bridging DOACs: stop 1–2 days before (longer if kidney issues) Herbs/supplements: typically stop 1–2 weeks before  Proper management balances bleeding risk vs. clot risk, so coordination among your surgeon, anesthesiologist, and prescribing doctor is essential.  I can also make a timeline table showing exactly when to stop common medications and supplements before surgery so it’s easy to follow. Do you want me to do that? | This is one of the most critical pre-operative questions. Managing these medications incorrectly can lead to severe bleeding during surgery or dangerous blood clots if stopped unnecessarily. The following are GENERAL guidelines, but you MUST get and follow SPECIFIC, WRITTEN instructions from your surgical team.  The answer depends entirely on why you are taking the medication and the specific drug. Here is a breakdown by category.     1. Prescription Anticoagulants ("Blood Thinners") DO NOT STOP THESE ON YOUR OWN. Stopping them without a plan can cause life-threatening clots (e.g., stroke, heart attack, pulmonary embolism).  Warfarin (Coumadin): Typically stopped 5 days before surgery. You will likely need to switch to a short-acting "bridge" therapy (like injectable heparin) and stop that closer to surgery. This requires close coordination between your surgeon, cardiologist, and hematologist. Direct Oral Anticoagulants (DOACs): (e.g.,, apixaban (Eliquis), rivaroxaban (Xarelto), dabigatran (Pradaxa), edoxaban (Savaysa)).  These are usually stopped 24–48 hours before surgery, depending on kidney function and bleeding risk. Clopidogrel (Plavix), Ticagrelor (Brilinta), and Prasugrel (Effient):  These are potent antiplatelet drugs often taken after heart stents. Stopping them carries a high risk of stent thrombosis. Stopping requires explicit, joint decision-making between your surgeon and cardiologist. It may be stopped 5–7 days prior, but this is highly individualized.   2. Aspirin & NSAIDs (Non-Steroidal Anti-Inflammatory Drugs) Low-Dose Aspirin (81 mg or 100 mg) for heart/stroke prevention:  Many surgeons now advise CONTINUING it for low-bleeding-risk surgeries if the cardiac benefit outweighs the risk. You must ask. If instructed to stop, it's typically 5-7 days before surgery. Higher-dose aspirin (325 mg) or NSAIDs for pain (e.g., ibuprofen), ibuprofen, naproxen, diclofenac):  These are typically stopped 5–7 days before surgery due to bleeding risk.   3. Dietary & Herbal Supplements Many patients forget these. But they can significantly increase the bleeding risk. A general rule is to stop all supplements 1–2 weeks before surgery unless specifically approved by your surgeon.  High-Risk Supplements to STOP (Common Examples): Fish Oil / Omega-3s, Vitamin E, Garlic, Ginkgo Biloba, Ginseng, Turmeric/Curcumin (in high doses), St. John's Wort (can interact with anesthesia), and Green Tea Extract   Your Action Plan: A Non-Negotiable Checklist  1. Create a Complete List: Write down every single medication and supplement you take, including the dose and frequency. 2. Disclose the Reason: For each prescription, note why you take it (e.g., "Eliquis for atrial fibrillation," "Plavix for a heart stent"). 3. Get WRITTEN instructions: At your preoperative appointment, the surgeon or anesthesiologist will give you a personalized written schedule of what to stop and when. If you don't receive one, ask for it. 4. Verify at the Pre-Anesthesia Visit: This is your final safety check. Bring your list and confirm the plan. 5. Morning of Surgery: You will be told which pills, if any, to take with a tiny sip of water (e.g., heart or blood pressure medications).   Critical Summary: Never self-manage this process. The risk of bleeding or clotting is too high. The timing depends on the drug, your health condition, and the specific surgery. Your final, authoritative instructions will come from your surgical/anesthesia team.  The single most important thing you can do is provide your surgical team with a complete list and follow their precise orders. This is a standard part of their pre-op protocol, and they are experts in managing it safely. |
| How can postoperative thrombosis (deep vein thrombosis) be prevented? What preparations are needed before surgery? | Preventing postoperative thrombosis (DVT/PE) is a major focus in endometrial cancer surgery, especially because pelvic surgery, cancer itself, and reduced mobility all increase clot risk. Prevention starts before, during, and after surgery. Here’s a practical guide:     1. Preoperative preparation  a. Risk assessment: Your doctor evaluates your personal and family history of blood clots, age, obesity, smoking, hormone therapy, and other comorbidities. High-risk patients may require more aggressive prevention.  b. Medication management: Some patients are started on low-molecular-weight heparin (LMWH) or other anticoagulants before surgery if the risk is high. Timing is carefully planned to minimize bleeding risk during surgery.  c. Hydration  Good hydration before surgery keeps blood viscosity lower and reduces clot risk.  d. Mobility planning: Surgeons plan early post-op mobilization, sometimes even the evening of surgery.     2. Intraoperative measures: a. Mechanical prophylaxis. Intermittent pneumatic compression devices (inflatable leg sleeves) may be placed during surgery. Compression stockings may also be used. Both help keep blood flowing in the legs while you’re immobile.  b. Minimally invasive surgery: Laparoscopy or robotic surgery reduces tissue trauma and allows faster recovery and earlier ambulation, lowering clot risk compared to open surgery.     3. Postoperative prevention: a. Early mobilization. Walking as soon as your doctor allows (usually within 12–24 hours for minimally invasive surgery). Even small leg exercises in bed help.  b. Medications  Prophylactic anticoagulants (e.g., LMWH) may be continued for several days post-op, sometimes up to 4 weeks if you are high-risk.  c. Mechanical devices  Compression stockings or intermittent pneumatic devices may continue post-op until you are mobile.  d. Lifestyle measures: Avoid prolonged sitting in the first weeks after surgery. Maintain hydration. Report any leg swelling, pain, redness, or shortness of breath immediately.     4. Monitoring  Your care team may check blood counts and coagulation tests before starting or stopping anticoagulation. High-risk patients may have an ultrasound of the legs if suspicion arises.     Bottom line: Prevention is multi-layered:  1. Before surgery: assess risk, optimize hydration, and plan anticoagulants if needed 2. During surgery: use compression devices, and choose a minimally invasive approach if possible. 3. After surgery: early ambulation, prophylactic anticoagulants, and continued mechanical compression. Most young, healthy patients with minimally invasive surgery need only short-term medication plus early walking, while higher-risk patients may need extended prophylaxis.  If you want, I can make a step-by-step DVT prevention timeline before and after surgery, showing exactly what happens and when. | This is an exceptionally important question. Postoperative Deep Vein Thrombosis (DVT) and its potentially fatal complication, Pulmonary Embolism (PE), are significant risks after major surgery like a hysterectomy. Preventing them is a top priority for your surgical team, and it involves a multi-layered strategy that starts before you even enter the operating room.  Here is a comprehensive breakdown of how it’s prevented and what preparations are needed.   Why Is the Risk High (Virchow's Triad)? Surgery creates a "perfect storm" for clot formation: 1. Blood Stasis: Immobility during and after surgery slows blood flow in the legs. 2. Vessel Injury: The surgical procedure itself can damage blood vessel walls. 3. Hypercoagulability: Your body's natural response to surgery and cancer is to increase blood clotting.   The Multi-Layered Prevention Strategy ("Thromboprophylaxis")  # 1. Pre-Operative Preparations & Assessment: This is the foundation. Your team will: Perform a Risk Assessment: They will calculate your personal risk using tools like the Caprini Score, which factors in your age, cancer diagnosis, type/length of surgery, BMI, and personal/family history of clots. Review All Medications: As discussed, they will manage blood thinners. They will also ask about:  Hormone Therapy: Any estrogen-containing medications (HRT, birth control pills) must be stopped 3–4 weeks before surgery. Encourage prehabilitation: Being more active before surgery improves overall circulation and resilience.  # 2. Intra-Operative (During Surgery) Measures Sequential Compression Devices (SCDs): These are inflatable sleeves placed on your calves before anesthesia. They rhythmically squeeze and release, mimicking muscle contractions to keep blood flowing. This is standard for almost every patient. Early Anesthesia & Surgical Techniques: Minimally invasive surgery (laparoscopy/robotics) itself reduces risk compared to open surgery. The anesthesiologist carefully manages your fluid balance.  # 3. Post-Operative (After Surgery) Protocol – THE MOST CRITICAL PHASE SCDs Continued: You will wear these constantly while in bed. Early and Frequent Ambulation: This is the single most effective thing you can do. You will be helped to sit up, stand, and walk within hours of surgery, even if just a few steps. This is non-negotiable for prevention. Pharmacologic Prophylaxis (Blood-Thinning Injections):  For most cancer patients, this is standard. You will receive a daily subcutaneous injection of a low-dose anticoagulant (e.g., enoxaparin/Lovenox, heparin) starting 6–24 hours after surgery.  These injections continue throughout your hospital stay and, for high-risk patients, may be prescribed for 2–4 weeks after discharge. Hydration and Leg Exercises: You will be encouraged to drink fluids and perform ankle pump exercises (flexing and pointing toes) regularly while in bed.   Your Role & Key Questions to Ask: You are an active partner in this prevention plan.  Before Surgery: Disclose a Full History: Tell your team about any personal or family history of blood clots, clotting disorders, or miscarriages. Ask: "Based on my risk factors, will I need blood-thinning injections after surgery, and for how long after I go home?"  After Surgery: Move, Move, Move: Adhere strictly to the ambulation schedule. Do your ankle pumps every hour when awake. Know the Symptoms: Report these immediately:  DVT: New, persistent calf or thigh pain, swelling, redness, or warmth in one leg.  PE: Sudden shortness of breath, sharp chest pain (worse with deep breathing), coughing (especially if bloody), and a rapid heart rate. Feeling faint. Comply with Medications: If prescribed injections to take at home, ensure you understand how and when to administer them.   Special Considerations for Endometrial Cancer Patients: Cancer itself is a major risk factor, often requiring extended prophylaxis. Obesity is a common comorbidity that further increases risk, making mechanical (SCDs) and pharmacologic (injections) measures even more essential. Minimally invasive surgery reduces, but does not eliminate, the risk. Prophylaxis is still mandatory.   Summary of Preventative Measures \| Timing \| Measure \| Purpose \| \| : \| : \| : \| \| Before \| Risk assessment, stop hormones \| Identify and mitigate personal risk. \| \| During \| Sequential Compression Devices (SCDs) \| Mechanically pump blood in the legs while immobilized. \| \| After \| 1. Early Ambulation<br>2. SCDs (while in bed)<br>3. Blood-thinning Injections<br>4. Hydration & Leg Exercises \| A multi-faceted attack on clot formation. \|  Rest assured, DVT prevention is a routine, protocol-driven part of your care. By understanding the plan and actively participating—especially by walking early and often—you significantly contribute to your own safe recovery. |
| My anemia is severe. Will it affect the surgery? Does it need to be corrected first? | Yes—severe anemia is an important issue before surgery. It can significantly affect both your safety during the operation and your recovery afterward. Here’s the breakdown:     1. How anemia affects surgery: Reduced oxygen delivery: Your organs, especially the heart, brain, and surgical site, get less oxygen. This can increase the risk of complications. Higher risk of blood transfusion: If your hemoglobin is very low, even normal blood loss during surgery can drop it to unsafe levels. Delayed wound healing: Anemia can slow tissue repair and increase the infection risk. Fatigue and recovery: You may feel weaker and recover more slowly postoperatively.     2. Preoperative correction. Whether and how anemia is corrected depends on the cause, severity, and time available before surgery.  Common strategies:  1. Iron deficiency anemia   Oral iron supplements if there’s time (weeks) before surgery.  Intravenous iron if oral therapy is too slow or poorly tolerated.  2. Anemia from chronic disease or other causes   May require erythropoiesis-stimulating agents (like EPO) in select patients.  Treat the underlying cause if possible.  3. Blood transfusion   Considered if hemoglobin is very low or surgery is urgent.  Transfusion raises hemoglobin quickly and reduces perioperative risk.     3. Timing: Ideally, hemoglobin should be optimized before elective surgery. Mild anemia may be acceptable, but severe anemia usually needs correction. Your surgeon and anesthesiologist will weigh the urgency of cancer surgery vs. correction time.     4. Practical tips: Take all prescribed iron or supplements exactly as directed. Eat iron-rich foods (lean meat, beans, dark leafy greens) plus vitamin C for absorption. Monitor hemoglobin closely in the weeks before surgery. Communicate any fatigue, dizziness, or shortness of breath to your doctor—they may indicate urgent correction is needed.     Bottom line: Severe anemia can increase surgical risk and delay recovery, so it usually needs to be corrected before surgery if time allows. How it is corrected depends on the cause, severity, and how urgently the surgery needs to happen.  If you want, I can outline a pre-surgery anemia optimization plan showing what labs, treatments, and timing are typically used to safely prepare for surgery. | This is a vital concern. The short answer is: Yes, severe anemia can significantly affect the safety of your surgery, and yes, it usually needs to be identified and corrected beforehand. This is a standard and essential part of preoperative optimization.  Here is a detailed breakdown of the risks, the goals, and how this is managed.   How Severe Anemia Affects Surgery and Recovery  Your red blood cells (hemoglobin) carry oxygen to all your tissues. During the stress of surgery and healing, your body's oxygen demand increases. Severe anemia compromises this system, leading to:  1. Increased Cardiac Strain: Your heart must work much harder to deliver the same amount of oxygen, raising the risk of heart attack, arrhythmias, or heart failure, especially under anesthesia. 2. Poor Tolerance of Blood Loss: Even normal surgical blood loss can become dangerous if your starting volume is already low. 3. Impaired Wound Healing and Infection Risk: Oxygen is crucial for tissue repair and immune function. Anemia can lead to poorer healing and higher infection rates. 4. Prolonged Fatigue and Recovery: You may feel excessively weak and fatigued post-op, hindering your ability to walk and perform necessary activities for recovery.   Does It Need to Be Corrected First? Almost certainly, yes.  The goal is to minimize the above risks. Your surgical and anesthesia team will have a specific hemoglobin target they want you to meet before proceeding. This is often a hemoglobin (Hgb) level > 8.0 g/dL, and ideally closer to 10.0 g/dL for major surgery.  The crucial step is to determine the CAUSE of your anemia. The treatment depends entirely on the cause:  Iron-Deficiency Anemia (Most Common): Very common in women, especially with heavy menstrual bleeding, which is a frequent symptom of endometrial cancer.  Treatment: Oral or intravenous (IV) iron supplementation. IV iron is often preferred pre-operatively because it repletes iron stores much faster (within 1–2 weeks) than oral iron (which can take months and often causes GI side effects). Anemia of Chronic Disease/Inflammation: The cancer itself can cause anemia.  Treatment: Addressing the cancer is the primary treatment, but IV iron or other medications (like erythropoiesis-stimulating agents - ESAs) may be used cautiously in coordination with your oncologist. Other Nutritional Deficiencies: (e.g., B12 or folate).  Treatment: Specific vitamin replacement. Other Causes: Such as bone marrow issues or kidney disease, requiring specialist management.   Will This Delay My Cancer Surgery?  This is a careful balance. The short delay required to correct anemia (often 1–3 weeks) is almost always justified by the significant reduction in surgical risk. Rushing into surgery with severe anemia is more dangerous than a brief, purposeful delay.  Your team's priority is: "First, do no harm." Optimizing your condition is the first step to a successful surgery.   What About a Blood Transfusion Before Surgery?  A pre-operative blood transfusion is generally considered only if: The anemia is very severe and symptomatic (e.g., Hgb < 7.0–8.0 g/dL with dizziness and shortness of breath at rest). The surgery is urgent and cannot wait for iron therapy to work (less common for planned cancer surgery). Other methods of correction are ineffective or contraindicated.  Transfusions carry their own small risks (reaction, infection, immune sensitization), so the team will try to correct anemia with iron therapy first if time allows.   Your Action Plan: Key Questions to Ask  1. Get the Numbers: Ask, "What is my exact hemoglobin and ferritin (iron store) level?" 2. Understand the Cause: Ask, "What is the most likely cause of my anemia?" 3. Know the Plan: Ask, "What is the recommended treatment to correct it before surgery?" (e.g., IV iron?) How long will that take, and what is our target hemoglobin?" 4. Discuss Timeline: "Will this change my scheduled surgery date, and if so, by approximately how long?"   Summary Severe anemia increases surgical risk and must be addressed. Correction is typically required before surgery to ensure your safety. An IV iron infusion is a common, effective, and fast solution for iron-deficiency anemia. This process is a standard part of preoperative care. Your medical team is experienced in managing it efficiently.  Your role is to report any symptoms (extreme fatigue, dizziness, shortness of breath) and to comply with the treatment plan (e.g., receiving IV iron). By proactively managing your anemia, you are actively participating in making your surgery as safe and successful as possible. |
| Is bowel preparation required before surgery? How is it done? | For endometrial cancer surgery, routine full bowel preparation is usually *not required, especially for minimally invasive procedures like laparoscopic or robotic hysterectomy. The approach depends on the type of surgery and whether bowel involvement is expected. Here’s a breakdown:     1. When bowel prep might be used: If the surgeon expects bowel surgery or resection (rare in early-stage endometrial cancer) If the patient has constipation, prior bowel surgery, or obstruction Some hospitals still use light prep for anesthesia and intra-abdominal visualization     2. Types of bowel preparation: a. Mechanical prep: Laxatives or oral solutions (like polyethylene glycol) to clear stool from the colon. Usually taken the night before surgery. b. Enemas: Sometimes used for local cleansing of the rectum or sigmoid colon. Less common for gynecologic surgery. c. Dietary prep: A clear liquid diet for 12–24 hours before surgery (water, broth, tea, clear juices). Avoid red/purple liquids if your surgeon will need to visualize the abdomen clearly.     3. Why bowel prep is limited in endometrial surgery: Minimally invasive surgery rarely involves bowel resection, so an empty colon isn’t critical. Studies show routine prep does not reduce surgical complications or infections in these cases. Over-preparation can cause dehydration or electrolyte imbalances, which actually increase the risk.     4. Practical tips: Follow your surgeon’s instructions: some centers may recommend light prep or clear liquids, others none. Drink plenty of fluids if you do prep to avoid dehydration. Report any severe constipation, diarrhea, or abdominal pain before surgery.     Bottom line  Routine bowel prep is not usually necessary for standard endometrial cancer surgery. Prep is individualized: only used if bowel involvement is likely, for comfort, or per hospital protocol. Focus on hydration, clear liquids if instructed, and following the surgeon's directions.  I can also make a sample 24-hour pre-surgery nutrition and bowel prep schedule to show exactly what to eat/drink before your operation. | This is a very practical and common question. The answer is: It depends entirely on your surgeon's specific protocol. There is variation in practice, but here is what you need to know.   Is Bowel Preparation Required?  For a standard total hysterectomy for endometrial cancer, full mechanical bowel prep (like for colon surgery) is typically NOT required.  However, some form of bowel "preparation" or "regimen" is almost always recommended. This usually means a gentle clean-out rather than a rigorous purge. The goals are different.  Common Surgeon Preferences: No Formal Bowel Prep: Many surgeons believe a clear liquid diet and a mild laxative are sufficient. "Mini-Prep": A gentle regimen to empty the rectum and sigmoid colon to improve surgical field visualization, especially for minimally invasive surgery. Full Bowel Prep: May be required if there is suspicion of bowel involvement from advanced cancer, or if the surgeon anticipates a possibility of bowel resection.  You will receive explicit, written instructions from your surgical team. Do not assume; always follow their specific orders.     What is the typical purpose of a "bowel regimen"?  1. To Empty the Lower Bowel: Makes it easier to retract the intestines during surgery, giving the surgeon a better view and more room to work, especially in the deep pelvis. 2. To Reduce Post-Op Constipation and Gas Pain: Anesthesia and pain medications severely slow gut function. Starting with an empty colon can make the initial post-op period more comfortable and reduce bloating. 3. To Minimize Infection Risk: In the rare event of an unrecognized bowel injury during surgery, having an empty, clean bowel reduces the risk of severe infection.     How Is It Usually Done? (Common Protocol Examples)  If instructed, a typical "mini-prep" might look like this:  The Day Before Surgery: Diet: You will be placed on a clear liquid diet (broth, apple juice, gelatin, clear sports drinks, black coffee/tea). No solid foods. Laxatives: You may be instructed to take one or more of the following in the afternoon/evening:  Bisacodyl (Dulcolax) tablets: A stimulant laxative.  Magnesium Citrate: A saline laxative liquid.  A phosphate enema or a bisacodyl suppository: To empty the very distal rectum.  The Night Before / Morning of Surgery: Fasting: You will begin a strict NPO (nothing by mouth) period, typically after midnight, except for sips of water with approved medications.   Crucial Points & Precautions  1. Hydration is Key: When on a clear liquid diet, drink plenty of allowed fluids to stay well-hydrated. Dehydration before surgery is dangerous. 2. Medication Adjustments: Your instructions will specify which medications to take or hold. This is especially important for diabetes medications and blood thinners. 3. Follow Instructions Precisely: Deviating from the prep (e.g., eating solid food) can lead to surgery being cancelled for your safety. 4. Plan to Be Near a Bathroom: Once the laxatives/enema take effect, you will need immediate access to a toilet.   What to Ask Your Surgeon  To eliminate confusion, ask at your pre-op appointment: "Do you require a specific bowel preparation before my surgery?" "If yes, can you provide me with the written, step-by-step instructions?" (They often give a printed handout). "What is the goal of the prep in my specific case?"   Summary While a full "colonoscopy-style" bowel prep is uncommon, a gentle bowel regimen involving a clear liquid diet and mild laxatives is frequently used to optimize conditions for surgery and your post-op comfort. Your surgical team's specific, written instructions are the final and only authority. Following them exactly is a critical step in preparing for a safe and successful operation. |
| What psychological and practical preparations should I make before surgery? | Facing endometrial cancer surgery can be stressful, so psychological and practical preparation are just as important as physical preparation. Both reduce complications and improve recovery. Here’s a clear, structured approach:     1. Psychological preparation  a. Understand the procedure. Learn what will happen in surgery: which organs will be removed, whether lymph nodes will be sampled, anesthesia, and recovery expectations. Ask your doctor about risks, benefits, and alternatives. Understanding reduces fear of the unknown.  b. Set realistic expectations: Recovery may take weeks; fatigue, pain, and temporary limitations are normal. Emotional ups and downs are common; acknowledging them helps you cope.  c. Stress management techniques: Breathing exercises or meditation: 5–10 minutes a day can reduce anxiety. Mindfulness or guided imagery: Visualizing smooth surgery and recovery can improve confidence. Talk to someone: Family, friends, or a counselor/therapist experienced in cancer care.  d. Prepare for body image and fertility changes  If hysterectomy or ovary removal is planned, some emotional adjustment may be needed. Consider discussing fertility preservation, sexual health, and hormonal changes with your doctor beforehand.     2. Practical preparations: a. Home preparation: Arrange a comfortable recovery area with easy access to the bathroom, water, snacks, and entertainment. Plan for help with meals, errands, and chores for at least 1–2 weeks post-op. Remove hazards (loose rugs, clutter) to prevent falls during early mobility.  b. Transportation  Arrange for someone to drive you to and from the hospital, especially if minimally invasive surgery is done as an outpatient or short-stay.  c. Medication management: Organize all prescription meds, supplements, and dosing schedule. Make a list for the hospital staff and anesthesiologist.  d. Clothing and personal items: Loose, comfortable clothes for post-op wear. Easy-to-wear shoes for walking soon after surgery. Toiletries, a phone charger, and any medical devices you use.  e. Postoperative planning: Stock up on easy-to-prepare meals, snacks, water, and high-protein foods. Have a list of emergency contacts and your surgeon’s office number handy. Plan short walks or light activity in your home to encourage circulation and reduce the risk of DVT.     3. Education and support: Consider joining a patient support group for gynecologic cancer. Ask your surgeon or nurse for pre-op educational materials—they often provide checklists and videos. Understanding what pain, drains, or catheters might feel like reduces anxiety and improves cooperation with recovery instructions.     Bottom line: Psychological prep: understand the procedure, manage stress, and anticipate emotional changes. Practical prep: organize the home, transportation, medication, food, and mobility aids. Combining both improves safety, recovery speed, and mental well-being after surgery.  I can also make a detailed pre-surgery checklist combining psychological, nutritional, and practical steps day by day for the week before surgery if you want—it makes preparation very concrete. | Preparing for major surgery involves both your mind and your environment. A well-considered plan can significantly reduce anxiety and pave the way for a smoother recovery. Here is a comprehensive guide to psychological and practical preparations.   Psychological & Emotional Preparation  This is about building your mental resilience and support system.  1. Educate Yourself (But Set Limits): You've done excellently by asking detailed questions. Understanding the "what" and "why" reduces fear of the unknown. However, avoid late-night internet deep dives into worst-case scenarios. 2. Acknowledge Your Feelings: It's normal to feel anxiety, fear, sadness, or even anger. Allow yourself to feel these emotions without judgment. Talk about them with a trusted friend, family member, or counselor. 3. Practice Stress-Reduction Techniques:  Mindfulness & Breathing: Apps like Calm or Headspace have short guided meditations for pre-surgery anxiety. Simple deep-breathing exercises (the 4-7-8 technique) can calm the nervous system.  Visualization: Spend a few minutes daily visualizing a positive outcome—seeing yourself in recovery, moving comfortably, and healing well. 4. Build Your Support Team:  Identify your primary caregiver(s) for the first week home.  Communicate Your Needs: Be specific with your support system. Tell them if you need someone to just listen, help with chores, or distract you.  Consider Professional Support: Ask your hospital about oncology social workers or psychologists. A single session can provide powerful coping tools. 5. Set Intentions, Not Just Expectations: Instead of fixating on a perfect recovery timeline, set gentle intentions: "I will listen to my body," "I will accept help," "I will be patient with the process."   Practical & Logistical Preparation: Getting your life and home ready is an active way to regain a sense of control.  1. At Home: Create a "Recovery Zone": Set up a comfortable chair or bed area with pillows, a side table for water, medications, a phone charger, books/remote, and a reachable basket with essentials. Meal Prep: Cook and freeze 2–3 weeks' worth of meals in individual portions. Stock up on easy-to-digest foods (soup, crackers, yogurt, protein shakes). Home Safety:  Remove tripping hazards (loose rugs, cords).  Place frequently used items (cups, plates, clothes) at waist level to avoid bending/reaching.  Consider a toilet seat riser and a shower chair/grip bars—these are invaluable for safety and independence.  Ensure you have loose, comfortable clothing (front-opening shirts, elastic-waist pants, slip-on shoes).  2. Logistics & Paperwork: Work & Finances: Complete necessary medical leave paperwork. Set up online bill payments for the next month. Delegate: Assign a trusted person to handle mail, pet care, water plants, and take out trash. Transportation: Schedule rides to and from the hospital. Plan for follow-up doctor appointments.  3. Hospital Bag Essentials: Comfort Items: Lip balm, lotion, earplugs, eye mask, hard candy (for dry mouth). Entertainment: Phone/tablet with a long charger, book, and podcast playlist. Practical Clothing: Going-home outfit (very loose dress or pajamas), non-slip socks/slippers, and a warm robe. Documents: ID, insurance card, list of medications/allergies, and your surgeon's contact information.   Immediate Pre-Surgery Checklist (The Day Before) Follow all dietary and bowel prep instructions precisely. Shower with antibacterial soap if instructed. Confirm arrival time and transportation. Remove all jewelry, nail polish, and piercings. Get a good night's sleep. If anxious, ask your doctor for a safe sleep aid.   A Final Note on the Day of Trust the team you have carefully chosen. You have done the work to be prepared. Once you check in, your job is to surrender to the process and the care of your medical team. They will guide you the rest of the way.  By addressing both the emotional and tangible aspects of preparation, you build a strong foundation for healing. This proactive approach is a powerful step in your journey through treatment and recovery. You've got this. |
| III. Postoperative Care and Complication Management |  |  |
| How is postoperative pain managed? How long can a patient-controlled analgesia pump be used? | Postoperative pain management after endometrial cancer surgery is multimodal, meaning it uses several strategies to keep you comfortable while minimizing side effects. Here’s the breakdown:     1. Types of pain management: a. Patient-Controlled Analgesia (PCA) — Usually an IV pump that allows you to self-administer small doses of pain medication (commonly opioids like morphine or hydromorphone). How it works: You press a button when you feel pain; the pump gives a preset dose with a lockout interval to prevent overdose. Duration: Typically 24–72 hours, depending on your pain level and recovery. Doctors reassess daily. Often combined with other methods to reduce opioid needs.  b. Oral or IV medications: Acetaminophen/paracetamol or NSAIDs (ibuprofen, celecoxib) are used for mild to moderate pain. These may be continued after PCA is stopped.  c. Local anesthetics. Long-acting local anesthetics at incision sites or laparoscopic port sites can reduce early postoperative pain. Some centers use nerve blocks or epidural analgesia in open or extensive surgery.  d. Non-drug methods: Ice packs or heat (for minor discomfort), relaxation techniques, and breathing exercises. Early mobilization: walking reduces stiffness and abdominal discomfort.     2. Typical pain timeline: First 24 hours: Pain is usually moderate; PCA or IV medications are most effective. Days 2–3: Pain often decreases; PCA may be discontinued, switching to oral painkillers. After 1 week: Mild incision or abdominal discomfort may persist but is usually manageable with over-the-counter medications.     3. Monitoring and safety: Nurses and doctors monitor for side effects of opioids: nausea, itching, constipation, drowsiness, or respiratory depression. Pain scores are regularly assessed to adjust medications. PCA allows personalized pain control, which can improve comfort and recovery.     4. Bottom line: Pain is managed proactively and in layers, not just with a single drug. PCA is usually used for 1–3 days, then patients transition to oral medications. Early pain control helps you mobilize sooner, breathe deeply, and recover faster, which also reduces complications like blood clots or pneumonia.  If you want, I can make a sample pain management plan for the first week after surgery, showing what medications and techniques are used on each day. | Excellent questions. Effective pain management is a cornerstone of recovery, allowing you to rest, breathe deeply, and begin moving—all of which are crucial for healing and preventing complications.  Here’s a detailed look at modern postoperative pain management, with a specific focus on PCA pumps.   How is Postoperative Pain Managed? (The Multi-Modal Approach)  The goal is not to eliminate all sensation, but to control pain to a manageable level with the fewest side effects. Today, the standard is "multimodal analgesia"—using different types of medications that work through different pathways. This approach provides better pain control while minimizing the doses (and side effects) of any single drug, particularly opioids.  1. The Foundation (Non-Opioids): Given routinely. Acetaminophen (Tylenol): Administered intravenously (IV) during/after surgery, then orally. Reduces pain and fever. NSAIDs (e.g.,, Ibuprofen, Ketorolac): IV or oral. Powerful anti-inflammatory drugs that target surgical site inflammation and pain. A key component for reducing opioid needs.  2. Local & Regional Anesthetics: Targeted numbing. Local Anesthetic Incisional Infiltration: The surgeon may inject long-acting numbing medication (like Exparel) into the incision sites at the end of surgery. This can provide pain relief for 24–72 hours. Transversus abdominis plane (TAP) block: A nerve block performed by the anesthesiologist (using ultrasound) to numb the abdominal wall nerves. Very effective for abdominal incisions.  3. Opioid Medications (For Breakthrough Pain): These are used judiciously to cover pain that breaks through the foundation medications. Routes: PCA pump (see below), IV injections by nurses, or oral pills (e.g., oxycodone) as you transition off the IV.  4. Non-Pharmacological Methods: Ice Packs: Applied to the abdomen to reduce swelling and pain. Early Ambulation: Moving actually helps reduce pain and gas-related discomfort. Proper Positioning: Using pillows for support when lying or sitting.     Patient-Controlled Analgesia (PCA) Pump: How It Works & Duration  What it is: A programmable IV pump connected to you. It contains a bag of diluted opioid (like morphine or hydromorphone).  How YOU Use It: You have a button. When you feel pain, you press it. Safety Feature #1: The pump is programmed with a "lockout interval" (e.g., 8–10 minutes). If you press the button before that time has elapsed, it will not deliver a dose. This prevents overdose. Safety Feature #2: It may have a continuous background infusion at a very low rate, or it may be demand-only. Your anesthesiologist decides based on your needs. Key Benefit: It puts you in control. There’s no delay waiting for a nurse, and smaller, more frequent doses often provide steadier relief with less sedation than larger, occasional IV injections.   How Long Can a PCA Pump Be Used?  The use of a PCA pump is typically short-term and transitional, usually lasting 24 to 48 hours after surgery.  The standard pathway is: 1. Day of Surgery & Day 1: Pain is most acute. You will rely on the PCA pump plus the scheduled IV acetaminophen and NSAIDs. 2. Day 2 (or when you can tolerate liquids): The medical team will begin transitioning you to oral medications. You will start taking scheduled oral acetaminophen and an NSAID (e.g., ibuprofen). The PCA pump dose may be decreased, and you will be offered oral opioids (like oxycodone) for breakthrough pain. 3. Discontinuation: Once your pain is well-controlled on the oral regimen, and you are taking adequate fluids/food, the PCA pump will be discontinued (usually by the end of post-op day 1 or on day 2). The IV line may remain for fluids. 4. Discharge Plan: You will go home with a prescription for a limited supply of oral opioids (often only a 3-7 day supply) to be used *only as needed, in addition to scheduled non-opioid medications.   What to Communicate with Your Care Team: Your feedback is essential. Use a pain scale (0–10) to communicate effectively. Tell them if your pain is not controlled (e.g., consistently above 4-5/10 at rest). Crucially, report side effects: nausea, itching, excessive drowsiness, or difficulty breathing. These can be managed, often without sacrificing pain control.   Summary Pain Management is Proactive: It uses a layered approach (multi-modal) starting in the operating room. PCA is a Tool: It gives you safe, immediate control for the first 1–2 days. The Goal is Transition: The focus is to quickly move you from IV opioids (PCA) to a regimen of strong non-opioid foundations with oral opioids only as a "rescue." You are a Partner: Clear communication about your pain level and side effects allows the team to tailor your plan for optimal comfort and safety.  By understanding this plan, you can be an active participant in your recovery, using pain medication effectively to aid your healing without fear. |
| How long will the urinary catheter and drainage tubes remain? Is their removal painful? | After endometrial cancer surgery, urinary catheters and surgical drains are commonly used to monitor output and prevent fluid accumulation. Here’s a clear explanation of what to expect:     1. Urinary catheter (Foley catheter): Purpose:  Keeps the bladder empty during and immediately after surgery. Helps monitor urine output, which reflects kidney function and fluid balance.  Typical duration:  Minimally invasive (laparoscopic/robotic) surgery: usually 12–24 hours after surgery. Open surgery or extensive procedures: may remain 1–3 days, depending on swelling or difficulty urinating.  Removal:  Usually done by nursing staff. Pain or discomfort: Some patients feel mild burning, urgency, or pressure during removal. Most discomfort is temporary and tolerable.     2. Surgical drainage tubes (e.g., pelvic drains)  Purpose:  Remove blood, lymph fluid, or serous fluid that may accumulate in the surgical area. Helps prevent infection and seroma formation.  Typical duration:  Usually 2–5 days, depending on the amount and type of drainage. Drain output is monitored daily; once it drops below a certain level, the drain can be removed.  Removal:  Done by nursing staff, sometimes with a quick, gentle tug. Pain or discomfort: Mild pulling or stinging is common, but most patients tolerate it well. Local care instructions are given for the site afterward to prevent infection.     3. Tips for comfort and care: Relaxation and deep breathing can help reduce discomfort during removal. Follow instructions for drain site cleaning and dressing changes. Report fever, redness, swelling, or unusual drainage immediately—they could indicate infection. Early mobility and gentle walking do not interfere with drains and help overall recovery.     Bottom line: Urinary catheter: 1–3 days, mild temporary discomfort during removal. Drains: 2–5 days, mild pulling or stinging during removal. Both are temporary tools to ensure safety and proper healing, and removal is usually quick and tolerable.  I can also create a timeline showing exactly when catheters and drains are typically removed after minimally invasive vs open surgery, which helps you mentally prepare for each step. | These are very common and understandable concerns. The presence of tubes can feel like a significant hurdle in your recovery, but they are temporary and important tools. Here’s what you can expect.   1. The Urinary Catheter (Foley Catheter)  Purpose: To drain your bladder continuously during and immediately after surgery. This is crucial because:  1. The anesthesia and pain medications temporarily paralyze your bladder.  2. It allows accurate measurement of urine output, a key indicator of your hydration and kidney function.  3. It keeps the bladder empty and out of the way during pelvic surgery, promoting healing.  How Long Will It Remain?  For Minimally Invasive Surgery (Lap/Robotic): Often removed the morning after surgery (around 12–24 hours post-op). Sometimes, if you're recovering very well, it may even be removed the same evening.  For Open Surgery (Laparotomy): May stay in for 24–48 hours due to the longer recovery of bowel and bladder function.  Extended Use: It may stay longer if you have complications like difficulty voiding, or if a complex procedure was performed near the bladder.  Is Removal Painful?  The removal is very quick (about 10 seconds) and is typically described as a strange, brief "pinching" or burning sensation, not severe pain.  The nurse will ask you to take a deep breath and exhale as they gently pull the catheter out. The discomfort is over instantly.  You may feel a temporary urgency to urinate or slight burning with your first few voids afterward, which is normal.   2. Surgical Drains (e.g., Jackson-Pratt or JP Drains)  Purpose: These are soft bulbs with tubes placed near the surgical site to drain excess blood and lymphatic fluid that can accumulate after surgery. Important: Not all patients get these. They are more commonly used if you had an extensive lymph node dissection (to prevent a lymphocyst) or if there was more tissue disruption. How Long Will They Remain? This is highly variable and depends on the daily output volume.  The drain is usually removed once the output decreases to a low, acceptable level (often less than 20–30 mL per 24 hours).  This can take anywhere from a few days to a week or more. You may even go home with the drain in place, with instructions on how to empty and measure it. Is Removal Painful?  Removal is generally quicker than a catheter removal. You will feel a tugging and sliding sensation as the tube is pulled from the tunnel under your skin. It can be briefly uncomfortable or slightly pinchy, but it’s over in seconds. The area may be sore for a short time afterward.   Your Role & Key Points  Communication is Key: Report any severe pain, redness, or foul-smelling discharge around the tube sites. Activity with Tubes: You can walk with both a catheter (the bag can be hung on your IV pole) and drains (pinned to your gown). Walking is encouraged! Milestone of Removal: Having the catheter and drains removed are positive milestones in your recovery. They mark your body's progress in healing and regaining function.  Summary: Catheter: Expect it for ~1 day (laparoscopic) or 1–2 days (open). Removal is a brief, odd sensation, not intensely painful. Drains: Used only in specific cases. Stay in until drainage slows, often for several days. Removal involves a quick tugging sensation.  These tubes are there to help you heal safely. Your care team will remove them at the earliest possible moment to support your comfort and mobility. |
| How soon after surgery can I get out of bed, eat, and drink? What dietary restrictions and recommendations are there? | After endometrial cancer surgery, recovery protocols focus on early mobilization, gradual diet progression, and preventing complications. Here’s a detailed breakdown:     1. Getting out of bed (early mobilization): Why it’s important:  Reduces the risk of blood clots (DVT/PE) Prevents lung complications like pneumonia Stimulates bowel function and speeds recovery  Typical timeline:  Minimally invasive surgery (laparoscopic/robotic): Usually a few hours after surgery, with assistance, patients are encouraged to sit up and take short walks in the room. Open surgery: often 12–24 hours post-op, depending on anesthesia recovery and pain control. Start with short, frequent walks, gradually increasing distance as tolerated.     2. Eating and drinking: Immediate post-op:  Clear liquids (water, tea, broth, juice without pulp) are usually allowed 6–12 hours after surgery if no nausea or bowel issues. Advance diet gradually:   Clear liquids → soft foods → regular diet over 1–2 days.  Open surgery:  Bowel function may take longer to return; surgeons often wait for the first passing of gas or bowel sounds before advancing the diet.     3. Dietary recommendations: Early recovery diet:  High-protein foods: eggs, yogurt, lean meats, fish, tofu—supports wound healing. Fruits and vegetables: vitamins and fiber to prevent constipation. Whole grains: oats, brown rice, whole wheat bread, for energy and gut function. Hydration: water and clear fluids are critical; avoid sugary drinks if diabetic.  Foods to avoid initially:  Fatty, fried, or heavy meals (may cause nausea or bloating) Carbonated drinks or gas-producing foods (beans, cabbage) if bloating occurs Alcohol until cleared by your doctor     4. Practical tips: Eat small, frequent meals at first. Take pain medications with food if they upset your stomach. Monitor bowel movements; constipation is common after anesthesia and pain meds. Gradually return to a normal diet as tolerated, aiming for balanced nutrition to support recovery.     Bottom line: Out of bed. Within hours for minimally invasive, 12–24 hrs for open surgery. Clear liquids: typically the same day or the next morning. Soft/regular diet: 1–2 days, as tolerated. Focus: high-protein, fiber-rich, easily digestible foods; avoid heavy/fatty foods early. Early activity and a careful diet speed recovery, reduce complications, and improve overall comfort.  I can also make a day-by-day post-op activity and diet schedule for the first week after surgery, so you know exactly when to walk, eat, and progress your diet safely. | These are excellent, practical questions. Knowing what to expect in the immediate hours and days after surgery can greatly reduce anxiety. Here’s a clear, stage-by-stage guide.   Timeline: Getting Moving, Eating, and Drinking  Modern recovery protocols emphasize getting you back to normal functions as quickly as safely possible to prevent complications.  In the Recovery Room (PACU):  Drinking: You may be allowed small sips of ice chips or water as you wake up, if you're not nauseated.  Moving: You'll be encouraged to move your legs and feet while in bed.  Back in Your Hospital Room (Typically a Few Hours Post-Op):  Getting Out of Bed: You will be helped to sit up, stand, and take a few steps with assistance the same day of surgery, often within 4–6 hours. This is non-negotiable and critical for preventing blood clots and pneumonia. It may be uncomfortable, but it is safe and necessary.  Drinking: You'll progress to clear liquids (water, broth, juice, gelatin). The goal is to see if your stomach and intestines are "waking up" from anesthesia.  Eating: Solid food is usually introduced the day after surgery, once you can tolerate liquids without nausea and you start passing gas (a sign your bowels are working). It begins with a light, bland diet (toast, crackers, soup).   Dietary Recommendations & Restrictions Post-Surgery  The primary goals are to avoid nausea, support healing, and prevent constipation.  Phase 1: Initial Hospital Diet (Clear Liquids to Bland Foods) Start Slowly: Follow the nurse's guidance. Don't rush to eat a large meal. Bland is Best: Stick to easily digestible foods like broth, saltines, toast, applesauce, bananas, rice, and plain chicken. Avoid: Greasy, fatty, spicy, or gas-producing foods (beans, broccoli, carbonated drinks) initially.  Phase 2: Diet at Home for the First 1–2 Weeks (The Healing Diet) Priority #1: Protein: Essential for tissue repair and immune function. Include: Greek yogurt, eggs, cottage cheese, fish, poultry, tofu, lentils, and protein shakes. Priority #2: Fiber + Hydration: This is your defense against opioid-induced constipation, a major post-op issue.  Fiber: Prunes, oats, pears, apples (with skin), beans, and vegetables. Increase gradually to avoid bloating.  Hydration: Drink plenty of water, herbal tea, and clear fluids. Aim for 8+ glasses a day. Foods to Emphasize:  Colorful Fruits & Vegetables: For vitamins, minerals, and antioxidants.  Whole Grains: For steady energy and fiber. Foods to Limit/Avoid:  Constipation Culprits: Excessive cheese, red meat, and processed foods.  Gas & Bloating: Carbonated drinks, cruciferous vegetables (if they bother you), and chewing gum.  Inflammatory Foods: High-sugar snacks and fried foods can hamper healing.   Key Dietary Principles to Remember: 1. Listen to Your Body: Eat small, frequent meals instead of three large ones. 2. Constipation is Common (and Treatable): Along with diet, you will likely be sent home with a stool softener (like Colace) and possibly a mild laxative (like Miralax). Start them as directed, don't wait. Straining is painful and dangerous after pelvic surgery. 3. Gas Pain is Normal: The CO₂ gas used in laparoscopic surgery can cause shoulder tip pain. Walking is the best remedy to help your body absorb it.   Red Flags: When to Call Your Doctor While some bloating and appetite changes are normal, contact your care team if you experience: Persistent nausea/vomiting that prevents you from keeping liquids down. Severe, cramping abdominal pain that doesn't improve. Inability to pass gas or have a bowel movement for 4–5 days despite following the regimen. Signs of dehydration (dark urine, dizziness, extreme thirst).  Summary: You'll be moving and sipping water the same day. Eating solid food resumes the next day. At home, focus on a high-protein, high-fiber, hydration-focused diet to power your healing and keep your digestive system moving smoothly. Your team will guide you every step of the way. |
| How should the wound be cared for? When can I shower? Do the minimally invasive puncture sites require special care? | Proper wound care after endometrial cancer surgery is critical to prevent infection, promote healing, and reduce scarring. The exact care depends on whether you had open surgery or minimally invasive (laparoscopic/robotic) surgery.     1. General wound care principles: Keep the incision clean and dry initially. Wash hands before touching dressings or the wound. Monitor for signs of infection: redness, warmth, swelling, pus, or increased pain. Avoid strenuous activity that stretches the incision for at least 2–4 weeks (or as advised by your surgeon).     2. Showering: Minimally invasive surgery (laparoscopic/robotic):  Small puncture sites usually have steri-strips or small adhesive dressings. Shower: often allowed 24–48 hours after surgery, but check if dressings should be left on until the first follow-up. Avoid soaking in baths, hot tubs, or swimming pools until the wounds are fully healed (usually 1–2 weeks).  Open surgery (larger abdominal incision):  Showering may be delayed 2–3 days or until the incision is dry and sealed. Pat dry gently; do not rub.     3. Minimally invasive puncture sites: Usually 1–2 cm small incisions. Keep dressings dry until instructed to remove or replace. Steri-strips or glue: do not peel off; they usually fall off naturally within 1–2 weeks. Avoid applying creams or ointments unless your doctor advises. Check for redness, swelling, or drainage—report promptly if present.     4. Dressing changes: Some centers allow daily gentle cleansing with mild soap and water once dressings are removed. Cover with a sterile gauze only if the wound is still oozing or at risk of friction. Avoid tight clothing that rubs or irritates the incision.     5. Pain management at the incision site: Mild pain, numbness, or tingling around the incisions is normal. Over-the-counter pain medication or your prescribed analgesics help. Ice packs can reduce swelling if tolerated; avoid direct ice contact with the skin—wrap in a towel.     Bottom line: Minimally invasive wounds: small, heal fast. Simple care—keep clean and dry, shower as allowed. Open surgery wounds: larger incision, may need a few more days before showering, careful monitoring. Watch for infection, redness, swelling, or unusual drainage. Steri-strips/glue should stay in place until naturally removed.  I can also make a wound care schedule for the first 2 weeks post-op, showing when to shower, change dressings, and monitor for complications—very practical for minimizing infection risk. | Excellent questions. Proper wound care is crucial for preventing infection and ensuring a good cosmetic outcome. The guidelines differ slightly between open and minimally invasive incisions.  Here’s a clear, step-by-step guide.   General Wound Care Principles (For All Incisions)  1. Keep it Clean and Dry: This is the golden rule until the skin is fully sealed. 2. Hand Hygiene: Always wash your hands with soap and water before touching near your incision sites. 3. Inspect Daily: Look at your wounds each day during care for signs of infection (see "Red Flags" below). 4. Let it Breathe: After showering and patting dry, you may leave the wounds uncovered or cover them with fresh, dry gauze if there's drainage or rubbing against clothing. Avoid heavy creams or ointments unless prescribed.   When can I shower?  Timeline: You can usually shower 24–48 hours after surgery, once any bulky dressings are removed. Your discharge instructions will confirm the exact timing. How to Shower Safely:  Avoid Baths, Hot Tubs, and Pools: Do not submerge the wounds in water (tub bath, swimming) for at least 4-6 weeks, or until your surgeon says it's safe.  Shower Technique: Let soapy water run gently over the incisions. Do not scrub. Pat the areas completely dry with a clean towel. Do not rub.   Care for Minimally Invasive (Laparoscopic/Robotic) Puncture Sites: These small incisions (usually 0.5–1.5 cm) are often closed with dissolvable sutures under the skin and sealed with surgical glue (Dermabond) or steri-strips (thin adhesive strips).  Surgical Glue: It will form a hard, waterproof seal that will flake off on its own in 7–14 days. Do not pick, peel, or scrub it. Steri-Strips: Leave them in place. They will curl and fall off on their own, usually in 5–10 days. You can gently trim any loose edges with clean scissors. Special Care: These sites require less maintenance. Just keep them clean and dry. They may bruise more visibly than a larger incision.   Care for an Open Incision (Laparotomy): This vertical or horizontal incision is closed in layers, often with dissolvable internal sutures and either staples, external sutures, or surgical glue on the surface.  If you have staples or non-dissolvable sutures: These will need to be removed by a nurse or your surgeon, typically 7–10 days after surgery. The wound must stay dry until they are out and the tiny holes have sealed (another 24–48 hours). Support Your Incision: Use your hand or a small pillow to splint the incision when you cough, sneeze, laugh, or get out of bed. This reduces pain and stress on the healing tissue. Clothing: Wear loose, soft, high-waisted pants or dresses to avoid rubbing.   Red Flags: When to Call Your Doctor Immediately  Contact your surgeon's office if you notice: Signs of Infection: Increasing redness, warmth, swelling, or foul-smelling drainage (yellow/green pus). Fever: Temperature above 101°F (38.3°C). Opening of the Wound: Any part of the incision reopens or gaps. Excessive Bleeding: Soaking through a bandage. Increasing Pain: Pain that worsens significantly instead of gradually improving. Red Streaks: Red streaks spreading from the incision site.   Summary Checklist  - [ ] Shower: Okay in 24–48 hrs. No baths/pools for 4-6 weeks. - [ ] Pat Dry: Never rub. - [ ] Laparoscopic Sites: Leave glue/strips alone to fall off naturally. - [ ] Open Incision: Protect it, splint when moving, and watch for staple/suture removal date. - [ ] Inspect daily for signs of infection. - [ ] Wear loose, non-irritating clothing.  Following these guidelines will promote smooth healing. Your discharge paperwork will have specific contact information for your surgical team—never hesitate to call with concerns. |
| What are the dangerous "warning signs" after surgery? (e.g.(fever, redness/discharge from the wound, leg swelling, difficulty breathing, abnormal abdominal pain, etc.) | After endometrial cancer surgery, there are certain warning signs that should never be ignored, because they can indicate serious complications such as infection, blood clots, bleeding, or bowel/urinary problems. Here’s a detailed guide:     1. Infection  Signs to watch for:  Fever ≥ 38 °C (100.4 °F) or persistent low-grade fever Redness, swelling, warmth, or increased pain at incision sites Pus or foul-smelling drainage from the wound or drain site Chills, malaise, or worsening fatigue  Action: Contact your surgeon immediately. Early antibiotics or intervention may be needed.     2. Bleeding or hematoma  Signs:  Sudden increase in drainage from surgical drains Bright red bleeding from the incision Large swelling or bruising around the wound Feeling faint, dizzy, or rapid heart rate (may indicate internal bleeding)  Action: Seek urgent medical care; severe bleeding may require readmission.     3. Blood clots (DVT/PE): DVT (deep vein thrombosis) signs:  Swelling in one leg (often the calf) Pain, tenderness, or cramping in the leg Red or warm skin over the leg  PE (pulmonary embolism) signs:  Sudden shortness of breath Chest pain, especially with breathing Rapid heartbeat or palpitations Coughing up blood (rare but serious)  Action: Emergency—call 911 or go to the ER immediately.     4. Bowel problems: Signs:  Severe or worsening abdominal pain or bloating Inability to pass gas or stool for more than 2–3 days Nausea, vomiting, or persistent vomiting Distended or hard abdomen  Action: Contact your doctor promptly; this may indicate bowel obstruction or ileus.     5. Urinary problems: Signs:  Inability to urinate or very low urine output, Burning, pain, or blood in the urine, Fever or flank pain (may indicate a urinary tract infection or obstruction)  Action: Notify your surgeon immediately.     6. Other concerning symptoms: Severe, uncontrolled pain despite medication; sudden weakness, numbness, or neurological changes; persistent dizziness, fainting, or rapid heartbeat.     Bottom line: After surgery, Immediate medical attention is warranted for any of the above symptoms. Early recognition can prevent serious complications.  Quick reference summary:  \| Category \| Warning signs \| Action \| \| - \| \| Infection \| Fever, redness, pus, swelling \| Contact doctor \| \| Bleeding \| Increased drainage, bright red blood, dizziness \| Urgent care \| \| Blood clot \| Leg swelling, pain, chest pain, shortness of breath \| Emergency \| \| Bowel \| Severe pain, vomiting, bloating, no gas/stool \| Call doctor \| \| Urinary \| Painful urination, low output, blood \| Call doctor \|    If you want, I can make a concise “red flag checklist” for the first 2–3 weeks after surgery, so you can easily monitor yourself daily. This is very useful for both you and your caregivers. | This is the most important list to know. Recognizing and acting on these warning signs promptly can prevent life-threatening complications. If you experience any of the following, contact your surgeon's office immediately or go to the nearest emergency room.  Here is a categorized list of dangerous post-operative warning signs.   Category 1: Life-Threatening Emergencies (Go to the ER) Difficulty Breathing / Shortness of Breath:  Especially if sudden, severe, or accompanied by: Chest pain (sharp, worse with deep breathing), rapid heart rate, coughing (especially if bloody), feeling faint, or extreme anxiety.  Why: This is the #1 symptom of a Pulmonary Embolism (PE)—a blood clot in the lungs. It is a medical emergency.  Chest Pain or Pressure:  Why: Could indicate a heart attack or a large PE.  Heavy, Uncontrolled Bleeding:  Soaking through a maxi-pad or wound dressing in an hour.  Why: May indicate hemorrhage from the surgical site.   Category 2: Signs of Infection (Call the Surgeon Immediately) Fever: Temperature above 101°F (38.3°C). A low-grade fever (99-100°F) is common in the first 24–48 hours due to inflammation, but a high or persistent fever is a red flag. Wound Infection Signs:  Increasing redness, warmth, or swelling spreading from the incision.  Foul-smelling, thick, yellow, or green pus (some clear or blood-tinged drainage is normal initially).  The wound edges are pulling apart. Severe Abdominal Pain: Pain that is significantly worsening instead of gradually improving, or is localized, sharp, and constant.   Category 3: Signs of Blood Clot (DVT) or Bleeding Deep Vein Thrombosis (DVT) in the Leg:  New, persistent pain in one calf or thigh (not both).  Swelling in one leg (measure if unsure).  Redness, warmth, and tenderness in one leg. Signs of internal bleeding or anemia:  Feeling dizzy, lightheaded, or like you might faint (especially when standing).  Rapid heart rate (palpitations).  Unusual and severe fatigue or weakness.   Category 4: Gastrointestinal & Urinary Red Flags: Abnormal Abdominal Pain & Distension:  Severe, cramping pain with an inability to pass gas or have a bowel movement for 4–5 days (despite using stool softeners).  A hard, swollen, and tight belly with nausea/vomiting. This could indicate a bowel obstruction. Urinary Problems:  Inability to urinate for 8+ hours after catheter removal, despite feeling the urge.  Painful urination with fever/chills (sign of a UTI).   A Handy "RED FLAG" Mnemonic: CALL M.D. C – Chest pain, trouble breathing A – Abdominal pain that is severe or worsening L – Leg pain/swelling (one side) L – Leaking (pus) from the incision M – Mental change (confusion, extreme fatigue) D – Dizziness, fainting, Fever >101°F   What to Do 1. Don't Panic, But Don't Wait. Trust your instincts. 2. Call Your Surgeon's Office First. They have a 24/7 line. Describe your symptoms clearly. They will tell you if you need to go to the ER or come to their clinic. 3. If symptoms are severe (chest pain, difficulty breathing), call 911 or go to the ER directly. Tell them you recently had major abdominal/pelvic surgery for cancer.  Having this list empowers you to be a partner in your recovery. It's always better to call and have it be a minor issue than to ignore a major one. Your surgical team expects and wants you to report these concerns. |
| What are the common postoperative complications (e.g., lymphocele, urinary tract infection, intestinal obstruction, etc.)? How can they be prevented and managed? | After endometrial cancer surgery, most patients recover uneventfully, but there are several potential postoperative complications. Understanding them helps in prevention, early detection, and management.     1. Lymphocyst / lymphatic fluid accumulation: Cause:  Removal of pelvic or para-aortic lymph nodes can leave lymph fluid accumulating in the pelvis.  Signs:  Pelvic or lower abdominal swelling, feeling of fullness or pressure, sometimes pain or urinary/bowel symptoms  Prevention:  Meticulous surgical technique to seal lymphatic channels Early ambulation to promote lymph drainage Drain placement if risk is high  Management:  Often resolves on its own If large or symptomatic, may require ultrasound-guided drainage; rarely, sclerotherapy or surgical intervention     2. Urinary tract infection (UTI)  Cause:  Indwelling catheter use or urinary retention post-surgery  Signs:  Painful or frequent urination, fever, cloudy or foul-smelling urine  Prevention:  Remove the catheter as soon as safely possible Maintain good hydration; clean catheter care if needed  Management:  Antibiotics based on urine culture; ensure adequate hydration and monitor kidney function     3. Intestinal obstruction (postoperative ileus or adhesion-related obstruction)  Cause:  Anesthesia, handling of intestines during surgery, adhesions  Signs:  Abdominal bloating Nausea and vomiting Inability to pass gas or stool  Prevention:  Minimally invasive surgery reduces risk Early mobilization Gradual diet progression (clear liquids → soft foods → normal diet)  Management:  Nasogastric tube for decompression if severe IV fluids and electrolyte correction Surgery only if obstruction does not resolve     4. Wound infection or dehiscence. Cause:  Bacteria at the incision site, poor blood supply, obesity, diabetes. Prevention:  Keep the incision clean and dry. Good glycemic control if diabetic. Proper surgical technique. Management: Antibiotics. Local wound care or dressing changes. Rarely, surgical debridement.     5. Deep vein thrombosis (DVT) / pulmonary embolism (PE)  Cause:  Immobility, cancer-related hypercoagulability  Prevention:  Early ambulation Compression stockings or pneumatic devices Prophylactic anticoagulants for high-risk patients  Management:  Anticoagulation therapy Hospitalization if PE is suspected     6. Lymphedema of the legs: Cause:  Lymph node removal disrupting lymphatic drainage  Signs:  Swelling in the legs, heaviness, discomfort Sometimes skin changes or infections  Prevention:  Avoid injury or infection in the legs. Elevation and gentle exercise  Management:  Compression stockings Physical therapy / lymphatic drainage massage Prompt treatment of infections     7. Urinary or bowel dysfunction: Temporary difficulty urinating due to nerve handling; constipation or delayed bowel movements due to anesthesia or pain medications. Prevention:  Early mobilization, adequate hydration, and stool softeners if necessary. Management:  Bladder scans and temporary catheterization if needed; laxatives or enemas under medical supervision.     Bottom line: Most complications can be prevented or minimized by:  1. Early mobilization 2. Good catheter and wound care 3. Adequate hydration and nutrition 4. Careful monitoring for signs of infection, bleeding, or clots 5. Follow-up visits for drains, lymph nodes, and labs  Early recognition and intervention are key—delays can lead to more serious problems.  I can also create a “postoperative complications table,” showing for each complication: risk factors, prevention steps, early warning signs, and management, which is very practical for home monitoring. Do you want me to do that? | This is a crucial question. Being aware of potential complications, their prevention, and management empowers you to participate actively in your recovery. While not everyone experiences these, here is a guide to the most common post-operative issues after endometrial cancer surgery.   Common Post-Operative Complications #1. Infection Types: Surgical Site Infection (SSI) at the incision, Urinary Tract Infection (UTI) from the catheter, and Pelvic Infection. Prevention: Pre-op antibiotics given by your surgeon, sterile technique during surgery, early catheter removal, and proper wound care (keeping it clean and dry). Management: Antibiotics (oral or IV). For a wound infection, may require opening the incision to drain pus.  # 2. Urinary Complications: Urinary Retention: Inability to empty the bladder fully after catheter removal. Common due to anesthesia/pain medications. UTI: As above. Prevention: Early catheter removal, adequate hydration, and "bladder training" (timed voiding). Management: If retention occurs, a new catheter may be needed for a few days to let the bladder rest. UTI treated with antibiotics.  # 3. Gastrointestinal Issues: Ileus: A temporary, predictable paralysis of the bowel after surgery. Causes bloating, nausea, and no passing of gas. Small Bowel Obstruction (SBO): A more serious blockage, often from adhesions (scar tissue). Causes severe cramping pain, vomiting, and distension. Prevention: Early ambulation, chewing gum (stimulates the gut), avoiding heavy narcotics as soon as possible, and following a diet progression. Management: Ileus is managed with IV fluids, stopping oral intake, and waiting. SBO may require a nasogastric (NG) tube to suction and decompress the stomach, and possibly repeat surgery if not resolved.  # 4. Thromboembolic Events (Blood Clots): Deep Vein Thrombosis (DVT): A clot in a leg vein. Pulmonary Embolism (PE): A clot that travels to the lungs (a medical emergency). Prevention: The cornerstone of your care. Includes sequential compression devices (SCDs) during/after surgery, early and frequent ambulation, and blood-thinning injections (enoxaparin/heparin). Management: Immediate initiation of therapeutic anticoagulation (stronger blood thinners) for several months.  # 5. Lymphatic Complications (After Lymph Node Removal) Lymphocyst: A fluid collection (seroma) in the pelvic cavity where lymph nodes were removed. Lymphedema: Chronic swelling of the leg(s) due to disrupted lymph flow. Prevention: Sentinel lymph node biopsy (vs. full dissection) drastically reduces risk. Some surgeons leave drains in place to prevent fluid buildup. Management: Small, asymptomatic lymphocysts are often just monitored. Large or symptomatic ones may be drained with a needle under ultrasound guidance. Lymphedema is managed by certified therapists with compression garments, manual drainage, and exercise.  # 6. Bleeding (Hemorrhage) Hematoma: A collection of blood under the incision or in the pelvis. Prevention: Meticulous surgical technique, management of blood thinners. Management: Small hematomas often reabsorb. Significant bleeding may require a blood transfusion or, rarely, a return to surgery (re-operation).   General Prevention Strategy: Your Role is Key: Many complications are mitigated by the same core actions: 1. Early & Frequent Ambulation: The #1 thing you can do. Prevents clots, ileus, and pneumonia. 2. Incentive Spirometer Use: Deep breathing exercises to prevent lung atelectasis/pneumonia. 3. Hydration: Drink plenty of water. 4. Adherence to Medication: Take blood thinners, stool softeners, and pain meds as prescribed. 5. Wound Care: Follow incision care instructions meticulously.   Management Philosophy: Early Recognition is Everything Your surgical team's goal is to prevent these issues. If they occur, the focus shifts to early recognition and intervention to prevent them from becoming severe.  When to Call Your Doctor: Refer to the "warning signs" list (fever >101°F, redness/drainage, leg swelling, difficulty breathing, severe abdominal pain, inability to urinate). Reporting early allows for outpatient management (e.g., oral antibiotics) instead of a crisis requiring re-hospitalization.  Summary: Modern surgery focuses on minimizing risks through technique (such as minimally invasive surgery and sentinel nodes) and strict protocols (such as clot prevention). By understanding these potential complications, you can be a vigilant partner in your recovery, knowing which symptoms warrant an immediate call to your care team. |
| How long will I likely need to stay in the hospital? What are the criteria for discharge? | Hospital stay after endometrial cancer surgery depends on the type of surgery, your recovery, and whether complications occur. Here’s a breakdown:     1. Typical length of stay  \| Surgery type \| Typical hospital stay \| \| \| -- \| \| Minimally invasive (laparoscopic/robotic) \| 1–3 days, sometimes same-day discharge if very fast recovery \| \| Open abdominal surgery (laparotomy) \| 3–7 days, depending on pain control, bowel function, and mobility \|  Notes:  Patients with complications (infection, bleeding, delayed bowel function) may need a longer stay. Younger, healthy patients often recover faster.     2. Criteria for Discharge Before going home, the medical team usually ensures:  a. Pain control  Pain is manageable with oral medications; no need for IV pain pumps.  b. Mobility: Able to walk safely and perform basic self-care (toileting, dressing).  c. Bowel and bladder function: Urinating without a catheter or minimal urinary retention, passing gas, or having a bowel movement (especially after open surgery)  d. Wound and drain status: Surgical wounds are stable, with no excessive drainage; drains are removed or safe for home care if left in place  e. Vital signs and labs: Stable; normal heart rate, blood pressure, and temperature; blood counts and electrolytes acceptable  f. Patient education and support: You and caregivers know wound care, medications, activity restrictions, and warning signs; a follow-up appointment is scheduled.     3. Practical tips: Arrange transportation home; you may be groggy from anesthesia or pain meds. Have help at home for the first few days, especially after open surgery. Stock up on meals and supplies in advance for easy recovery.     Bottom line: Minimally invasive surgery: 1–3 days if recovery is smooth Open surgery: 3–7 days Discharge depends on pain control, mobility, bowel/bladder function, wound status, and stable vital signs. Early discharge is possible if all criteria are safely met, even after major surgery.  I can also make a postoperative recovery timeline showing what milestones are usually met each day in the hospital and at home, so you can anticipate discharge and recovery steps. | This is a key question for planning and setting expectations. Hospital stays have become significantly shorter due to advances in minimally invasive surgery and enhanced recovery protocols.   How Long Will I Likely Stay in the Hospital?  The length of stay depends primarily on the surgical approach:  Minimally Invasive Surgery (Laparoscopic/Robotic):  Typical Stay: 1 to 2 nights. Many patients go home on post-operative day 1. This is now the most common pathway for early-stage disease. Open Surgery (Laparotomy):  Typical Stay: 2 to 4 nights. The larger incision requires more time for pain management and the return of bowel function.  Important Note: These are averages. Your stay may be shorter or longer based on your individual recovery, pain control, and whether any complications arise.     What are the criteria for discharge?  You are not discharged simply because a certain number of days have passed. You must meet specific safety milestones that show your body is recovering adequately to continue healing at home. The clinical team will assess:  1. Vital Signs are Stable: No fever, with a normal heart rate and blood pressure. 2. Pain is Controlled with Oral Medication: You must be able to manage your pain with pills (e.g., acetaminophen, ibuprofen, and a limited supply of opioids), not needing IV pain medications. 3. You Are Tolerating a Diet: You are drinking fluids and eating solid food without significant nausea or vomiting. 4. Your gastrointestinal system is "working": you have passed gas and, ideally, had a bowel movement. This is a key sign that your intestines are recovering from anesthesia. 5. You Are Mobilizing Independently: You can get out of bed, walk to the bathroom, and take walks in the hallway with minimal assistance. This is critical for preventing clots and pneumonia. 6. You Can Urinate Normally: After the catheter is removed, you must be able to empty your bladder completely without difficulty. 7. Your Wound Looks Good: No signs of active infection or concerning drainage. 8. You and Your Caregiver Understand Home Care Instructions: This includes knowing how to care for your incisions, what medications to take, which activities to avoid, and what warning signs require an immediate call to the doctor.   What to Expect on Discharge Day You will receive detailed written instructions covering medication, wound care, activity restrictions, diet, and follow-up appointment dates. Your nurse will review these instructions with you and your caregiver. You will be given a contact number for your surgical team for urgent questions.   Summary: The Path to Going Home Your medical team's goal is to support you in meeting these milestones safely and efficiently. Your active participation—walking, using the incentive spirometer, and reporting your progress—directly helps you achieve them faster.  You can ask your surgeon beforehand: "Based on the planned surgical approach, what is the expected hospital stay for a patient like me?" This will give you a realistic framework, but always remember the final decision is based on your safe, individual recovery on the day. |
| How soon after discharge can I resume normal life and work? When can I exercise (e.g., walk, swim) and resume sexual activity? | Recovery after endometrial cancer surgery varies depending on the surgery type, age, overall health, and any complications, but there are general timelines for resuming activities:     1. Returning to daily life and work  \| Activity \| Minimally invasive surgery \| Open abdominal surgery \| \| -- \| -- \| - \| \| Light daily activities (dressing, cooking, short walks) \| 1–3 days \| 3–7 days \| \| Household chores / errands \| 1–2 weeks \| 2–4 weeks \| \| Return to work (sedentary/desk job) \| 2–4 weeks \| 4–6 weeks \| \| Return to physically demanding work \| 4–6 weeks \| 6–8 weeks \|  Notes:  Fatigue may persist for weeks, so gradually increase activity. Listen to your body—pain, dizziness, or swelling means slow down.     2. Exercise: Walking is encouraged within hours after surgery in the hospital for minimally invasive surgery, 1–2 days after open surgery. Gradually increase duration and distance.  Moderate exercise (yoga, cycling, light jogging):  Usually 4–6 weeks after surgery, depending on healing.  Swimming or soaking in pools/baths:  Wait until incisions are fully healed (typically 2–4 weeks). Avoid hot tubs until cleared, due to infection risk.  Strength training / lifting heavy objects:  Avoid lifting >5–10 kg for at least 4–6 weeks after open surgery; minimally invasive recovery may be faster.     3. Sexual activity can usually be resumed 4–6 weeks after surgery, once:   Incisions are healed  Pain is controlled  No vaginal bleeding or discharge Using lubrication may help if vaginal dryness occurs, especially if ovaries were removed. Discuss fertility and hormonal concerns with your doctor if ovary removal or fertility-sparing surgery was done.     4. Practical tips for smooth recovery: Gradually increase activity and avoid overexertion. Follow wound care and hygiene guidelines to prevent infection. Maintain hydration, a balanced diet, and protein intake to support healing. Track any new symptoms (fever, swelling, bleeding, or unusual pain) and report promptly.     Bottom line: Light daily activities: start almost immediately post-op. Return to work: 2–6 weeks, depending on surgery type and job demands. Exercise: walking immediately, moderate exercise in 4–6 weeks, swimming after wound healing. Sexual activity: usually 4–6 weeks post-op. Recovery is individualized—follow your surgeon’s guidance and gradually build up activity rather than rushing.  I can make a detailed week-by-week post-discharge activity guide, including walking, diet, exercise, and sexual activity, so you know exactly what is safe at each stage. | This is a critical part of planning your recovery. Resuming activities is a gradual, phased process guided by your body's healing. Here is a general timeline based on the type of surgery, but always follow your surgeon's specific instructions, as they know the details of your procedure.   General Recovery Timeline & Guidelines: Your body needs approximately 6–8 weeks for initial internal healing (for ligaments, connective tissue, and the vaginal cuff). Use this as a framework, but let pain and fatigue be your guide.     1. Resuming Work Desk Job / Sedentary Work:  Minimally Invasive Surgery: 2–4 weeks. Many can return part-time or full-time if flexible.  Open Surgery: 4–6 weeks. Physically Demanding Job (lifting, standing, manual labor):  Minimally Invasive Surgery: 6–8 weeks minimum, and may require temporary work restrictions.  Open Surgery: 8 weeks or more, often requiring a formal work-release note from your surgeon.  Key Point: Discuss your specific job demands with your surgeon. Fatigue is a major factor; you may need a phased return (e.g., half-days).     2. Daily Activities & Household Chores Walking: Encouraged immediately. Start with short, frequent walks at home, gradually increasing distance and pace. This is your primary exercise for the first few weeks. Driving: Typically allowed 2–4 weeks post-op, but only when:  1. You are off narcotic pain medication.  2. You can react quickly without hesitation (test by simulating braking).  3. You can turn your body to check blind spots without significant pain. Lifting: STRICT RESTRICTION: Do not lift anything heavier than 10 lbs (about a gallon of milk) for 6-8 weeks. This includes groceries, children, laundry baskets, and heavy pots. This is crucial to prevent hernia and internal injury. Housework: Avoid vacuuming, mopping, and making beds for 4-6 weeks. Light cooking and tidying are okay if they don't involve bending or lifting.     3. Exercise Walking: Start Day 1. The best and safest exercise initially. Climbing Stairs: Go slowly. One step at a time. You can do this from the start, but limit frequency initially. Low-Impact Aerobics/Stationary Bike: May be allowed at 4–6 weeks with your surgeon's okay. Swimming & Baths: Absolutely not until your surgeon explicitly clears you, usually at your 6-week post-op checkup, provided your vaginal cuff is fully healed. Submerging in water before then risks a serious infection. Running, high-impact activities, weight lifting: not before 8 weeks, and often longer. Requires explicit clearance.     4. Resuming Sexual Activity This is one of the most important questions. You must wait for your surgeon's explicit "all clear," typically at your 6-week postoperative visit.  Why the Wait? The top of the vagina is surgically closed to form a "vaginal cuff." It needs 6-8 weeks to heal completely. Penetration before this cuff is healed can cause a serious tear, infection, or bleeding that may require emergency surgery. At the 6-Week Check: Your surgeon will examine you to confirm the cuff is healed. When Resuming: Go slowly, use lubrication, and communicate with your partner. Some discomfort or tightness is normal initially. Stop if there is pain or bleeding, and inform your doctor.   The "Listen to Your Body" Principle: Fatigue is Normal: Your energy will come in waves. Rest when you are tired; it's part of healing. Pain is a Signal: If an activity causes pain (beyond mild soreness), stop. You are doing too much too soon. Swelling is a Sign: Increased abdominal swelling at the end of the day means you've been overly active. Scale back the next day.   Summary: The Phased Approach Weeks 1–2: Focus on resting, walking around the house, and basic self-care. No lifting. Weeks 3–4: Increase walking distance. May return to a desk job. Light household tasks. No driving if on opioids. Weeks 5–8: Gradual return to most normal activities, but still no heavy lifting, swimming, or sex until cleared. After 8 Weeks: With your surgeon's approval, you can begin to reintegrate most exercises and activities.  Your final, A personalized timeline will be given at your post-operative checkups. Patience during this recovery period is an investment in your long-term health and to prevent complications that could set you back significantly. |
| How soon after discharge can I resume normal life and work? When can I exercise (e.g., walk, swim) and resume sexual activity? | After endometrial cancer surgery, recovery timelines vary depending on the surgery type, your overall health, and whether there were any complications, but general guidance is as follows:     1. Daily life and work  \| Activity \| Minimally invasive surgery \| Open abdominal surgery \| \| -- \| -- \| - \| \| Light daily activities (dressing, cooking, short walks) \| 1–3 days \| 3–7 days \| \| Household chores / errands \| 1–2 weeks \| 2–4 weeks \| \| Return to sedentary work \| 2–4 weeks \| 4–6 weeks \| \| Return to physically demanding work \| 4–6 weeks \| 6–8 weeks \|  Tips:  Fatigue is common for several weeks; increase activity gradually. Pain, dizziness, or swelling are signals to slow down.     2. Exercise: Walking is encouraged immediately in hospital for minimally invasive surgery; 1–2 days post-op for open surgery. Moderate exercise (yoga, cycling, light jogging): usually 4–6 weeks post-op. Swimming or baths: only after incisions are fully healed, usually 2–4 weeks. Heavy lifting: avoid >5–10 kg for 4–6 weeks after open surgery; minimally invasive recovery may allow a faster return.     3. Sexual activity: Generally safe to resume 4–6 weeks post-op, once:   Incisions are healed  Pain is minimal  No abnormal bleeding or discharge  Lubrication may help if vaginal dryness occurs, especially if ovaries were removed.  Discuss fertility or hormonal concerns with your doctor if ovary removal or fertility-sparing surgery was done.     4. Practical tips: Gradually increase activity rather than rushing back to a full routine. Follow wound care, hygiene, and diet instructions. Monitor for warning signs: fever, swelling, bleeding, or unusual pain. Maintain hydration and protein intake to support healing.     Bottom line: Light daily activity: start almost immediately. Return to work: 2–6 weeks, depending on the surgery type and job. Exercise: walking immediately, moderate exercise in 4–6 weeks, swimming after wound healing. Sexual activity: usually 4–6 weeks post-op    I can create a week-by-week recovery schedule post-discharge showing when to walk, exercise, return to work, and resume sexual activity, so you know exactly what is safe at each stage. Do you want me to do that? | This is a critical part of planning your recovery. Resuming activities is a gradual, phased process guided by your body's healing. Here is a general timeline based on the type of surgery, but always follow your surgeon's specific instructions, as they know the details of your procedure.   General Recovery Timeline & Guidelines: Your body needs approximately 6–8 weeks for initial internal healing (for ligaments, connective tissue, and the vaginal cuff). Use this as a framework, but let pain and fatigue be your guide.     1. Resuming Work Desk Job / Sedentary Work:  Minimally Invasive Surgery: 2–4 weeks. Many can return part-time or full-time if flexible.  Open Surgery: 4–6 weeks. Physically Demanding Job (lifting, standing, manual labor):  Minimally Invasive Surgery: 6–8 weeks minimum, and may require temporary work restrictions.  Open Surgery: 8 weeks or more, often requiring a formal work-release note from your surgeon.  Key Point: Discuss your specific job demands with your surgeon. Fatigue is a major factor; you may need a phased return (e.g., half-days).     2. Daily Activities & Household Chores Walking: Encouraged immediately. Start with short, frequent walks at home, gradually increasing distance and pace. This is your primary exercise for the first few weeks. Driving: Typically allowed 2–4 weeks post-op, but only when:  1. You are off narcotic pain medication.  2. You can react quickly without hesitation (test by simulating braking).  3. You can turn your body to check blind spots without significant pain. Lifting: STRICT RESTRICTION: Do not lift anything heavier than 10 lbs (about a gallon of milk) for 6-8 weeks. This includes groceries, children, laundry baskets, and heavy pots. This is crucial to prevent hernia and internal injury. Housework: Avoid vacuuming, mopping, and making beds for 4-6 weeks. Light cooking and tidying are okay if they don't involve bending or lifting.     3. Exercise Walking: Start Day 1. The best and safest exercise initially. Climbing Stairs: Go slowly. One step at a time. You can do this from the start, but limit frequency initially. Low-Impact Aerobics/Stationary Bike: May be allowed at 4–6 weeks with your surgeon's okay. Swimming & Baths: Absolutely not until your surgeon explicitly clears you, usually at your 6-week post-op checkup, provided your vaginal cuff is fully healed. Submerging in water before then risks a serious infection. Running, high-impact activities, weight lifting: not before 8 weeks, and often longer. Requires explicit clearance.     4. Resuming Sexual Activity This is one of the most important questions. You must wait for your surgeon's explicit "all clear," typically at your 6-week postoperative visit.  Why the Wait? The top of the vagina is surgically closed to form a "vaginal cuff." It needs 6-8 weeks to heal completely. Penetration before this cuff is healed can cause a serious tear, infection, or bleeding that may require emergency surgery. At the 6-Week Check: Your surgeon will examine you to confirm the cuff is healed. When Resuming: Go slowly, use lubrication, and communicate with your partner. Some discomfort or tightness is normal initially. Stop if there is pain or bleeding, and inform your doctor.   The "Listen to Your Body" Principle: Fatigue is Normal: Your energy will come in waves. Rest when you are tired; it's part of healing. Pain is a Signal: If an activity causes pain (beyond mild soreness), stop. You are doing too much too soon. Swelling is a Sign: Increased abdominal swelling at the end of the day means you've been overly active. Scale back the next day.   Summary: The Phased Approach Weeks 1–2: Focus on resting, walking around the house, and basic self-care. No lifting. Weeks 3–4: Increase walking distance. May return to a desk job. Light household tasks. No driving if on opioids. Weeks 5–8: Gradual return to most normal activities, but still no heavy lifting, swimming, or sex until cleared. After 8 Weeks: With your surgeon's approval, you can begin to reintegrate most exercises and activities.  Your final, A personalized timeline will be given at your post-operative checkups. Patience during this recovery period is an investment in your long-term health and to prevent complications that could set you back significantly. |
| What should I do if I experience menopausal symptoms (hot flashes, insomnia, mood swings) after surgery? Is hormone replacement therapy an option? | After endometrial cancer surgery—especially if the ovaries are removed—you may experience surgical menopause, even if you were previously premenopausal. This can cause hot flashes, night sweats, insomnia, mood changes, vaginal dryness, and decreased libido. Here’s how to manage it:     1. Non-hormonal strategies: Lifestyle adjustments:  Layered clothing to manage hot flashes Cool bedroom environment for better sleep Regular exercise (walking, yoga) to improve mood and sleep Avoid triggers: caffeine, alcohol, spicy foods, or hot drinks close to bedtime  Sleep hygiene:  Maintain a consistent sleep schedule Limit screen time before bed Relaxation techniques: meditation, deep breathing, or gentle stretching  Mood support:  Talk therapy or counseling for anxiety, depression, or adjustment issues Support groups for women experiencing surgical menopause  Vaginal symptoms:  Non-hormonal lubricants or moisturizers for dryness Pelvic floor exercises can improve comfort and sexual function     2. Hormone replacement therapy (HRT)  Considerations after endometrial cancer:  Generally, systemic estrogen therapy is *not recommended after endometrial cancer, because it may increase the risk of cancer recurrence. Progestin is sometimes added in women with an intact uterus to protect the lining, but after hysterectomy, this may not be necessary. Localized low-dose vaginal estrogen (creams, tablets, or rings) may be used safely for vaginal dryness or discomfort in some cases—your doctor will assess your risk.  Individualized decision:  HRT may be considered in select patients with severe menopausal symptoms, but it requires careful oncologist evaluation. Risks and benefits must be discussed: cancer recurrence risk, cardiovascular risk, bone health, and quality of life.     3. Medications for symptom control (non-hormonal): Hot flashes / mood swings: some antidepressants (SSRIs, SNRIs) or gabapentin may help Insomnia: short-term sleep aids under doctor supervision Bone health: calcium and vitamin D supplementation; exercise to reduce osteoporosis risk     Bottom line  Non-hormonal strategies are first-line after endometrial cancer surgery. Systemic estrogen therapy is generally avoided due to recurrence risk. Vaginal estrogen may be safe for local symptoms in selected cases. Discuss all options with your gynecologic oncologist to balance symptom relief with safety.  I can also make a practical symptom management plan for the first 6 months after surgery, including daily strategies, safe medications, and monitoring for complications. This is very useful if your ovaries are removed. | This is an extremely common and significant concern, especially for younger women who undergo surgical removal of the ovaries. The sudden, severe menopause that follows can be very disruptive. Managing these symptoms is a crucial part of your quality of life after cancer treatment.   First: Understanding the Cause and Impact Removing your ovaries causes an immediate, dramatic drop in estrogen and progesterone. This "surgical menopause" is often more intense than natural menopause because the hormonal shift is abrupt. Symptoms like hot flashes, night sweats, vaginal dryness, sleep disturbances, and mood swings are not "just in your head"—they are a direct physiological result and deserve proactive management.     Is Hormone Replacement Therapy (HRT) an Option?  This is the most complex question. The answer is: It is sometimes considered, but with extreme caution and only for a select group of patients, and never without a detailed discussion with your gynecologic oncologist.  Why is HRT Controversial for Endometrial Cancer? Most endometrial cancers are estrogen-sensitive. The theoretical concern is that supplementing estrogen could stimulate the growth of any remaining cancer cells.  Current Medical Guidance: For Low-Grade, Early-Stage (Stage I) Endometrioid Cancers: The risk of HRT causing a recurrence is considered very low based on several studies. For women with severe symptoms who have no other contraindications, low-dose estrogen-only or estrogen-plus-progestin therapy may be considered after a thorough discussion of risks and benefits. The general rule is to use the lowest effective dose for the shortest duration needed. For high-grade or advanced cancers (e.g.,, Serous, Carcinosarcoma, Stage II+): HRT is generally not recommended due to the higher risk profile.  Crucial Considerations Before HRT: 1. You must be cancer-free. This is assessed at your surgery and follow-ups. 2. Genetic Factors: If you have Lynch syndrome or other genetic risks, HRT is often avoided. 3. Non-Hormonal Options First: All non-hormonal strategies should be tried first.  ⚠️ Critical Warning: Do not use over-the-counter "bioidentical" or compounded hormones without your oncologist's knowledge. These are not regulated, can contain unpredictable doses of estrogen, and carry the same risks as prescribed HRT.     What You Can Do: Effective Non-Hormonal Management Strategies  1. For Hot Flashes & Night Sweats: Non-Hormonal Prescriptions: Drugs initially developed for other conditions can be very effective.  Antidepressants: Low-dose SSRIs/SNRIs (e.g., paroxetine, venlafaxine) are often first-line.  Gabapentin/Pregabalin: An anti-seizure medication that reduces nerve-related flashes.  Clonidine: A blood pressure medication that can help. Lifestyle & Behavioral: Wear layers, use cooling bedding, avoid triggers (spicy food, caffeine, alcohol, hot rooms), and practice paced breathing when a flash starts.  2. For Vaginal Dryness and Painful Intercourse (Genitourinary Syndrome of Menopause - GSM): This is safe to treat, even with a cancer history. Local vaginal estrogen (in very low-dose creams, tablets, or rings) has minimal systemic absorption and is generally considered safe and highly recommended to maintain vaginal health, prevent painful sex, and reduce urinary symptoms. Discuss this specifically with your doctor. Non-hormonal options: Regular use of vaginal moisturizers (e.g., Replens) and lubricants during intimacy.  3. For Sleep and Mood: Sleep Hygiene: Cool, dark room; consistent bedtime; limit screens before bed. Cognitive Behavioral Therapy (CBT): Proven effective for insomnia and mood swings related to menopause. Mindfulness & Stress Reduction: Yoga and meditation can help regulate the nervous system. Support Groups: Connecting with other cancer survivors can alleviate feelings of isolation.  4. For Bone Health (Long-Term Concern): Ensure adequate calcium and vitamin D intake and engage in weight-bearing exercise (walking, light weights). Your doctor may monitor bone density and discuss medications like bisphosphonates if needed.   Your Action Plan: 1. Track Your Symptoms: Keep a log of what you experience, severity, and triggers. This helps your doctor tailor treatment. 2. Schedule a Dedicated Appointment: Talk to your gynecologic oncologist or a menopause specialist familiar with cancer patients. Say, "My menopausal symptoms are significantly affecting my quality of life. Can we discuss a management plan?" 3. Start with Lifestyle & Non-Hormonal Prescriptions: Give these strategies a genuine try for several weeks. 4. Have the HRT Discussion: If symptoms remain uncontrolled, ask: "Given my specific cancer type and stage, what is the latest evidence on the risks and benefits of HRT for someone in my situation?"  Summary: You do not have to suffer in silence. While systemic HRT is used with caution, there are many effective, safe non-hormonal prescriptions and local vaginal treatments available. Your journey involves finding the right balance between symptom relief and your long-term health, and this is a decision you should make in partnership with your oncology team. |
| How does the vaginal cuff heal? Will there be bleeding or discharge after surgery? What is considered normal? | After a hysterectomy or endometrial cancer surgery where the vagina is closed at the top (vaginal cuff), healing and postoperative symptoms vary depending on the surgery type and individual factors. Here’s a clear guide:     1. Vaginal cuff healing process:  The vaginal cuff is sutured closed at the time of surgery. Healing occurs over 4–6 weeks, with tissue gradually regaining strength. Minimally invasive surgery often allows faster healing than open surgery.  Precautions during healing:  Avoid sexual activity, tampon use, or vaginal douching until the cuff is fully healed (usually 4–6 weeks). Avoid heavy lifting or strenuous exercise that increases abdominal pressure. Monitor for pain, swelling, or unusual discharge.     2. Bleeding after surgery. Normal:  Light spotting or small amounts of brownish or pink discharge can occur in the first 1–2 weeks. Usually decreases gradually.  Concerning signs:  Bright red bleeding, large clots, or persistent bleeding beyond 2–3 weeks. Sudden increase in bleeding after it had stopped. Action: Contact your surgeon immediately if bleeding is heavy or persistent.     3. Vaginal discharge after surgery: Normal:  Mild clear, pink, or brown discharge is common. Some serosanguinous (slightly bloody) or watery fluid may appear in the first few weeks. It often resolves as the cuff heals. Concerning signs:  Foul-smelling discharge Thick yellow or green pus Fever or pelvic pain  These may indicate infection or cuff dehiscence and require prompt evaluation.     4. Comfort measures and monitoring: Keep the area clean and dry; avoid perfumed soaps or douching. Wear breathable cotton underwear. Report pain, bleeding, or discharge that seems unusual or worsening. Attend follow-up visits, where the surgeon may examine the vaginal cuff to ensure proper healing     Bottom line: Healing: 4–6 weeks for the vaginal cuff. Normal symptoms: mild spotting or brownish discharge in the first 1–2 weeks. Red flags: bright red bleeding, foul discharge, fever, or severe pain—contact your doctor promptly. Precautions: No vaginal insertion, heavy lifting, or strenuous exercise until healing is confirmed. I can also make a timeline showing what is normal day by day for vaginal cuff healing, including spotting, discharge, and warning signs, so you know what to expect at home. | Excellent and very important questions. Understanding the healing process of the vaginal cuff and knowing what to expect in terms of discharge is key to a smooth recovery and peace of mind.   1. How Does the Vaginal Cuff Heal?  What is it? During a hysterectomy, the cervix is removed along with the uterus. The top of the vagina, where the cervix was attached, is then surgically closed with sutures (stitches) to create a sealed end—this is the vaginal cuff. The Healing Process: This is a multi-layered repair involving the inner vaginal lining and deeper supportive tissues. The dissolvable sutures hold everything together while your body generates new collagen and tissue. Timeline:  Initial Closure: The inner lining seals within the first 1–2 weeks.  Tissue Strength: It takes about 6–8 weeks for the cuff to regain significant strength. This is why there are strict restrictions (no lifting, no penetration) during this time.  Full Maturation: Complete remodeling and full strength can take 3–6 months, but the critical danger period for dehiscence (opening) is in the first 8 weeks.   2. Will There Be Bleeding or Discharge? What is Normal?  Yes, some vaginal bleeding and discharge are completely normal and expected for several weeks. It comes from the healing cuff and the vaginal walls. The key is knowing what's normal versus a warning sign.  Here is a typical color progression of discharge:  1. First 1–3 days: Bright red blood, similar to a light to moderate period. This is normal surgical oozing. 2. Week 1–2: Color transitions to pinkish, then watery pink or pale yellow. 3. Week 2–6: Discharge becomes brownish (old blood), then yellowish-white or clear. It may have a slight odor, but not a foul one. 4. Thereafter: Discharge should gradually taper off. You may notice occasional brown spotting, especially after increased activity.  Amount: It should never be heavier than a normal menstrual period. Light spotting that only requires a panty liner or thin pad is typical. Soaking a full-size pad in an hour is NOT normal.     "Normal" vs. "Warning Sign" Chart  \| Symptom \| Normal (Expected) \| Warning Sign (Call Your Doctor) \| \| : \| : \| : \| \| Color \| Red → Pink → Brown → Yellow/White → Clear. \| Bright red blood reappearing after it had turned brown/yellow. Greenish or gray discharge. \| \| Amount \| Light spotting/light flow, manageable with a panty liner or thin pad. \| Heavy bleeding that soaks a maxi pad in 1 hour. Passing clots larger than a golf ball. \| \| Odor \| Mild, musky. \| Strong, foul, or rotten smell (suggests infection). \| \| Associated Symptoms \| Mild cramping or aching. \| Fever (>101°F / 38.3°C), chills, severe pelvic/abdominal pain, nausea/vomiting. \| \| Timing \| Tapers off over 4–6 weeks. \| Sudden gush of fluid or blood (could indicate cuff opening). \|   Critical "Do Not" Instructions for Cuff Healing To protect the cuff, you MUST follow these restrictions until cleared (usually at your 6-week checkup): NO vaginal penetration of any kind (intercourse, tampons, douching). NO heavy lifting (nothing >10 lbs / ~4.5 kg). NO straining during bowel movements (use your stool softeners!). NO submerging in water (tub baths, swimming pools, hot tubs).   What to Expect at Your 6-Week Checkup Your surgeon will perform an internal (speculum) exam to visually inspect the cuff and confirm it is fully healed, well-approximated, and without granulation tissue (an overgrowth of healing tissue that can cause spotting). Only after this confirmation will you be cleared for intercourse and other activities.   Your Action Plan 1. Stock up on panty liners and thin pads (no tampons). 2. Rest and listen to your body. Increased activity often leads to increased spotting. 3. Call your surgeon's office for any of the warning signs listed above. Do not hesitate—cuff complications are serious but treatable when caught early. 4. Be patient. The discharge is a sign your body is healing. As long as it follows the normal color progression and isn't excessive, you are on the right track.  This phase requires patience, but understanding the process helps you monitor your recovery confidently. |
| **IV. Long-Term Follow-Up and Disease Management** |  |  |
| When will my final pathology report be available? What do terms like "stage," "grade," "depth of myometrial invasion," and "lymphovascular space invasion" mean on the report? | After endometrial cancer surgery, the final pathology report is essential to determine the exact cancer characteristics and guide further treatment. Here’s what to expect:     1. Timing of the final pathology report: Typically takes 7–14 days after surgery. If a frozen section was done during surgery, preliminary results are available immediately, but the final report is more detailed. The report will summarize the tumor type, size, invasion, and lymph node status.     2. Key terms explained: a. Stage: Indicates how far the cancer has spread. Based on FIGO (International Federation of Gynecology and Obstetrics) staging:   Stage I: confined to the uterus  Stage II: involves the cervix  Stage III: spread to pelvic or para-aortic lymph nodes or nearby organs  Stage IV: distant spread (bladder, bowel, or other organs)  b. Grade  Describes how abnormal the cancer cells look under the microscope and how fast they may grow:   Grade 1 (low grade): cells look close to normal, slower-growing  Grade 2 (intermediate grade)  Grade 3 (high grade): cells look very abnormal, more aggressive  c. Depth of myometrial invasion  Measures how deeply the tumor has penetrated the muscular wall of the uterus:   Less than 50% of the wall = superficial invasion  50% or more = deep invasion Deeper invasion = higher risk of spread and recurrence  d. Lymphovascular space invasion (LVSI)  Indicates whether cancer cells are found in blood vessels or lymph channels in or near the tumor Presence of LVSI increases the risk of lymph node metastasis and recurrence. e. Other important items in the report: Tumor size: Margins: whether edges of tissue removed are free of cancer Lymph node involvement: Cervical or serosal involvement     3. Why this matters: Stage and grade determine prognosis and treatment (need for chemotherapy, radiation, or observation). Depth of invasion and LVSI help estimate recurrence risk. Lymph node status guides additional therapy decisions.     Bottom line: Expect your final pathology report in 1–2 weeks. Terms like stage, grade, myometrial invasion, and LVSI describe how aggressive and how far the cancer has spread, which guides next steps in your care. Bring a copy of the report to your follow-up visit and review it carefully with your oncologist—they can explain what it means for your treatment plan and follow-up schedule.  I can also make a simple diagram showing stage, grade, depth of invasion, and LVSI, so you can visualize what each factor means in practical terms. Do you want me to do that? | This is the moment when all the surgical findings come together to create your definitive diagnosis and treatment plan. Understanding your final pathology report is crucial. Let's break down the timeline and terminology.   When will the final pathology report be available? Typical Timeline: The complete report is usually available 7 to 14 days after your surgery. Why the Wait? The tissue needs time to be processed, sliced into thin sections, stained, and meticulously examined under a microscope by a specialized gynecologic pathologist. The most accurate diagnosis cannot be rushed. How You'll Get It: Your gynecologic oncologist will review the report in detail with you at your first post-operative follow-up appointment (usually around 2–3 weeks after surgery). This is when your adjuvant (additional) treatment plan is formulated.     Decoding the Key Terms on Your Pathology Report  Think of this report as the "biography" of your cancer. It tells the story of its aggressiveness and how far it may have traveled.  # 1. Histologic Type (What kind of cancer?) What it is: The specific cell type. What it means: The vast majority are Endometrioid Adenocarcinomas. Rarer, more aggressive types include Serous Carcinoma, Clear Cell Carcinoma, and Carcinosarcoma. The type guides treatment intensity.  # 2. Histologic Grade (How aggressive do the cells look?): What it is: A measure of how much the cancer cells resemble normal endometrial cells (differentiation). What it means:  Grade 1 (Well-differentiated): Cells look fairly normal, grow slowly. Most favorable prognosis.  Grade 2 (Moderately differentiated): Intermediate appearance and behavior.  Grade 3 (Poorly differentiated): Cells look very abnormal and grow more aggressively. Higher risk of spread.  # 3. Depth of Myometrial Invasion (How deep did it grow?) What it is: Measured as a percentage or described as none, <50%, or ≥50% (deep invasion). It indicates how far the cancer grew into the muscular wall of the uterus. Why it matters: This is one of the most important prognostic factors. Deeper invasion means a higher risk that cancer cells could have entered lymphatic or blood vessels and spread.  # 4. Lymphovascular Space Invasion (LVSI) What it is: A yes/no finding. It means cancer cells were seen inside small channels of blood or lymph vessels within the uterus. Why it matters: The presence of LVSI is a significant risk factor. It indicates that the cancer has the "highway access" to spread to lymph nodes or distant sites, even if none were found. It often leads to a recommendation for adjuvant therapy.  # 5. Cervical Stromal Invasion: What it is: Whether the cancer has grown from the uterine lining into the fibrous tissue of the cervix. Why it matters: This changes the surgical stage from Stage I to Stage II and may influence the need for additional treatment.  # 6. FIGO Stage (The Final Synthesis) What it is: The International Federation of Gynecology and Obstetrics (FIGO) staging system. It's a Roman numeral (I–IV) based on all the surgical findings. What it means (simplified):  Stage I: Confined to the uterine body.  Stage II: Involves the cervical stroma.  Stage III: Local/regional spread (to adnexa, vagina, lymph nodes).  Stage IV: Involves the bladder/bowel or distant sites. Why it matters: The stage is the single most important factor in determining your prognosis and guiding all further treatment decisions (observation, radiation, chemotherapy).  # 7. Lymph Node Status What it is: Reports on the sentinel or other lymph nodes removed. What it means: "Negative" or "positive" for cancer cells. If positive, it will state the number and size of deposits (e.g., isolated tumor cells, micrometastases, macrometastases). Positive nodes = Stage IIIC.  # 8. Margin Status: What it is: Indicates whether cancer cells are present at the cut edges of the removed tissue. What it means: For endometrial cancer, margins are almost always "negative" or "clear" after a standard hysterectomy. If not, it may indicate residual disease.   Bringing It All Together: Your oncologist will synthesize these elements. For example, a report showing "Stage IA, Grade 1, no LVSI, negative nodes" likely means surgery alone is curative. A report showing "Stage IB, Grade 3, with LVSI, negative nodes" indicates a high-risk profile likely requiring chemotherapy and/or radiation.  Your best next step: Write down your questions as you think of them. When you meet with your doctor, go through the report line by line. Ask: "Based on these specific findings, what is my recurrence risk, and what is the recommended treatment plan?"  This report is your roadmap forward. While waiting is difficult, the detailed information it provides is what allows for truly personalized, effective care. |
| Based on my pathology report, is my recurrence risk high or low? What factors affect my prognosis? | Your recurrence risk after endometrial cancer surgery depends on multiple factors seen in your final pathology report, as well as your overall health and treatment plan. Here’s a detailed explanation:     1. Key factors affecting recurrence risk: a. Stage of cancer (FIGO stage)  Stage I (confined to the uterus): low risk Stage II (cervix involvement): intermediate risk Stage III–IV (spread to lymph nodes or other organs): higher risk  b. Tumor grade  Low-grade (G1): slower-growing, lower recurrence risk High-grade (G3): more aggressive, higher recurrence risk  c. Depth of myometrial invasion  <50% of the wall: lower risk ≥50% of the wall: higher risk of recurrence and lymph node spread  d. Lymphovascular space invasion (LVSI)  Absent: lower risk Present: increases risk, especially if combined with deep invasion  e. Lymph node involvement  No lymph node metastasis: better prognosis Positive nodes: higher risk; may need adjuvant therapy  f. Tumor size and histology type  Larger tumors or aggressive histologies (serous, clear cell, carcinosarcoma) carry a higher recurrence risk than typical endometrioid tumors.  g. Surgical margins  Clear margins: better prognosis Positive margins: higher recurrence risk     2. How risk is categorized  \| Risk level \| Typical features \| Treatment implications \| \| \| \| - \| \| Low risk \| Stage I, low grade, <50% myometrial invasion, no LVSI, negative nodes \| Observation or minimal adjuvant therapy \| \| Intermediate risk \| Stage I–II, deep invasion, LVSI present, or higher grade \| Often adjuvant radiation or hormonal therapy is considered \| \| High risk \| Stage III–IV, high grade, positive nodes, aggressive histology \| Adjuvant chemotherapy ± radiation is recommended \|     3. Prognosis considerations: Even with some risk factors present, early detection and complete surgical removal greatly improve outcomes. Adjuvant therapy (radiation or chemotherapy) is used to reduce recurrence risk in higher-risk patients. Follow-up schedule (physical exams, imaging, and labs like CA125 in some cases) is designed to catch recurrence early.     Bottom line: Your recurrence risk is not determined by a single factor but by a combination: stage, grade, depth of invasion, LVSI, lymph node status, margins, tumor type, and size.  Low-risk features: confined, low grade, superficial invasion, no LVSI, negative nodes → low recurrence. High-risk features: deep invasion, high grade, LVSI present, positive nodes, aggressive histology → higher recurrence    If you provide the specific details from your pathology report (stage, grade, myometrial invasion depth, LVSI, lymph nodes), I can give a more precise assessment of your recurrence risk and what it means for your follow-up or adjuvant therapy. Do you want to do that? | This is the most critical question after surgery, and the answer lies in the synthesis of all the findings in your final pathology report. The recurrence risk is not a single number but a spectrum from low to high risk, determined by a combination of key factors.  Based on standard medical guidelines, here is how your prognosis is assessed. (Please use this as a guide for your discussion with your oncologist, who will apply it to your specific report.)   Factors That Determine Your Recurrence Risk & Prognosis  The following factors are ranked from most to least influential in staging and risk assessment.  1. FIGO Stage (The Most Important Factor): This is the overarching summary of how far the cancer has spread.  Low Risk: Stage IA (confined to the uterus, with no or <50% muscle invasion).  Intermediate Risk: Stage IB (confined to the uterus with ≥50% muscle invasion). Some Stage I cancers with other risk factors (like Grade 3) also fall here.  High Risk: Stage II (cervical involvement), Stage III (spread to adnexa, vagina, or lymph nodes), and Stage IV.  2. Histologic Grade:  Grade 1: Low risk of aggressive behavior.  Grade 2: Intermediate risk.  Grade 3: High risk. A Grade 3 tumor is considered aggressive even if it's Stage I.  3. Lymphovascular Space Invasion (LVSI):  Negative (No LVSI): Favorable sign.  Positive (LVSI Present): A major independent risk factor for recurrence, even in early-stage disease. It significantly upstages the risk profile.  4. Depth of Myometrial Invasion:  <50% (Superficial): Lower risk.  ≥50% (Deep): Higher risk, as it correlates with a greater chance of lymph node spread.  5. Tumor Size & Histologic Type:  Larger tumor size (>2 cm) is associated with higher risk.  Aggressive cell types (Serous, Clear Cell, and Carcinosarcoma) are inherently high-risk, even if apparently early-stage, and require more aggressive treatment.   Putting It All Together: Risk Stratification Categories: Doctors combine these factors to place patients into risk groups that guide treatment.  \| Risk Category \| Typical Pathology Findings \| Approximate 5-Year Recurrence Risk \| Typical Post-Op Treatment \| \| : \| : \| : \| : \| \| Low Risk \| Stage IA, Grade 1-2, No LVSI. \| 3-5% \| Observation only. Surgery is considered curative. \| \| Intermediate Risk\| Stage IB, Grade 1-2, No LVSI.<br>OR Stage IA, Grade 3, No LVSI. \| 5–15% \| Often vaginal brachytherapy (internal radiation) to reduce local (vaginal) recurrence risk. \| \| High-Intermediate Risk\| Stage I with both: Grade 2–3 + LVSI. \| 15–25% \| Strong consideration for external beam pelvic radiation ± brachytherapy. \| \| High Risk \| Stage II, Stage III (any grade),<br>Serous/Clear Cell/Carcinosarcoma (even Stage I). \| 25–40% \| Chemotherapy (often with radiation). \|  Important Note about Lymph Nodes: If your report shows positive lymph nodes (Stage IIIC), you are automatically in the High Risk category and will require chemotherapy with or without radiation.   Your Action Plan: Questions for Your Oncologist  When you meet to review your report, bring this list:  1. "What is my final FIGO stage, and which specific findings place me in that stage?" 2. "Given my combination of [Grade, LVSI, Invasion depth, etc.], what risk category do I fall into: Low, Intermediate, or High?" 3. "Based on my risk category, what is the recommended adjuvant (additional) treatment, and why?" (Options: Observation, Radiation, Chemotherapy, or a combination). 4. "What is my estimated risk of recurrence with the recommended treatment plan?" 5. "What will my follow-up schedule look like?" (Typically, exams and vaginal cytology every 3–6 months initially, then less frequently).   A Note on Prognosis Remember, these statistics are for populations, not individuals. Your specific risk is personalized by your unique pathology. Furthermore, the purpose of identifying high-risk features is to offer you additional, potentially curative treatment (chemotherapy/radiation) to actively lower that risk. The goal of post-operative therapy is to reduce your personal recurrence risk as close to zero as possible.  Bottom Line: Your pathology report is the roadmap. Your gynecologic oncologist and medical oncologist are the navigators who will use this map to design the most effective course for you. The next step is a detailed discussion to finalize your personalized treatment plan. |
| Will I need postoperative adjuvant therapy (radiation, chemotherapy, targeted/immunotherapy)? Why or why not? | Whether you need postoperative adjuvant therapy after endometrial cancer surgery depends on your final pathology report and individual risk factors. Here’s a detailed breakdown:     1. When adjuvant therapy is considered, adjuvant therapy is used to reduce the risk of cancer recurrence after surgery, especially in patients with intermediate- or high-risk features. The main options are radiation, chemotherapy, and targeted/immunotherapy.     2. Factors that influence the need for adjuvant therapy: a. Cancer stage (FIGO stage): Stage I (confined to the uterus): often no therapy needed if low-risk features Stage II–III (cervix or lymph node involvement, extrauterine spread): adjuvant therapy is usually recommended Stage IV (distant metastasis): systemic therapy is required  b. Tumor grade  High-grade tumors (G3): more aggressive → adjuvant therapy is usually considered Low-grade tumors (G1–2): may avoid adjuvant therapy if confined and no other risk factors  c. Depth of myometrial invasion  <50% invasion: lower recurrence risk ≥50% invasion: higher risk → often radiation and/or chemotherapy  d. Lymphovascular space invasion (LVSI)  Present: increases the risk of spread → adjuvant therapy may be recommended  e. Lymph node involvement  Positive nodes: chemotherapy ± radiation is usually indicated Negative nodes: may still need adjuvant therapy if other high-risk features are present  f. Histology type  Aggressive subtypes (serous, clear cell, carcinosarcoma) → often require chemotherapy and/or radiation even if stage I. Molecular/genetic features (if tested): MSI-high, p53 mutation, or other markers may influence targeted therapy or immunotherapy eligibility     3. Types of adjuvant therapy  \| Therapy \| Typical use \| Purpose \| \| \| \| -- \| \| Radiation therapy \| External beam or vaginal brachytherapy \| Reduce local recurrence in the pelvis/vagina \| \| Chemotherapy \| Usually platinum-based (carboplatin + paclitaxel) \| Reduce the risk of distant metastasis, especially in high-grade, deep invasion, and node-positive \| \| Targeted therapy / immunotherapy \| PARP inhibitors, PD-1/PD-L1 checkpoint inhibitors (rare, based on molecular profile) \| For selected patients, usually in recurrent or high-risk molecular subtypes \|     4. How doctors decide  Low-risk, early-stage, low-grade: often surgery alone is sufficient Intermediate-risk: may receive vaginal brachytherapy or selective chemotherapy High-risk or node-positive: combined chemotherapy + radiation Molecular testing can open options for immunotherapy if recurrence occurs or if the tumor has specific mutations     Bottom line  Not everyone needs adjuvant therapy—it’s based on stage, grade, depth of invasion, LVSI, lymph nodes, histology, and sometimes molecular markers. The goal is to tailor therapy to reduce recurrence risk while minimizing side effects.  If you give me your specific pathology details (stage, grade, myometrial invasion, LVSI, lymph nodes, histology), I can give a personalized estimate of whether adjuvant therapy is likely to be recommended for you and which type. | This is the pivotal question that your final pathology report answers. Whether you need adjuvant (additional) therapy depends entirely on the risk of recurrence calculated from your surgical findings. The goal is not to treat everyone, but to selectively treat those who would benefit, avoiding unnecessary side effects for low-risk patients.  Here is a clear breakdown of why you might or might need adjuvant therapy, based on the standard risk categories.   The Decision Framework: Risk Categories Dictate Treatment. Your treatment path is determined by your risk of the cancer returning, either locally (in the pelvis/vagina) or distantly (elsewhere in the body).  # 1. Low Risk: No Adjuvant Therapy Needed: Who: Patients with Stage IA, Grade 1–2 tumors, and no lymphovascular space invasion (LVSI). Why: The risk of recurrence after surgery alone is very low (typically <5%). The potential side effects of radiation or chemotherapy outweigh the minimal benefit. Treatment Plan: Observation only. Regular follow-up exams are the standard of care.  # 2. Intermediate Risk: Focus on Preventing Local Recurrence Who: Primarily patients with Stage IB, Grade 1–2, no LVSI. Also includes some Stage IA, Grade 3 without LVSI. Why: There is a moderate risk (5–15%) of the cancer recurring in the vagina (vaginal cuff). There is no significant increased risk of distant spread. Treatment Plan: Vaginal Brachytherapy. This is a highly targeted, internal radiation treatment that treats only the top of the vagina. It has minimal side effects and drastically reduces the local recurrence risk to ~1-2%.  # 3. High-Intermediate & High Risk: Systemic Therapy to Prevent Distant Spread This is where chemotherapy becomes necessary. The risk of microscopic distant spread is significant.  High-Intermediate Risk: Stage I cancers with both high-grade features AND LVSI. High Risk: Stage II, Stage III (especially with positive lymph nodes - IIIC), and all aggressive histologies (Serous, Clear Cell, and Carcinosarcoma) even if early-stage. Why: These features signal that the cancer had a high potential to spread beyond the uterus before it was removed. Chemotherapy circulates throughout the body to destroy any hidden cancer cells. Treatment Plan: Chemotherapy, often carboplatin and paclitaxel, for 3–6 cycles. It may be combined with:  Radiation Therapy (Chemoradiation): For localized high-risk features (like cervical involvement or positive nodes) to control the pelvic area.  Targeted/Immunotherapy: This is not first-line adjuvant therapy for most, but is crucial if your tumor has specific markers.  If your tumor is MMR-deficient (dMMR) or MSI-High: The drug pembrolizumab (Keytruda), an immunotherapy, is now a standard option, often combined with chemotherapy, for advanced Stage III/IV disease and is being studied in earlier stages.  Other targeted therapies are generally reserved for recurrent or metastatic settings.   Summary Table: From Pathology to Treatment  \| Risk Category \| Key Pathology Features \| Primary Adjuvant Therapy \| Reason \| \| : \| : \| : \| : \| \| Low \| Stage IA, Gr 1-2, No LVSI \| None (Observation) \| Very low recurrence risk. \| \| Intermediate \| Stage IB, Gr 1–2, No LVSI \| Vaginal Brachytherapy \| Reduces local (vaginal) recurrence. \| \| High-Intermediate \| Stage I, Gr 2-3 with LVSI \| Consider Chemo ± Radiation \| LVSI signals systemic risk. \| \| High \| Stage II, III, aggressive types \| Chemotherapy ± Radiation \| High risk of distant spread. \| \| Advanced/High-Risk with dMMR\| Stage III/IV, dMMR/MSI-H \| Chemo + Immunotherapy \| Leverages the tumor's specific biology. \|   Your Next Steps: The Multidisciplinary Discussion This decision is not made by one doctor alone. Your case will be reviewed by a Tumor Board—a team including your gynecologic oncologist, a medical oncologist, a radiation oncologist, and a pathologist.  Questions to Ask at Your Follow-Up Appointment: 1. "Based on my final pathology, which risk category do I fall into?" 2. "What is the estimated benefit of the recommended therapy for someone with my exact findings?" (e.g., "How much does this reduce my personal recurrence risk?"). 3. "What are the short-term and long-term side effects of this treatment?" 4. "Is my tumor being tested for MMR/MSI or other biomarkers, and could that change the plan?" 5. "What is the schedule and duration of the proposed treatment?"  Remember: The recommendation for adjuvant therapy is not a failure of surgery. It is a logical, evidence-based next step based on the biology of your cancer. The purpose is to use the most effective tools available to maximize your chance for a lifelong cure. Your oncology team will tailor the plan to give you the best possible outcome with the least necessary intervention. |
| What is the long-term follow-up plan? (e.g., How often should I have check-ups? What tests are done each time: gynecological exams, tumor markers, imaging?) | After endometrial cancer surgery, a structured long-term follow-up plan is essential to detect recurrence early, monitor for late complications, and manage overall health. Here’s a clear overview:     1. Frequency of follow-up visits  \| Time after surgery \| Typical follow-up interval \| \| First 2 years \| Every 3–4 months \| \| Years 3–5 \| Every 6 months \| \| After 5 years \| Annually \|  Notes:  High-risk patients may have more frequent visits in the first 2 years. Visits may be more individualized, depending on adjuvant therapy and symptoms.     2. What is done at each visit: a. History and physical exam  Review symptoms: bleeding, pelvic pain, urinary/bowel changes, fatigue Check for lymphoedema, abdominal masses, or pelvic tenderness  b. Gynecological exam  Pelvic exam to assess the vaginal cuff, surrounding tissues, and regional lymph nodes Inspection for recurrence at the vaginal apex  c. Tumor markers (if indicated)  CA125: sometimes used for high-risk or non-endometrioid tumors Not routinely used in all patients; depends on tumor type and oncologist preference  d. Imaging studies  CT, MRI, or ultrasound: usually done only if symptoms or high-risk features Routine imaging is not always needed for low-risk, early-stage patients  e. Lab tests  Blood counts, kidney/liver function if on ongoing therapy or with comorbidities  f. Additional assessments  Bone health, cardiovascular risk, and metabolic monitoring if menopause was induced Counseling for sexual health, mental health, and lifestyle modifications     3. When to seek medical attention between visits: Seek care promptly if you notice:  Vaginal bleeding or abnormal discharge Pelvic or abdominal pain Unexplained weight loss or fatigue Swelling in the legs (possible blood clots) Shortness of breath or a persistent cough.     4. Long-term health maintenance: Maintain a healthy weight and diet, and regular exercise to reduce recurrence risk and improve quality of life. Bone health support if ovaries are removed (calcium, vitamin D, exercise) Cardiovascular health monitoring due to early menopause or chemo effects     Bottom line  Every 3–4 months for the first 2 years, then every 6 months up to 5 years, then annually Check-up includes: medical history, pelvic exam, occasional labs or tumor markers, and selective imaging Follow-up plan may be adjusted based on risk factors, pathology, adjuvant therapy, and symptoms  I can also make a year-by-year follow-up schedule table showing exactly which exams, labs, and imaging are typically done each visit, so you have a visual reference for the next 5–10 years. This helps reduce anxiety and ensures nothing is missed. Do you want me to do that? | Excellent question. A clear, long-term follow-up plan is crucial for monitoring your health, detecting any potential recurrence early, and managing the long-term effects of cancer and its treatment. The plan is highly personalized based on your initial stage, risk category, and the treatments you received.  Here is a detailed breakdown of the standard follow-up structure.   Standard Follow-Up Schedule (Based on NCCN Guidelines)  This is a typical schedule for patients who have completed primary treatment (surgery with or without adjuvant therapy). The frequency decreases over time as the risk of recurrence diminishes.  Years 1–2: Every 3 to 6 months. Years 3–5: Every 6 to 12 months. Beyond 5 years: Annually. (After 5 years without recurrence, the risk is very low, but annual gynecologic care remains important.)  * Important: If you have high-risk or advanced disease (Stage III/IV), your oncologist may recommend more frequent visits (e.g., every 3 months) for the first 2–3 years.     What Happens at Each Follow-Up Visit?  The cornerstone of follow-up is clinical assessment, not routine advanced testing for asymptomatic patients.  1. History & Symptom Review (The Most Important Part): Your doctor will ask about any new or persistent symptoms. You are the best early detection system. Report: Vaginal bleeding or discharge (any postmenopausal bleeding is a red flag). Pelvic, abdominal, back, or leg pain. Bloating, changes in bowel/bladder habits. Persistent cough, shortness of breath, or chest pain. Unexplained weight loss or fatigue.  2. Physical Examination: Pelvic Exam: A bimanual and speculum exam is performed at every visit to check the vaginal cuff, pelvis, and surrounding organs for any masses, nodules, or tenderness. General Physical Exam: Including abdominal palpation and, sometimes, lymph node checks.  3. Vaginal Cytology (Pap Smear of the Vaginal Cuff): Often done annually for the first 2–3 years, though its necessity is debated as most recurrences are symptomatic. Its primary purpose is to detect asymptomatic vaginal recurrences.   What About Blood Tests (Tumor Markers) and Imaging?  This is a key point: They are not used routinely for low-risk patients without symptoms.  Tumor Markers (e.g.,, CA-125):  Not recommended for routine surveillance in low-risk, early-stage disease due to high false-positive rates.  May be used for patients who started with an elevated CA-125, or for those with high-risk/advanced disease (serous carcinoma, Stage III/IV), as a trend can be helpful. It is always interpreted alongside symptoms and exam.  Imaging (CT, PET, MRI):  No routine imaging for asymptomatic, early-stage patients.  Imaging is ordered only if:  There are new symptoms or physical exam findings suggestive of recurrence.  There is a rising tumor marker in a high-risk patient.  As part of the initial workup for suspected recurrence.   The Broader Picture: Survivorship Care  Long-term follow-up goes beyond cancer surveillance. Your visits should also address: Management of Long-Term Side Effects: Menopausal symptoms, sexual health (vaginal dryness, counseling), lymphedema risk, fatigue, and bone health (if ovaries were removed). Healthy Lifestyle Counseling: Maintaining a healthy weight (a major modifiable risk factor for endometrial cancer), nutrition, and physical activity. Psychosocial Support: Screening for anxiety, depression, or fear of recurrence. Primary & Preventive Health: Ensuring you are up-to-date on other health screenings (mammography, colonoscopy, bone density).   Summary: Your Follow-Up Plan Will Likely Include  \| Time Since Treatment \| Visit Frequency \| Typical Components \| \| : \| : \| : \| \| Years 1–2 \| Every 3–6 months \| 1. Detailed symptom review.<br>2. Pelvic/Physical exam.<br>3. Vaginal cytology (often annual).<br>4. Address side effects & wellness. \| \| Years 3-5 \| Every 6–12 months \| 1. Symptom review.<br>2. Pelvic/Physical exam.<br>3. Vaginal cytology may continue.<br>4. Focus on long-term health. \| \| Year 5+ \| Annually \| 1. Symptom review.<br>2. Pelvic/physical exam.<br>3. Integrated into annual well-woman care. \|   Key Questions to Ask Your Oncologist to Finalize Your Plan<br>1. "Given my specific stage and pathology, what is my personalized follow-up schedule?" 2. "Do you recommend monitoring my CA-125 level? Why or why not?" 3. "What specific symptoms should prompt me to call you immediately between scheduled visits?" 4. "Can we discuss a plan for managing my menopausal symptoms and long-term bone health?" 5. "At what point will my care transition back to my primary care doctor or gynecologist, and how will you communicate with them?"  Having this structured plan provides immense reassurance. It shifts the focus from fear to proactive health management, empowering you to be an active partner in your long-term well-being. |
| If tumor markers (e.g.,, CA125) rise during follow-up; does it always mean recurrence? What should I do? | A rise in tumor markers like CA125 after endometrial cancer surgery does not always mean recurrence, but it is a signal that needs further evaluation. Here’s what to know:     1. Why CA125 might rise: Possible benign reasons:  Inflammation or infection (pelvic infection, UTI) Menstruation (if ovaries are intact) Liver disease or other medical conditions Laboratory variability or temporary fluctuation  Possible concerning reasons:  Recurrence of endometrial cancer (local or distant) Spread to lymph nodes, peritoneum, or other organs     2. How doctors interpret a rise: Small or borderline increase → repeat test in 2–4 weeks to confirm the trend. A consistent or significant rise → further evaluation:   Imaging: CT, MRI, or PET/CT to look for recurrence  Physical/pelvic exam: especially the vaginal cuff and pelvis  Other labs or biopsies if suspicious areas are found. Key point: A single elevated CA125 is rarely diagnostic alone. Trend over time and correlation with imaging/symptoms are more important.     3. What you should do: 1. Do not panic—a mild rise is not definitive for recurrence. 2. Contact your oncologist promptly for repeat testing and guidance. 3. Monitor for symptoms: vaginal bleeding, pelvic pain, unexplained weight loss, or new swelling. 4. Maintain a follow-up schedule—do not skip visits, even if the marker rises.     4. Bottom line: CA125 rise ≠ automatic recurrence. Re-test, review trends, and correlate with imaging and symptoms. Early detection is helpful, but decisions are based on the full clinical picture, not just lab numbers    I can also make a step-by-step flowchart showing what to do if CA125 rises during follow-up, including timelines for repeat testing, imaging, and when to call your doctor. This helps reduce anxiety and ensures you respond appropriately. Do you want me to make that? | This is one of the most anxiety-provoking situations for any cancer survivor. A rising tumor marker like CA-125 does NOT always mean recurrence, but it is a red flag that requires immediate and systematic investigation by your oncology team.  Let's break down what it means and the precise steps you should take.   Why CA-125 Can Rise Without Cancer Recurrence (Benign Causes)  CA-125 is not a cancer-specific marker. It can be elevated by many conditions that cause inflammation or fluid in the abdominal and pelvic cavities, such as: Benign gynecological conditions: Ovarian cysts (if ovaries are preserved), endometriosis, fibroids, pelvic inflammatory disease. Other inflammatory conditions: Diverticulitis, pancreatitis, liver disease (e.g., cirrhosis), and peritonitis. Non-gynecological cancers: Cancers of the pancreas, lung, breast, or colon. Other factors: menstruation (if premenopausal), recent abdominal surgery, or even a strenuous workout.   What a Rising CA-125 *Could Mean in Your Context  For an endometrial cancer survivor, it raises concern for: 1. True Cancer Recurrence: This is the primary concern. The recurrence could be local (in the pelvis/vagina) or distant (in the abdomen, lymph nodes, lungs, etc.). 2. A New Primary Cancer: Such as ovarian cancer (especially if your ovaries were preserved) or another abdominal malignancy. 3. One of the benign conditions listed above.  The key is: The CA-125 number alone is not diagnostic. It is a signal to look for the cause.   Your Immediate Action Plan: What to Do Step-by-Step  1. Do Not Panic, But Act Promptly. Contact your gynecologic oncologist or medical oncologist's office immediately. Do not wait for your next scheduled appointment.  2. Provide Specific Information. When you call, be ready to state: "My CA-125 level has risen from [previous number] to [new number] over [timeframe]." Report any new symptoms, no matter how minor (bloating, pain, changes in bowel/bladder habits, vaginal bleeding, an unexplained cough, or fatigue).  3. What Your Oncology Team Will Do: The Standard Workup: Your doctor will not jump to conclusions. They will initiate a careful diagnostic process: Detailed History and Physical Exam: They will review all symptoms and perform a thorough pelvic and abdominal exam. Imaging: This is the most critical next step. You will likely get a CT scan of the chest, abdomen, and pelvis to look for any visible masses or signs of recurrence. A PET-CT scan may be recommended for higher sensitivity. Possible Additional Tests: Depending on symptoms and imaging, they may consider a colonoscopy, upper endoscopy, or specialized tests. Repeat the CA-125: Sometimes, it's repeated to confirm the trend, especially if the rise was small and you have no symptoms.   Important Considerations for Endometrial Cancer: Not All Tumors Secrete CA-125: Many low-grade, early-stage endometrioid cancers do not produce much CA-125. For you, it may not be a useful marker at all. It's more useful for high-risk types: CA-125 is more consistently elevated and monitored for aggressive types like uterine serous carcinoma. The Trend is More Important Than a Single Number: A slow, steady rise over months is more concerning than a single, slightly elevated blip.   Summary & Key Takeaways: A rising CA-125 is a warning light, not a diagnosis. It means "investigate," not "recurrence confirmed." Your job is to report it to your doctor without delay. Their job is to find the cause. The workup will focus on a combination of your symptoms, physical exam, and imaging. The CA-125 guides the search but does not replace it. Stay off "Dr. Google." Interpreting tumor markers without clinical context causes immense and often unnecessary distress.  Prepare for the process: It is a period of high anxiety—the "scanxiety" is real. Lean on your support system, communicate openly with your medical team, and remember that you have a plan. Whether the outcome is a benign condition or a recurrence that needs treatment, knowing is always better than not knowing. It allows you and your team to take the most effective action. |
| How can potential long-term complications be monitored and managed? (e.g., lymphedema, increased cardiovascular disease risk, osteoporosis, etc.) | After endometrial cancer surgery, especially if combined with ovary removal, lymph node dissection, or adjuvant therapy, long-term complications can occur. Monitoring and proactive management are crucial to maintain health and quality of life. Here’s a structured guide:     1. Lymphedema (swelling of the legs or lower body)  Cause: Removal of pelvic/para-aortic lymph nodes can impair lymph drainage.  Monitoring:  Watch for swelling, heaviness, or tightness in the legs, feet, or vulvar area. Early detection is key to preventing progression. Management:  Compression stockings or sleeves, Manual lymphatic drainage therapy (specialized massage), Regular exercise to promote circulation, Avoid injuries, infections, or prolonged standing, and Early referral to a lymphedema specialist if swelling develops.     2. Cardiovascular disease risk  Cause: Surgical menopause (ovary removal) can lead to estrogen deficiency, increasing the risk of:  Heart disease Hypertension Metabolic syndrome  Monitoring:  Blood pressure checks Lipid profile, fasting glucose, HbA1c Weight and waist circumference  Management:  Regular aerobic exercise (walking, cycling) Healthy diet: low saturated fat, high fiber Medications for hypertension, diabetes, or high cholesterol if needed Smoking cessation if applicable     3. Osteoporosis / bone health  Cause: Loss of estrogen accelerates bone loss.  Monitoring:  DEXA scan (bone density) at baseline after surgery, then every 1–2 years, depending on results. Blood calcium and vitamin D levels. Management:  Calcium (1,000–1,200 mg/day) and vitamin D supplementation. Weight-bearing exercise: walking, resistance training. Consider bisphosphonates or other medications if high fracture risk.     4. Vaginal and sexual health  Potential issues: Vaginal dryness, shortening, and pain during intercourse  Monitoring:  Assess vaginal comfort and sexual function at follow-ups Report pain, dryness, or bleeding  Management:  Non-hormonal lubricants and moisturizers, pelvic floor therapy, and low-dose vaginal estrogen in select cases (with oncologist approval)     5. Psychological well-being  Potential issues: Anxiety, depression, body image concerns, and menopausal mood changes  Monitoring:  Routine check-ins during follow-up visits Self-awareness of mood changes, sleep disturbances, or social withdrawal  Management:  Counseling or therapy Support groups for cancer survivors Mindfulness, relaxation exercises, or yoga     6. Secondary cancers or recurrence  Monitoring:  Follow-up schedule with pelvic exams, imaging if indicated, and tumor markers Prompt attention to new symptoms  Management:  Early intervention if recurrence or secondary malignancy is detected Maintain a healthy lifestyle to reduce the overall cancer risk     7. Practical long-term tips: Maintain a personal health record with surgery, pathology, and follow-up details. Schedule regular follow-up visits and lab/imaging tests. Stay active and eat a balanced diet. Report any new symptoms promptly     Bottom line: Long-term complications are preventable or manageable with:  Regular monitoring (lymphedema, bone density, cardiovascular health) Lifestyle measures (exercise, diet, smoking cessation) Medical interventions (compression therapy, supplements, medications) Psychosocial support    I can also make a comprehensive long-term follow-up checklist for the next 5–10 years, including which complications to monitor, what tests to do, and lifestyle recommendations, so you have a concrete plan. This is especially useful if you had ovary removal or lymph node dissection. Do you want me to do that? | This is a critical aspect of survivorship care. Moving beyond cancer surveillance, managing the long-term and late effects of treatment is essential for your overall health and quality of life. Here is a structured guide on monitoring and managing potential long-term complications.   1. Lymphedema (After Lymph Node Removal) What it is: Chronic swelling in the leg(s) due to disruption of the lymphatic system. Monitoring:  Self-Monitoring: Be aware of changes. Measure your ankles/calves periodically with a tape measure. Note feelings of heaviness, tightness, or aching in one leg.  Clinical Monitoring: Report any swelling at your oncology or primary care visits. Management & Prevention:  Prevention: Maintain a healthy weight, practice meticulous skin care to avoid infection, and avoid blood pressure cuffs or injections in the affected leg.  Treatment (if it develops): Referral to a certified lymphedema therapist is key. Treatment includes Manual Lymphatic Drainage (MLD) massage, compression garments, and specialized exercises. It is manageable but not curable.   2. Increased Cardiovascular Disease Risk (Due to Surgical Menopause and Potential Weight Gain) Why: Early loss of estrogen's protective effects, coupled with common risk factors like obesity and hypertension, significantly increases heart disease risk. Monitoring:  Regular Primary Care Visits: Annual checks of blood pressure, cholesterol, and fasting blood glucose.  Awareness of Symptoms: Know the signs of heart disease (chest pain, shortness of breath, palpitations) and stroke (sudden numbness, confusion, vision problems). Management:  Lifestyle: This is the cornerstone. A heart-healthy diet (Mediterranean style), regular aerobic exercise (150 mins/week), smoking cessation, and weight management.  Medical Management: Aggressive control of hypertension, diabetes, and high cholesterol with medications if needed.   3. Osteoporosis & Bone Health (Due to Surgical Menopause) Why: Estrogen is crucial for bone density. Its sudden drop accelerates bone loss. Monitoring:  Bone Density Scan (DEXA Scan): Recommended at menopause (which is immediately post-op if ovaries are removed) and repeated as per your doctor's advice (e.g., every 2–5 years). Management:  Nutrition: Ensure adequate calcium (1,200 mg/day from diet + supplements) and vitamin D (800–2000 IU/day; your level should be checked).  Exercise: Weight-bearing (walking, jogging) and resistance training (lifting weights) are non-negotiable for bone strength.  Medications: If osteoporosis is diagnosed, drugs like bisphosphonates (e.g., alendronate) may be prescribed.   4. Genitourinary Syndrome of Menopause (GSM) What it is: Vaginal dryness, atrophy, pain with intercourse, and urinary symptoms (urgency, recurrent UTIs). Monitoring: Discuss these symptoms openly at follow-up visits. Do not suffer in silence. Management:  First Line: Regular use of vaginal moisturizers (e.g.,, Replens) and lubricants during intimacy.  Most Effective: Low-dose vaginal estrogen (cream, tablet, ring). This has minimal systemic absorption and is generally considered safe and highly recommended for cancer survivors to maintain tissue health and quality of life. Discuss this with your oncologist.   5. Mental Health & Cognitive Changes: "Chemo-brain," anxiety, depression, and fear of recurrence are common. Monitoring: Self-awareness and screening by your doctor. Management: Cognitive-behavioral therapy (CBT), mindfulness, support groups, and, if needed, medications (antidepressants). Don't hesitate to ask for a referral to a psycho-oncologist.   6. Fatigue Monitoring: Track your energy levels. Management: Gradual, consistent exercise is paradoxically the best treatment for cancer-related fatigue. Also, prioritize sleep hygiene and manage other contributing factors (anemia and thyroid function).   Creating Your Personal Survivorship Care Plan: You should receive a Survivorship Care Plan document from your oncology team. It summarizes your treatment and outlines a monitoring schedule. If not, Ask for one.  Your Healthcare Team Will Expand: Gynecologic Oncologist: Focuses on cancer surveillance (years 1–5). Primary Care Physician (PCP): Becomes your quarterback for long-term general health. They manage cardiovascular risk, bone health, and other screenings. Ensure your oncology records are sent to them. Specialists: As needed (cardiology, endocrinology, physical therapy for lymphedema).  Your Action Plan: 1. Schedule an annual wellness visit with your PCP specifically to address these long-term risks. 2. Ask your oncologist: "Can we create a survivorship care plan that addresses lymphedema monitoring, bone health, and cardiovascular risk for me?" 3. Adopt a proactive lifestyle: Nutrition and exercise are your most powerful tools. 4. Communicate: Report any new or worsening symptoms to the appropriate doctor.  By addressing these areas proactively, you take control of your long-term health, aiming not just for survival but for a vibrant and healthy life after cancer. |
| To prevent recurrence, what specific lifestyle changes (diet, exercise, weight management) should I make? | Lifestyle modifications play an important role in reducing recurrence risk after endometrial cancer. While they cannot guarantee prevention, research shows that healthy habits improve overall prognosis, quality of life, and long-term health. Here’s a detailed guide:     1. Weight management: Why it matters:  Obesity and excess abdominal fat increase estrogen levels, which can fuel recurrence in hormone-sensitive tumors.  Recommendations:  Aim for a healthy BMI (18.5–24.9 kg/m²) if possible. Gradual weight loss: 0.5–1 kg per week. Combine a calorie-conscious diet with physical activity.     2. Exercise  Benefits:  Reduces fat tissue and circulating estrogen; improves cardiovascular health, muscle strength, and mood; may reduce fatigue and improve immune function. Guidelines:  Aerobic exercise: 150 minutes/week (e.g., brisk walking, swimming, cycling) Strength training: 2–3 times/week (resistance bands, light weights) Flexibility and balance exercises: yoga or stretching  Tip: Start gradually after surgery—walking is safe early, higher-intensity exercise after 4–6 weeks (or as cleared by your doctor)     3. Diet and nutrition  Focus on:  Plant-based foods: fruits, vegetables, whole grains Lean protein: fish, chicken, legumes Healthy fats: olive oil, nuts, avocado Fiber: supports digestion and weight control  Limit:  Processed foods, refined sugar, and high-fat meats Excess alcohol (increases estrogen and cancer risk)  Other considerations:  Maintain adequate calcium and vitamin D for bone health, especially if ovaries were removed Stay hydrated (water, herbal teas)     4. Avoid tobacco and limit alcohol. Smoking increases the risk of secondary cancers and cardiovascular disease. Alcohol can raise estrogen and affect the liver metabolism of medications.     5. Stress management and sleep  Chronic stress may impact immune function. Adequate sleep (7–9 hours/night) helps recovery and hormone balance. Techniques: meditation, yoga, mindfulness, counseling     6. Regular medical follow-up: Attend all scheduled check-ups. Monitor weight, blood pressure, blood sugar, and lipid profile Report new symptoms promptly     Bottom line  \| Area \| Recommendations \| \| Weight \| Aim for a healthy BMI, gradual weight loss if needed \| \| Exercise \| 150 min/week aerobic + 2–3x/week strength training \| \| Diet \| Plant-based, high fiber, lean protein, healthy fats; limit processed food, sugar, alcohol \| \| Lifestyle \| No smoking, stress management, adequate sleep \| \| Follow-up \| Regular visits, labs, and imaging as advised \|    If you want, I can create a practical daily and weekly lifestyle plan tailored for endometrial cancer survivors, specifying exercise routines, meal ideas, and habit trackers to actively reduce recurrence risk. This gives a concrete structure rather than general advice. Do you want me to do that? | This is one of the most empowering questions you can ask. For endometrial cancer, lifestyle changes are not just general health advice—they are a critical part of your treatment and recurrence prevention plan. The link between body weight, metabolism, and endometrial cancer risk is very strong.  Here are the evidence-based, specific lifestyle changes you should focus on, in order of importance:   1. Weight Management: The Single Most Important Factor Why: Adipose (fat) tissue, especially around the abdomen, produces excess estrogen and creates a state of chronic inflammation and insulin resistance—all of which can fuel the growth of endometrial cancer cells. Goal: Achieve and maintain a healthy BMI (Body Mass Index). Even a 5–10% reduction from your current weight can significantly lower estrogen and insulin levels and reduce recurrence risk. How: Focus on sustainable habits rather than drastic diets. Combine the dietary changes below with consistent physical activity. Consider working with a dietitian who specializes in oncology.   2. Dietary Changes: Adopt an Anti-Inflammatory, Insulin-Sensitizing Diet. Think of food as medicine that can change your internal environment.  Prioritize plant-based, high-fiber foods:  Non-Starchy Vegetables: Fill half your plate with a variety of colorful vegetables (leafy greens, broccoli, peppers, tomatoes).  Whole Fruits: Berries, apples, citrus fruits.  Legumes: Beans, lentils, chickpeas (excellent for fiber and protein).  Whole Grains: Quinoa, oats, brown rice, whole-wheat bread. Choose Lean Protein Sources: Fish (especially fatty fish like salmon, rich in anti-inflammatory omega-3s), skinless poultry, eggs, tofu, and the legumes mentioned above. Limit red and processed meats. Select healthy fats: avocados, nuts, seeds, and olive oil. Avoid trans fats and limit saturated fats. Eliminate sugar-sweetened beverages: This is a quick win. Avoid soda, sweetened coffee drinks, and fruit juices. Drink water, herbal tea, or black coffee. Limit ultra-processed foods: These are high in unhealthy fats, sugars, and salt, and low in fiber. They drive inflammation and weight gain.  The "Mediterranean Diet" pattern is an excellent model to follow.   3. Physical Activity: Move Your Body Regularly. Exercise independently lowers estrogen and insulin levels, reduces inflammation, helps with weight management, and combats fatigue.  Aerobic Exercise: Aim for at least 150 minutes per week of moderate-intensity exercise (e.g., brisk walking, cycling, swimming). This can be broken into 30 minutes, 5 days a week. Strength Training: Include muscle-strengthening activities at least 2 days per week. This is crucial because muscle is metabolically active and improves insulin sensitivity. This can be bodyweight exercises, resistance bands, or weights. Avoid sitting: Break up long periods of sitting. Set a timer to stand and move for 5 minutes every hour.   4. Other Modifiable Factors: Alcohol: Limit or avoid alcohol. It is a source of empty calories, can affect estrogen metabolism, and is a known risk factor for several cancers. Sleep & Stress: Chronic stress and poor sleep can raise cortisol levels, which impacts blood sugar and weight. Prioritize sleep hygiene and stress-reduction techniques (mindfulness, yoga, meditation).   Summary: Your Action Plan for Prevention  1. First Priority: Weight. Aim for steady, gradual weight loss if needed, targeting a 5–10% reduction. 2. Transform Your Plate: Build meals around plants and lean protein. Make vegetables the star. 3. Create an Exercise Routine: Combine cardio and strength training. Consistency is more important than intensity. 4. Seek Support: Ask your oncologist for a referral to a dietitian/nutritionist and potentially a physical therapist or certified fitness trainer familiar with cancer recovery. 5. Be Patient and Kind to Yourself: This is a marathon, not a sprint. Focus on progress, not perfection. Every healthy choice is a positive step.  Crucial Note: These lifestyle changes complement your medical follow-up and any recommended adjuvant therapies; they do not replace them. They are a powerful way for you to take an active role in your long-term health and significantly improve your odds of remaining cancer-free.  You have the power to positively influence your internal environment. This knowledge is your tool. |
| Can I receive vaccines such as the HPV vaccine or the COVID-19 vaccine? | Yes, in most cases, you can receive vaccines like HPV and COVID-19 after endometrial cancer treatment, but timing and type matter. Here’s a detailed explanation:     1. HPV vaccine  Purpose: Protects against human papillomavirus, which can cause cervical, vaginal, vulvar, and other cancers. After endometrial cancer:   The vaccine does not treat existing endometrial cancer.  It may still provide protection against other HPV-related cancers. Eligibility: Usually safe for adults up to 45 years old. Timing: Can be given after recovery from surgery, typically once your immune system is stable and you are not on chemotherapy.     2. COVID-19 vaccine  Purpose: Protects against COVID-19 infection, which can be more severe in cancer patients. After surgery or during follow-up:   Safe for most patients once recovered from major surgery.  If you are on chemotherapy, immunotherapy, or high-dose steroids, your response may be lower, but vaccination is still recommended. Timing:   Preferably after surgical wounds have healed and you are clinically stable.  Avoid vaccination on the same day as major surgery.     3. Other vaccines: Influenza (flu) vaccine – recommended annually. Pneumococcal vaccine – recommended for adults with comorbidities or older age. Td/Tdap booster – follow the standard adult schedule.  General rules:  Prefer inactivated vaccines (not live vaccines) if you are immunocompromised. Space vaccines away from chemotherapy sessions if applicable. Discuss with your oncologist or primary doctor before starting any new vaccine.     4. Practical tips: Keep a vaccination record. Inform the clinic about your cancer history and any current treatment. Monitor for side effects, especially after surgery or during chemotherapy.    Bottom line:  HPV vaccine: may provide protection against other HPV-related cancers; safe after recovery. COVID-19 vaccine: safe after surgery; ideally after wound healing and outside chemotherapy days. Other inactivated vaccines are generally safe. But timing may need adjustment based on treatment.  If you want, I can make a post-endometrial cancer vaccination schedule, showing which vaccines are recommended, optimal timing, and special considerations. This helps ensure safety and effectiveness. Do you want me to do that? | This is a very important and practical question. The answer is: Generally, yes, and for cancer survivors, receiving certain vaccines is crucial. However, the timing and types of vaccines should be decided in consultation with your oncologist based on your specific situation.  Here is a detailed analysis of two key vaccines:   1. COVID-19 Vaccine Conclusion: Strongly recommended and a priority. Why is it possible and advisable? Cancer patients (including those undergoing treatment and survivors) are at high risk for severe illness, critical condition, and death from COVID-19 infection. Vaccination is the most effective means of preventing severe disease, hospitalization, and death. Timing is crucial (must be confirmed with your oncologist):  During chemotherapy: It is generally recommended to receive the vaccine at the end of a chemotherapy cycle or during the recovery period between cycles, when the immune response may be better. Avoid vaccination when white blood cell counts are at their lowest.  During radiotherapy: Usually possible, but it's best to avoid the treatment area (e.g., pelvic radiotherapy).  During immunotherapy: Vaccination is generally safe for patients on immune checkpoint inhibitors (e.g., Pembrolizumab), but requires physician assessment.  Post-surgery/Observation period: Vaccination can proceed once the body has largely recovered from major surgery. Vaccine type: mRNA vaccines (e.g., Pfizer, Moderna) or recombinant protein vaccines are recommended due to their favorable safety and efficacy data even in immunocompromised individuals. Completion of the primary series and subsequent booster doses is advised.   2. HPV (Human Papillomavirus) Vaccine Conclusion: Vaccination is possible, but its primary value lies in "preventing future new infections," not in treating existing cancer. Why is it possible? While most endometrial cancers are not related to HPV infection, a small subset (particularly certain special types) may be associated. More importantly, as a cancer survivor, Receiving the HPV vaccine can prevent other HPV-related cancers, such as cervical, vaginal, vulvar, oropharyngeal, and anal cancers. Who is most suitable?  Younger patients (within national age limits, typically ≤45 years) benefit the most.  Even with a prior HPV infection or a history of an HPV-related cancer, the vaccine can still protect against other HPV subtypes not yet encountered. Timing: It is generally recommended to receive the vaccine after cancer treatment is completed and your physical condition is stable. Again, consult your doctor to determine the optimal start time.   General Principles for Other Vaccines Inactivated vaccines (e.g., influenza, pneumococcal, and hepatitis B vaccines): Generally safe and recommended. Cancer patients are at high risk for influenza and pneumonia, making annual flu vaccination very important. Live attenuated vaccines (e.g.,, MMR, varicella, shingles vaccines): Extreme caution is required! Administration is contraindicated during periods of potential immune suppression (e.g., active chemotherapy, radiotherapy, or high-dose immunosuppressant use) due to the risk of disease caused by the replication of the vaccine virus. They may only be considered after treatment has ended for a sufficient duration and immune function has fully recovered, following strict assessment by a physician.   Your Action Plan 1. Discuss this with your primary oncologist: This is a mandatory step before receiving any vaccine. Inform your doctor of your complete treatment history and current status. 2. Develop a vaccination schedule: Work with your doctor to create a safe vaccination timeline based on your treatment milestones (e.g., chemotherapy cycles). 3. Prioritize vaccines: The typical order of priority is COVID-19 vaccine > influenza vaccine > pneumococcal vaccine > HPV vaccine. 4. Inform the vaccine provider: When visiting a community vaccination site, Be sure to tell the healthcare provider that you are a cancer survivor/patient and what treatments you are currently receiving or have received.  In summary: Vaccination is an important component of your long-term health management. For vaccines like those against COVID-19 and influenza, the benefits of vaccination far outweigh the potential risks. By working closely with your healthcare team to choose the correct timing and vaccine types, you can safely build an important protective immune barrier for yourself. |
| Will my disease and treatment have long-term effects on my bone health (osteoporosis), cardiovascular health, and cognitive function? How can these be addressed? | Yes, endometrial cancer treatment—especially surgery that removes the ovaries, adjuvant therapy, or induced menopause—can have long-term effects on bone, cardiovascular, and cognitive health. Here’s a clear breakdown and strategies to address them:     1. Bone health (osteoporosis risk)  Causes:  Surgical menopause → sudden loss of estrogen, which protects bones Chemotherapy or long-term steroid use can worsen bone density  Potential consequences:  Osteopenia → osteoporosis Increased fracture risk (spine, hip, wrist)  Monitoring:  DEXA scan (bone density) baseline after surgery, then every 1–2 years depending on results Monitor calcium and vitamin D levels  Management:  Calcium: 1,000–1,200 mg/day Vitamin D: 800–1,000 IU/day Weight-bearing exercise: walking, dancing, resistance training Medications: bisphosphonates or denosumab if high fracture risk Avoid smoking and excessive alcohol     2. Cardiovascular health  Causes:  Loss of estrogen accelerates atherosclerosis, increases blood pressure, and impacts lipid metabolism Obesity, inactivity, or chemotherapy may further increase risk  Monitoring:  Blood pressure, cholesterol, glucose, and HbA1c regularly Weight, waist circumference, and cardiovascular symptoms  Management: A healthy diet: a Mediterranean-style diet rich in vegetables, fruits, lean protein, and healthy fats Regular exercise: at least 150 min/week of moderate activity Medications for hypertension, hyperlipidemia, or diabetes if needed Avoid smoking     3. Cognitive function ("chemo brain" or menopause-related cognitive changes)  Potential effects:  Difficulty concentrating, memory lapses, and slowed processing speed Can be caused by chemotherapy, hormonal changes, sleep disturbances, or stress  Monitoring:  Self-assessment of memory, focus, and multitasking Discuss concerns at follow-up visits  Management:  Cognitive exercises: puzzles, reading, and memory games Physical activity: improves brain blood flow and mood Sleep hygiene and stress management Address mood disorders: counseling or therapy if anxiety/depression are present     4. Practical tips for long-term health: Keep a personal health log: medications, labs, imaging, and lifestyle habits Schedule regular check-ups with primary care and oncology Adopt preventive lifestyle habits: healthy weight, diet, exercise, no smoking, and moderate alcohol Discuss hormone replacement therapy cautiously (rarely used in endometrial cancer) Consider referrals: endocrinologist for bone health, cardiologist for heart risk, and cognitive therapist or neuropsychologist if cognitive concerns arise     Bottom line  Bone health: at risk from estrogen loss; prevent with calcium, vitamin D, exercise, and monitoring Cardiovascular health: at risk; prevent with lifestyle, regular labs, and medications if needed Cognitive function: may be affected by treatment and menopause; improve with mental exercises, sleep, and stress management Proactive monitoring and early interventions greatly reduce long-term complications    If you want, I can create a long-term health monitoring and lifestyle plan for endometrial cancer survivors, showing which tests, exercises, and preventive steps to take each year, so you have a structured approach to maintain bone, heart, and brain health. This can make it much easier to manage post-treatment. Do you want me to do that? | This is a profoundly important question. The answer is: Yes, it can. Endometrial cancer itself (particularly its associated metabolic issues) and its treatments—especially surgical removal of the ovaries leading to premature menopause, and potentially chemotherapy—can indeed have significant long-term impacts on your health. Please be assured, however, that these effects are well understood, and there are clear strategies for prevention and management. The goal of modern cancer care is not only to cure the disease but also to safeguard your long-term quality of life.  Below is a detailed analysis and management plan for these three key areas:   1. Bone Health: Significantly Increased Risk of Osteoporosis: Cause: Surgical removal of the ovaries (or ovarian failure due to chemotherapy) leads to a sharp and permanent decline in estrogen levels. Estrogen is a crucial hormone for maintaining bone density. Impact: Accelerated bone loss, leading to a markedly increased risk of fractures (particularly in the hip, spine, and wrist) within just a few years after treatment. Management:  Assessment: A baseline bone density test (DXA scan) should be performed after menopause or treatment completion for future comparison. Follow-up scans should be conducted periodically (e.g., every 2–5 years) based on risk.  Lifestyle Interventions:  Supplements: Ensure adequate intake of calcium (1200 mg daily, ideally from dietary sources, supplemented if insufficient) and vitamin D (800–2000 IU daily; blood levels should be checked to ensure sufficiency).  Exercise: Weight-bearing exercises (e.g., brisk walking, jogging) and resistance training (e.g., lifting weights, using resistance bands) are crucial for stimulating bone growth.  Medical Intervention: If osteoporosis or high risk is confirmed by bone density testing, medications such as bisphosphonates (e.g.,, alendronate) or other bone resorption inhibitors/formation promoters may be prescribed.   2. Cardiovascular Health: A Risk That Cannot Be Overlooked Causes:  1. Loss of Estrogen's Protective Effect: Estrogen is cardioprotective in women. Its premature loss increases the risk of atherosclerosis and hypertension.  2. Shared Risk Factors: Endometrial cancer is closely linked to metabolic syndrome components like obesity, insulin resistance, diabetes, and hypertension, which are themselves major risk factors for cardiovascular disease.  3. Treatment Effects: Certain chemotherapy drugs (e.g., anthracyclines) may have potential effects on heart muscle. Management:  Monitoring: Integrate cardiovascular health into routine follow-up care. Annual checks of blood pressure, lipid profile (cholesterol, triglycerides), and fasting blood glucose are essential.  Active Risk Factor Management:  Weight Control: This is the most critical component.  "Heart-Healthy" Diet: Adopt a Mediterranean-style diet rich in vegetables, fruits, whole grains, healthy fats (olive oil, nuts), and fish, while limiting saturated fats, trans fats, and processed foods.  Regular Exercise: At least 150 minutes of moderate-intensity aerobic exercise per week is one of the best ways to improve metabolism and protect the heart.  Strict smoking cessation and alcohol moderation.  Medical Management: If hypertension, dyslipidemia, or diabetes develops, prompt pharmacological control under the guidance of an internal medicine physician is crucial, often with stricter treatment targets than for the general population.   3. Cognitive Function: "Chemo Brain" and Menopausal Effects Causes:  1. Treatment-Related: Chemotherapy can cause short-term or even long-term cognitive changes, often called "chemo brain," manifesting as memory loss, difficulty concentrating, and slowed thinking.  2. Hormonal Shift: Estrogen helps regulate cognitive function. Its sudden decline can affect memory, mood, and sleep.  3. Psychological Factors: The stress of illness, anxiety, depression, and fatigue themselves can significantly impair cognition. Management:  Acknowledgment and Communication: First, recognize these as real symptoms, not "overthinking." Communicate your feelings with your doctors and family.  Cognitive Rehabilitation & Training:  Use aids like notebooks, calendars, and phone reminders.  Engage in mentally stimulating activities like reading, puzzles, or learning new skills.  Try mindfulness meditation to improve focus.  Addressing Root Causes: Actively treat insomnia, anxiety, and depression. Improving these conditions often significantly enhances cognitive function.  Staying Socially and Mentally Active: Engaging in social interactions and beneficial mental activities helps build cognitive reserve.   Integrated Management: Your "Survivorship Care Plan": You need a multidisciplinary team to address these concerns: Gynecologic Oncologist: Responsible for cancer follow-up and initiating discussions about long-term risks. Primary Care/Family Physician: Acts as the "gatekeeper" for your long-term health, coordinating and managing chronic disease risks like cardiovascular and bone health. Specialists: Referral to cardiologists, endocrinologists, rehabilitation specialists, or mental health professionals as needed.  You can proactively request a "Survivorship Care Plan" from your oncologist. This document should summarize your treatment history and outline recommended monitoring and management strategies for these long-term health risks.  In summary: Yes, these long-term effects are real challenges after treatment, but this does not mean you must accept them passively. Through proactive monitoring, a healthy lifestyle, and timely medical intervention when necessary, you can significantly mitigate these risks, maintain good physical and cognitive function, and enjoy a high quality of life for years to come. Please place equal importance on managing these issues as you do on your cancer follow-up care. |
| If recurrence or metastasis occurs in the future, what effective treatment options remain? Will current treatments affect future options? | If endometrial cancer recurs or metastasizes, there are still several effective treatment options, but your initial treatment and pathology features influence what is feasible and effective. Here’s a detailed overview:     1. Recurrence patterns: Local recurrence: usually in the vaginal cuff or pelvis Regional recurrence: lymph nodes in the pelvis or para-aortic region Distant metastasis: lungs, liver, peritoneum, or other organs  Treatment depends on:  Location of recurrence Tumor biology (grade, histology, molecular markers) Prior treatments (surgery, radiation, chemotherapy) Overall health and comorbidities     2. Treatment options for recurrence: a. Surgery—Possible if recurrence is localized and resectable; previous radiation or surgery may make repeat surgery more complex  b. Radiation therapy—External beam or brachytherapy can be used for localized pelvic recurrences. Prior radiation may limit re-irradiation options due to tissue tolerance. Chemotherapy—Platinum-based regimens (carboplatin + paclitaxel) are standard. Other agents (doxorubicin, paclitaxel, ifosfamide) may be used depending on prior therapy. Previous chemotherapy may limit cumulative dosing and options. Hormone therapy—For low-grade, hormone receptor-positive tumors, options include progestins, aromatase inhibitors, and selective estrogen receptor modulators. These are often used when the disease is slow-growing or systemic. Targeted therapy/immunotherapy—Molecular profiling of the recurrent tumor is important. Options include:   PD-1/PD-L1 inhibitors (for MSI-high or mismatch repair-deficient tumors)  mTOR inhibitors or other targeted agents depending on mutations. Availability depends on prior treatments and molecular status.     3. How current treatments affect future options: Prior radiation: limits the dose for re-irradiation Prior chemotherapy: limits the cumulative dosing of certain drugs due to toxicity (heart, nerves, bone marrow) Ovary removal or hormone therapy: may influence eligibility for hormonal strategies Surgery: Previous extensive surgery may make reoperation more complex. Bottom line: treatment choices are influenced by what has already been done, tumor characteristics, and your overall health.     4. Key considerations for patients: Early detection of recurrence improves outcomes; follow-up is essential. Molecular testing of recurrent tumor can expand targeted therapy options. Multidisciplinary care (gynecologic oncology, medical oncology, radiation oncology) is recommended. Even with recurrence, long-term control and quality of life are achievable with appropriate therapy    If you want, I can make a diagram showing first-line, second-line, and targeted treatment options for recurrence, including how prior treatments influence these choices, so you can visualize your future options clearly. This can help reduce anxiety and plan ahead. Do you want me to do that? | This is a critical issue that requires courage to confront. First and foremost, please be clear: Even if recurrence or metastasis occurs in the future, it absolutely does not mean there are no options left. The field of endometrial cancer treatment is advancing rapidly, particularly with targeted and immunotherapy, offering new and effective choices for patients with recurrent disease.  The initial primary treatment (surgery, radiation, chemotherapy) will influence future options, but this typically means strategically selecting the next line of therapy, not that all methods have been "used up."  Below is a comprehensive overview of treatment options after recurrence or metastasis, along with an explanation of how current treatments affect future choices.   What are the effective treatment options if recurrence/metastasis occurs?  The choice of treatment plan depends on three core factors: 1) the site and extent of recurrence; 2) the time elapsed since primary treatment; and 3) the tumor's molecular characteristics (most crucial).  # Category One: Local/Regional Recurrence - Scenario: Recurrence confined to the pelvis, vagina, or lymph nodes. - Treatment Options:  - Surgical Resection: If completely resectable, this remains the preferred option.  - Radiation Therapy: Highly effective if prior radiotherapy was not received or can be delivered to a new area.  - Combined Chemoradiation: For more extensive regional recurrence.  # Category Two: Distant Metastasis (Systemic Recurrence) This is a more common scenario, requiring systemic therapy. Treatment has entered the era of "precision classification and tailored treatment."  1. Hormone Therapy (Applicable for low-grade, estrogen/progesterone receptor-positive tumors) - Applicable Scenario: Slow progression, asymptomatic or mild bone/lung metastases. - Drugs: High-dose progestins (e.g., Megestrol acetate), aromatase inhibitors (e.g., Anastrozole), Tamoxifen, etc. - Advantages: Oral administration, relatively mild side effects.  2. Chemotherapy - Foundation Regimen: Carboplatin + Paclitaxel remains the first-line chemotherapy for most recurrent patients. It can often be reused even if employed in initial adjuvant therapy, especially with a longer treatment-free interval (>6–12 months). - Other Regimens: Doxorubicin, Docetaxel, Topotecan, etc., as subsequent options.  3. Targeted Therapy & Immunotherapy (Revolutionary Advances) This is currently the most dynamic area, with efficacy depending on your tumor's molecular subtype (determined via genetic testing): - For dMMR/MSI-H (Mismatch Repair Deficient / Microsatellite Instability-High) Tumors:  - Immune Checkpoint Inhibitors (e.g., Pembrolizumab): Used alone or combined with chemotherapy, showing excellent efficacy and becoming the first choice for these patients. - For p53-mutant tumors (e.g., serous carcinoma) or specific gene mutations:  - Anti-angiogenic Agents (e.g., Bevacizumab): Combined with chemotherapy, can extend progression-free survival.  - PARP Inhibitors (e.g., olaparib): Combined with chemotherapy, can improve overall survival., Olaparib): Effective for tumors with homologous recombination deficiency (e.g., BRCA mutations).  - Antibody-Drug Conjugates (ADCs): Such as Trastuzumab deruxtecan, showing significant efficacy against HER2-positive tumors (found in some high-grade types).  4. Clinical Trial Participation: For patients who have exhausted standard treatments or wish to access the latest therapies, clinical trials offer opportunities for cutting-edge drugs (e.g., novel ADCs, bispecific antibodies, cell therapies, etc.).   How does current primary treatment affect future choices?  This influence is "stepwise" and "personalized," not simply "blocking" future paths.  1. Impact of Radiation Therapy:  - If the pelvis received a full dose of radiation during primary treatment, the same area typically cannot receive curative radiotherapy again. However, radiation is a local modality and does not affect the choice of systemic therapies (e.g., chemotherapy, targeted drugs).  2. Impact of Chemotherapy:  - Affects the selection of subsequent chemotherapeutic agents: If recurrence occurs soon after initial adjuvant chemotherapy (<6 months), it suggests potential resistance to the prior regimen. The next chemotherapy should utilize drugs with different mechanisms (e.g., switching from platinum-based to doxorubicin, etc.).  - Does not affect targeted/immunotherapy: This is key! Regardless of prior chemotherapy, if the tumor has the corresponding molecular marker (e.g., dMMR), it can benefit from immunotherapy. Targeted/immunotherapy opens entirely new pathways independent of chemotherapy resistance.  3. Impact of Surgery:  - Successful surgical debulking provides a foundation and does not affect |

The specific time window for data collection (December 10–20, 2025).
